# Pho84/GLUT2 are cellular *myo*-inositol exporters regulated by inositol pyrophosphates

**DOI:** 10.1038/s44318-026-00888-9

**Published:** 2026-08-07

**Authors:** Xue Bessie Su, Xinjie Zhang, Valeria Fedeli, An-Li Andrea Ko, Jiarui Nicole Fu, Dorothea Fiedler, Adolfo Saiardi

**Affiliations:** 1 Laboratory for Molecular Cell Biology (LMCB), University College London (UCL), London, WC1E 6BT UK; 2 Leibniz-Forschungsinstitut für Molekulare Pharmakologie (FMP), Robert-Rössle-Straße 10, 13125 Berlin, Germany

**Keywords:** Metabolism, Signal Transduction

## Abstract

Inositol is an essential nutrient for most living organisms, as combinatorial phosphorylation on this cyclic sugar generates key cellular messengers, such as lipid-bound phosphoinositides (PtdInsPs), water-soluble inositol phosphates (InsPs), and high-energy inositol pyrophosphates (PP-InsPs). Although the kinases and phosphatases modifying inositol-derived molecules are well-characterised, the molecular pathways controlling the cellular homeostasis of the inositol backbone and transport carriers remain unclear. Using a combination of LC-MS analysis and a screen based on the inositol-exporting *opi1Δ* mutant yeast, we here discovered that inositol export is tightly regulated by PP-InsPs and that the high-affinity phosphate transporter Pho84 also acts as an inositol exporter. We further expanded these observations to the mammalian system and revealed that inositol export in human cells is similarly controlled by PP-InsPs, and that the human homolog of the yeast Pho84, GLUT2, contributes to inositol export. In summary, we discovered an evolutionarily conserved crosstalk pathway linking PP-InsPs to both phosphate and inositol homeostasis.

## Synopsis

Inositol provides the backbone for phosphoinositide and inositol phosphate signalling molecules, yet how cells regulate their pool of free inositol remain ill-defined. This study identifies yeast Pho84 and its human homologue GLUT2 as inositol exporters and uncovers inositol pyrophosphates as novel regulators of inositol homeostasis.

- Inositol pyrophosphates (PP-InsPs) regulate the cellular exit of inositol.
- The high-affinity phosphate transporter Pho84 mediates cellular inositol export in yeast, balancing between phosphate and inositol levels.
- Regulation of inositol export by PP-InsPs is evolutionarily conserved in mammalian cells.
- The low-affinity glucose transporter GLUT2, which is the mammalian homologous of Pho84, functions as an inositol exporter.

## Abstract (web summary)

An evolutionarily conserved transport circuit links inositol pyrophosphate signalling to cellular inositol and phosphate homeostasis.

## Graphical abstract

**Figure:**
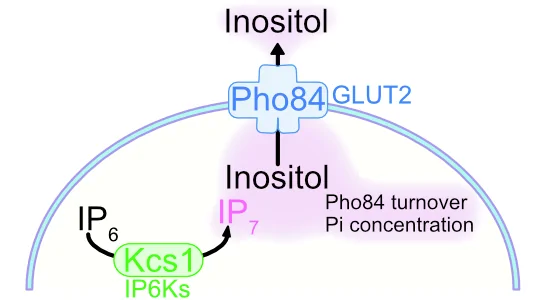

## Introduction

The cyclitol sugar *myo*-inositol (hereafter inositol) is an essential metabolite across all three domains of life (Michell, 2008). Inositol, or its dimeric form in archaea, functions as an organic osmolyte (Su et al, 2023). Eukaryotes have further exploited the metabolic stability of inositol by phosphorylating its six hydroxyl groups to generate a vast repertoire of metabolites, such as inositol phosphates (InsPs) (Kim et al, 2024) and their membrane-anchored derivatives, the phosphoinositides (PtdInsPs) (Balla, 2013; Hammond and Burke, 2020). InsPs and PtdInsPs collectively form a highly dynamic signalling network central to eukaryotic physiology. In addition, diphospho-inositol derivatives, also known as inositol pyrophosphates (PP-InsPs), are commonly present in eukaryotic cells (Nguyen Trung et al, 2022). Over the past several decades, extensive research has been dedicated to the characterisation of the kinases and phosphatases governing the synthesis and turnover of PtdInsPs, InsPs, and PP-InsPs. In contrast, much less work has been done to understand the synthesis, acquisition, and turnover of inositol itself. Given that inositol serves as the backbone of PtdInsPs, InsPs, and PP-InsPs, its homoeostatic regulation may represent a fundamental layer of control over the diverse signalling functions of these molecules. Additionally, inositol homoeostasis is central to the ‘inositol depletion hypothesis’, which underpins the proposed mechanism of lithium’s mood-stabilising effects (Berridge et al, 1989; Dollins et al, 2021; Saha et al, 2024).

Inositol homoeostasis in eukaryotic cells is controlled by four mechanisms: de novo synthesis, acquisition from the extracellular environment, utilisation to synthesise inositol-containing metabolites and export from the cell. It is important to emphasise that although inositol degradation pathways are commonly found in eubacteria, they are largely absent from eukaryotic cells (Su et al, 2023). Inositol de novo synthesis from the glycolytic intermediate glucose-6-phosphate (glucose-6P) involves two steps. First, inositol-3-phosphate synthase (Ino1 in yeast, ISYNA1 in mammals) catalyses the cyclisation of glucose-6P to generate inositol-3-phosphate (Ins3P), using NADH as a cofactor. Inositol monophosphatases (Inm1/2 in yeast, IMPA1/2 in mammals) subsequently dephosphorylate Ins3P to generate inositol. The presence of Ino1 homologues, and consequently the capacity to synthesise inositol, are common amongst archaeal and eukaryotic species (Michell, 2008), but are absent from many groups of eubacteria. The budding yeast *Saccharomyces cerevisiae* played a pivotal role in uncovering the enzymology of inositol synthesis, with *INO1* (INOsitol-requiring) first identified in a yeast mutagenesis screen (Culbertson and Henry, 1975). Subsequent studies revealed that *INO1* transcription is downregulated in the presence of exogenous inositol. *INO1* transcription is regulated by transcriptional repressor Opi1 (OverProducer of Inositol), and the *opi1Δ* mutant overproduces inositol, leading to its export from the cell (Greenberg et al, 1982).

A recent study suggests that Kcs1, the yeast IP_6_-kinase that generates the PP-InsPs InsP_7_, is a positive regulator of *INO1* transcription (Ye et al, 2013). Interestingly, Kcs1 and the downstream Vip1, which produces InsP_8_ (also known as PP-InsP_5_ and PP_2_-InsP_4_), also regulate phosphate homoeostasis through distinct pathways (Azevedo and Saiardi, 2017; Saiardi, 2012) (Dollins et al, 2020; Saiardi et al, 1999). Vip1 regulates the PHO regulon, a gene network activated under phosphate starvation (Lee et al, 2007). In the absence of Vip1, the cyclin–cyclin-dependent kinase (CDK) complex Pho80–Pho85 is constitutively active, triggering persistent phosphorylation of the transcription factor Pho4 and its retention in the cytoplasm. As a result, phosphate-responsive genes such as *PHO84*, which encodes the high-affinity phosphate transporter, are repressed, rendering *vip1Δ* yeast unresponsive to phosphate starvation (Chabert et al, 2023). Kcs1, on the other hand, directs the synthesis of inorganic polyphosphate (polyP) (Wild et al, 2016), the principal phosphate storage polymer in yeast. *kcs1Δ* mutant also causes constitutive nuclear localisation of Pho4, thus triggering a phosphate starvation–like response even under phosphate-replete conditions (Chabert et al, 2023).

Inositol uptake from the extracellular environment is mediated by transporters such as the sodium-dependent myo-inositol transporters (SMIT1 and SMIT2) and the proton-dependent myo-inositol transporter (HMIT) in human cells (Vawter et al, 2019). In budding yeast, the inositol transporter Itr1 and its paralog Itr2 are homologous to HMIT. In contrast, little is known about the mechanisms governing inositol export, despite it being a characterised phenomenon in *opi1Δ* yeast (Greenberg et al, 1982). The concept of an inositol export pathway has received even less attention in human physiology, although it may be critical for transporting inositol across the intestinal epithelial cells, the placenta to supply the foetus and the blood–brain barrier (Su et al, 2023). Lithium and valproate have been used to treat bipolar disorder by reducing the cellular pool of free inositol, but patients show variable responses to such treatments (Herrera-Rivero et al, 2024), which could be accounted for by genetic variations in pathways controlling inositol homoeostasis, including inositol export. Importantly, SMIT1/2 and HMIT/Itr1-2 are unlikely to function in reverse, as this would require transporting against the sodium or proton gradients. This fundamental gap in the understanding of inositol homoeostasis thus prompted us to identify the inositol exporter(s) and the regulatory mechanisms. Using *opi1Δ* yeast as a model system, we demonstrated that inositol export is tightly regulated by PP-InsPs and that the high-affinity phosphate transporter Pho84 also acts as a yeast inositol exporter. We further showed that the regulatory pathway of inositol export is conserved in mammalian cells, with the human low-affinity glucose transporter GLUT2 capable of exporting inositol. This work therefore reveals a previously unknown crosstalk network connecting PP-InsP, phosphate metabolism and inositol homoeostasis.

## Results

### Kcs1 regulates inositol homoeostasis

**Figure 1:**
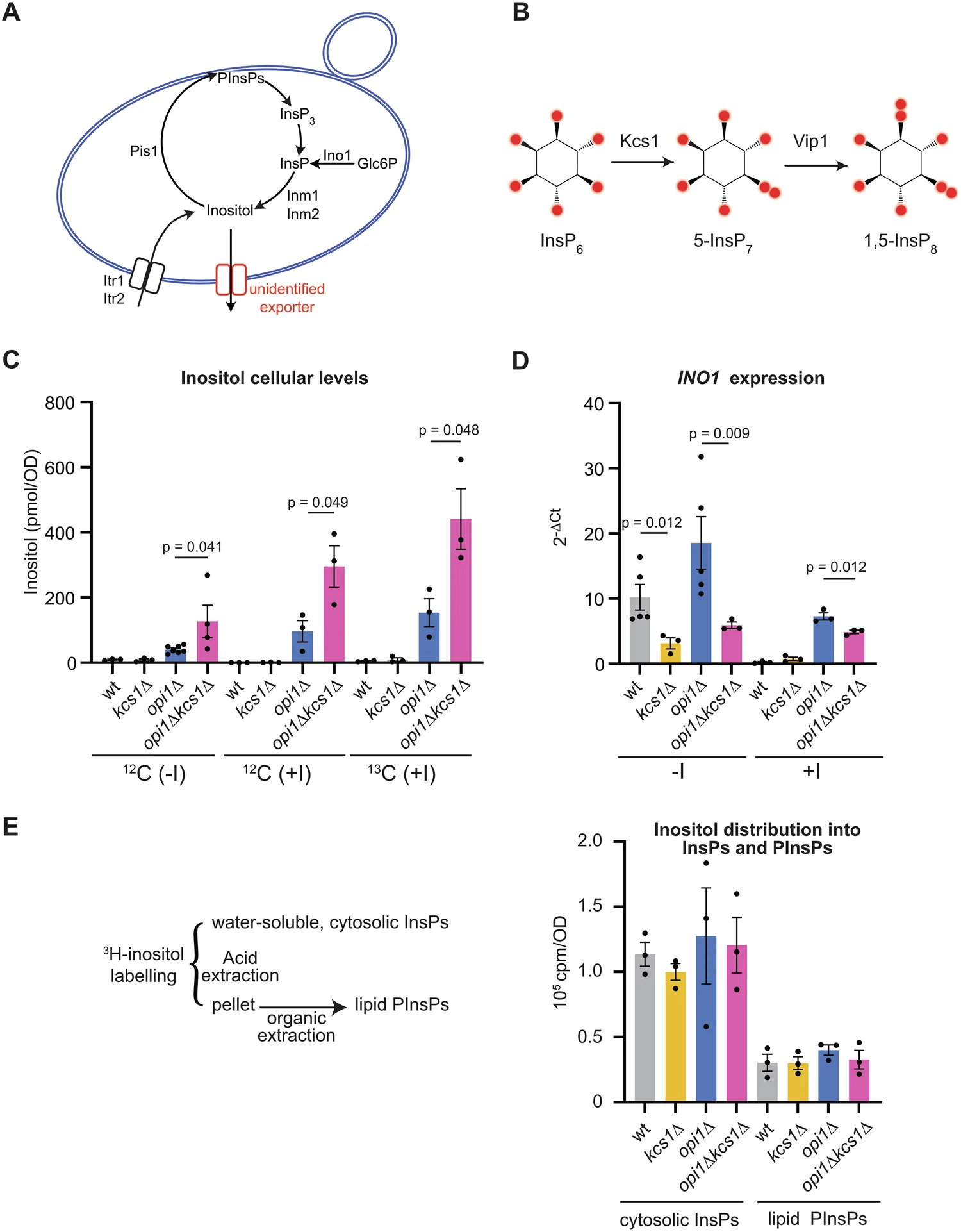
Inositol levels are maintained in *kcs1Δ* yeast despite downregulation of *INO1* expression. (**A**) Schematics of pathways controlling intracellular levels of inositol. Exogenous inositol is acquired by the cell via importers Itr1 and Itr2. Ino1, the yeast homologue of ISYNA1, synthesises inositol monophosphate (InsP) from glucose-6-phosphate (Glc6P). Inm1 and Inm2, the yeast functional orthologs of IMPA1, subsequently dephosphorylate InsP to generate free inositol. Free inositol could either be used by phosphatidylinositol synthase (Pis1) to synthesise phosphoinositides (PtdInsPs) or exported outside the cell via unknown transporters. (**B**) PP-InsPs are synthesised by Kcs1 and Vip1 kinases. (**C**) Intracellular inositol levels remain unchanged in *kcs1Δ* and is increased in *opi1Δkcs1Δ*. Wild-type W303 (yBS1), *kcs1Δ* (yBS2), *opi1Δ* (yBS45) and *opi1Δkcs1Δ* (yBS186) yeast cells were grown to mid-log phase in SC-ino (-I) or +10 µM ^13^C_6_-inositol (+I) media. ^12^C- and ^13^C_6_- inositol, hence represented endogenously synthesised and exogenously acquired inositol, respectively. Sugar extraction and LC-MS analysis were performed as described in Methods. Graphs represent averages ± standard error (s.e.m) (*n* ≥ 3 biological replicates, *opi1Δ* ^12^C -I vs. *opi1Δkcs1Δ* ^12^C -I: *P* = 0.041, *opi1Δ* ^12^C + I vs. *opi1Δkcs1Δ* ^12^C + I: *P* = 0.049, *opi1Δ* ^13^C vs. *opi1Δkcs1Δ* ^13^C: *P* = 0.048, using two-tailed Student’s *t*-test). (**D**) *INO1* expression is reduced when *KCS1* is deleted. Yeast cells were grown as described in Fig. 1C. RNA extraction and RT-qPCR were performed as described in Methods. Ct values of each gene were normalised to that of *TUB1* (loading control) using the 2^−ΔCt^ method. Graphs represent averages ± s.e.m (s.e.m) (*n* ≥ 3 biological replicates, wt -I vs. *kcs1Δ* -I: *P* = 0.012, *opi1Δ* -I vs. *opi1Δkcs1Δ* -I: *P* = 0.009, *opi1Δ* + I vs. *opi1Δkcs1Δ* + I: *P* = 0.012, comparing ln(2^−ΔCt^) values using two-tailed Student’s *t*-test.). (**E**) Bulk levels of water-soluble or lipid-associated inositol-containing metabolites are similar in wt, *kcs1Δ*, *opi1Δ* or *opi1Δkcs1Δ* yeast. Experimental workflow is depicted on the left. Briefly, yeast cells were labelled with 1 µM ^3^H-inositol for at least five cell divisions. Cells were subject to perchloric acid extraction, and the insoluble materials were further subject to organic extraction. Radioactivity was counted after each extraction to measure the amount of water-soluble or lipid-associated metabolites containing ^3^H-inositol. Graphs represent averages ± s.e.m (*n* = 3 biological replicates, using two-tailed Student’s *t*-test). Source data are available online for this figure.

**Figure EV1:**
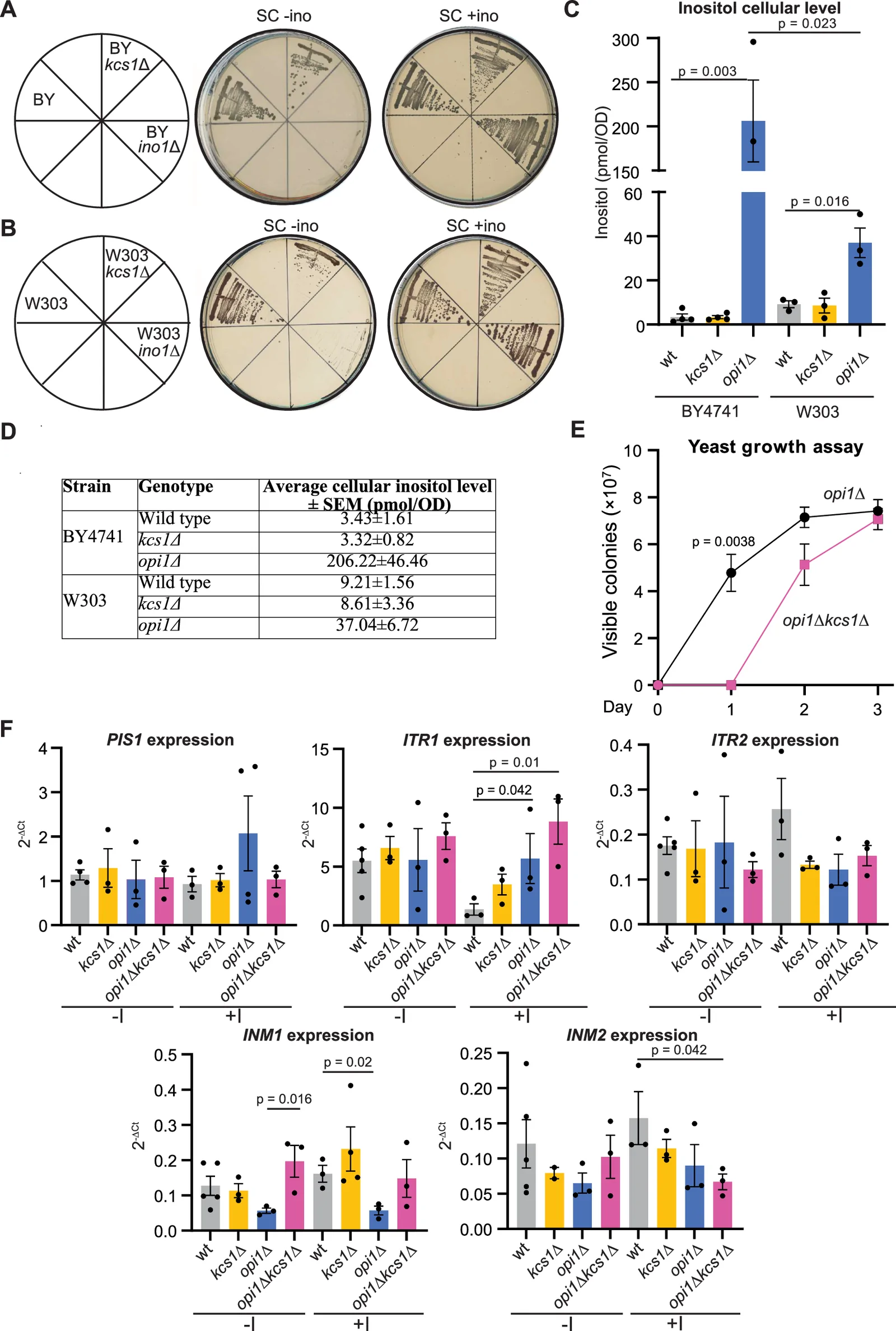
Phenotypic evaluation of *kcs1Δ* and *opi1Δkcs1Δ* yeast. (**A**) Backcrossed BY4741 (abbreviated as BY) *kcs1Δ* yeast is inositol prototrophic. Wild-type (yXZ3), *kcs1Δ* (yXZ4) and *ino1Δ* (yXZ5) yeast cells were streaked on synthetic complete media with or without 10 µM *myo*-inositol (abbreviated as SC-ino or SC+ino), as indicated in the schematics. (**B**) W303 *kcs1Δ* yeast is inositol prototrophic. Wild-type W303 (yBS1), *kcs1Δ* (yBS2) and *ino1Δ* (yBS88) yeast cells were streaked on SC-ino or SC+ino, as indicated in the schematics. (**C**) Intracellular inositol levels are unchanged in the *kcs1Δ* mutants. Yeast strains used in (**A**, **B**) were grown to mid-log phase in SC-ino media, and sugar extraction was performed as described in Methods. BY4741 *opi1Δ* (yBS358) and W303 *opi1Δ* (yBS45) yeast were similarly analysed as positive controls for increased intracellular inositol levels. Graphs represent averages ± s.e.m (*n* ≥ 3 biological replicates, BY4741 wt vs. BY4741 *opi1Δ*: *P* = 0.003, W303 wt vs. W303 *opi1Δ*: *P* = 0.016, BY4741 *opi1Δ* vs. W303 *opi1Δ* *P* = 0.023, using two-tailed Student’s *t*-test). (**D**) Summary of LC-MS results of EV1C. (**E**) *opi1Δkcs1Δ* shows reduced growth rate but maintained viability compared to *opi1Δ*. W303 *opi1Δ* (yBS45) and *opi1Δkcs1Δ* (yBS186) were plated on SC-ino plates as described in Methods. Graphs represent averages ± s.e.m (*n* = 3 biological replicates, Day 1: *P* = 0.0038, using two-tailed Student’s t *t*-test). (**F**) Analysis of key genes controlling inositol metabolism revealed changes in *INM1* and *ITR1* levels in *opi1Δkcs1Δ* yeast. Strains and growth conditions were described in Fig. 1C. RNA extraction and RT-qPCR were performed as described in Methods. Graphs represent averages  ± s.e.m (*n* ≥ 3 biological replicates, *ITR1* expression in wt + I vs. *opi1Δ* + I: *P* = 0.042, wt + I vs. *opi1Δ kcs1Δ* + I: *P* = 0.01, *INM1* expression in *opi1Δ* -I vs. *opi1Δkcs1Δ* -I: P = 0.016, wt + I vs. *opi1Δ* + I: *P* = 0.02, *INM2* expression in wt + I vs. *opi1Δ kcs1Δ* + I: *P* = 0.042, using two-tailed Student’s *t*-test on the ln(2^−ΔCt^) values).

In budding yeast, intracellular inositol levels are determined by several interconnected pathways (Fig. 1A), endogenous synthesis followed by dephosphorylation by inositol monophosphatase, utilisation to synthesise lipid-bound phosphoinositides and cytosolic inositol phosphates, import via the Itr1 and Itr2 transporters, and export through an as-yet unidentified transporter. A recent study suggests that PP-InsPs synthesised by Kcs1, primarily 5-InsP_7_ (Fig. 1B), exert feedback regulation on basic inositol metabolism. Specifically, deletion of *KCS1* was reported to cause inositol auxotrophy via downregulating *INO1* expression (Ye et al, 2013). However, this observation conflicts with several radioactive labelling studies showing that the *kcs1Δ::KANMX* strain (BY4741) grows efficiently in inositol-free media supplemented with sub-limiting concentrations of [³H]-inositol (Laha et al, 2019; Seeds et al, 2005). This discrepancy may have arisen from the direct use of the *kcs1Δ::KANMX* strain from the yeast knockout collection (Winzeler et al, 1999), which was later reported to carry additional mutations by the O’Shea laboratory (Huang and O’Shea, 2005). Indeed, after backcrossing (Saiardi et al, 2002), we found that BY4741 *kcs1Δ::KANMX* grew in inositol-free media (Fig. EV1A). An independently generated *kcs1Δ::LEU2* mutant in the W303 background also grew in inositol-free media (Fig. EV1B). We next quantified intracellular inositol levels (Su et al, 2025) in wild-type and *kcs1Δ* yeast grown in inositol-free media. While the inositol-overproducing *opi1Δ* strains showed the expected increase in intracellular inositol levels in both genetic backgrounds, *kcs1Δ* strains had levels of inositol similar to their respective wild-type controls (Fig. EV1C,D), further confirming that inositol biosynthesis occurs in the absence of Kcs1.

It was noteworthy that inositol homoeostasis differed between the two strain backgrounds. Wild-type BY4741 cells had slightly lower intracellular inositol levels than wild-type W303 cells, whereas BY4741 *opi1Δ* strains accumulated more free inositol than their W303 *opi1Δ* counterparts (Fig. EV1C,D). This observation is consistent with previous reports highlighting metabolic differences between these strains (Miles et al, 2023; Ocampo et al, 2012). Given that W303 is more commonly used in recent years, we analysed this strain for most experiments in this study.

The reported downregulation of *INO1* expression in BY4741 *kcs1Δ* (Ye et al, 2013), which we independently confirmed in W303 *kcs1Δ* (see below), did not lead to a decrease in intracellular inositol levels (Fig. EV1C,D), suggesting that *KCS1* deletion dysregulates other aspects controlling inositol homoeostasis (Fig. 1A) to compensate for the reduction in endogenous synthesis. To elucidate this point, we examined Kcs1’s role in each pathway by coupling biochemical and gene expression analyses in both wild-type and *opi1Δ* background. First, we analysed Kcs1’s role in modulating the relative levels of endogenously synthesised and exogenously acquired inositol. In inositol-free conditions, the *opi1Δkcs1Δ* double mutant showed a further increase in intracellular inositol levels compared to the *opi1Δ* single mutant (Fig. 1C, -I, ^12^C), despite a reduction in *INO1* expression (Fig. 1D). We noticed a significant increase in the expression of inositol monophosphatase *INM1* in *opi1Δkcs1Δ* (Fig. EV1F), which could contribute to the observed increase in inositol levels. When exogenous ¹³C_6_-inositol was added, both wild-type and *kcs1Δ* strains repressed endogenous inositol synthesis from glucose (Fig. 1C, +I, ^12^C), and the levels of imported inositol were similar between the two strains (Fig. 1C, +I, ¹³C_6_). ¹²C- and ¹³C_6_-inositol were increased in *opi1Δ* cells as previously reported (Su et al, 2025) (Fig. 1C, +I), and saw further increases in *opi1Δkcs1Δ* cells (Fig. 1C, +I). We did not observe a statistically significant difference in transcript levels of genes controlling inositol metabolism in *opi1Δkcs1Δ* compared to *opi1Δ* in inositol-replete conditions (Fig. EV1F), suggesting that other pathways were affected in *opi1Δkcs1Δ* to further upregulate inositol levels.

We next investigated whether Kcs1 regulates the overall utilisation of inositol for the synthesis of cytosolic and water-soluble InsPs and membrane-bound PtdInsPs (Fig. 1A). We employed [³H]-inositol tracer and allowed it to be fully incorporated in the yeast PtdInsPs, InsPs, and PP-InsPs metabolome by labelling the yeast cultures overnight. InsPs and PP-InsPs were quantified by measuring radioactivity in the water-soluble fraction of yeast extracts (Fig. 1E). Membrane-bound PtdInsPs were extracted from the acid-insoluble pellet using organic solvents and quantified by radioactive counting (Fig. 1E). No significant difference was observed in either category of metabolites across all four strains, indicating that Kcs1 and Opi1 do not regulate the overall distribution of inositol between PtdInsPs, InsPs, and PP-InsPs (Fig. 1E). Consistently, we did not observe any change in gene expression levels of phosphoinositide synthase *PIS1* when cells grew in inositol-free media (Fig. EV1F).

Overall, our results suggest that Kcs1 does not regulate the utilisation of inositol, and it only affects the expression of genes involved in inositol import and the dephosphorylation step of endogenous synthesis in *opi1Δ* background. Hence, it remains unknown how *kcs1Δ* yeast maintains intracellular inositol levels comparable to wild-type, despite the downregulation of *INO1* expression under inositol-free conditions. This prompted us to explore the possibility that Kcs1 regulates the little-known inositol export pathway.

### Inositol export is reduced in *opi1Δkcs1Δ*

**Figure 2:**
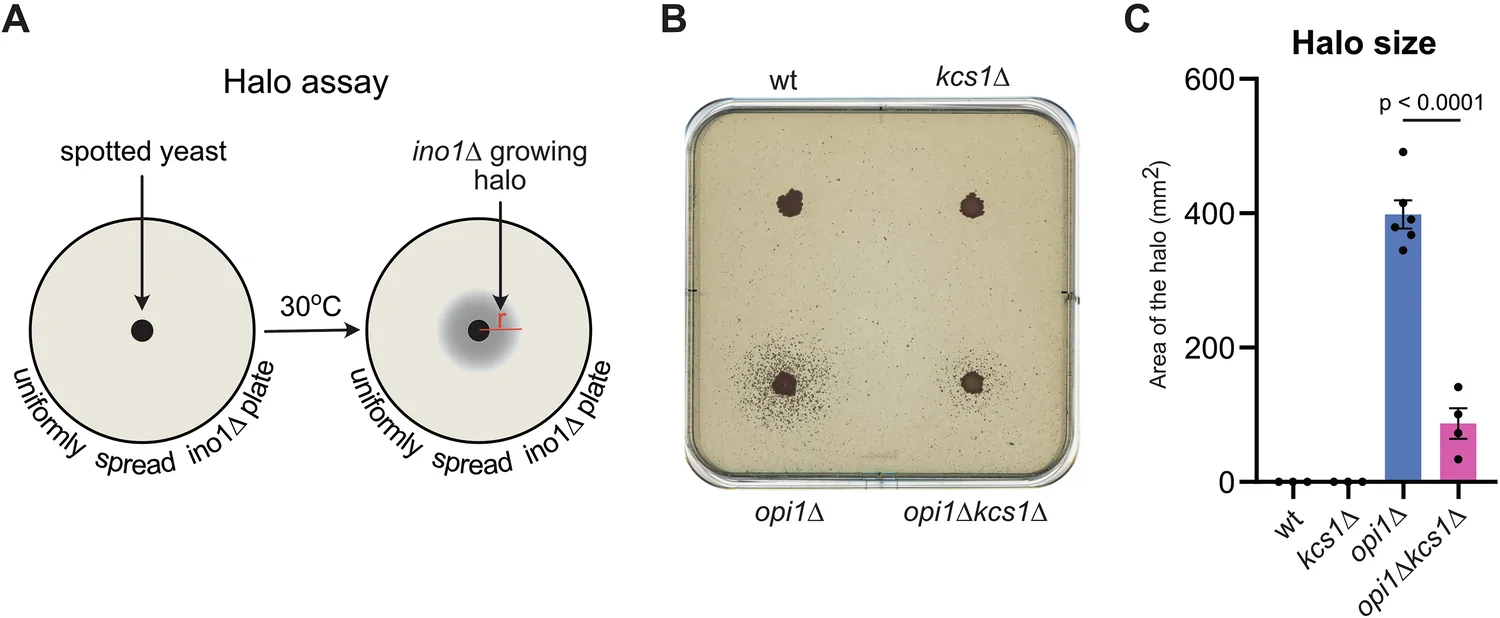
Inositol export is downregulated in *opi1Δkcs1Δ* yeast. (**A**) Diagram describing the inositol export assay. Briefly, *ino1Δ* yeast is spread evenly on an SC/SD -ino plate, and the test strains are spotted at the centre. Exported inositol supports the growth of an *ino1Δ* halo around the spotted strain. (**B**) Inositol export is reduced in the *opi1Δkcs1Δ* mutant. The halo assay was set up as described in Methods section. *ino1Δ* (yBS88) was spread on SC-ino solid media and WT (yBS1), *kcs1Δ* (yBS2), *opi1Δ* (yBS45) or *opi1Δkcs1Δ* (yBS186) yeasts were spotted at the centre. Shown is the representative image of three biological repeats. (**C**) Quantitative measurement of the *ino1Δ* halo. Graphs represent averages ± s.e.m (*n* ≥ 3 biological replicates, *P* < 0.0001, using two-tailed Student’s *t*-test). Source data are available online for this figure.

To investigate the role of Kcs1 in inositol export, we used the *opi1Δ* yeast model, in which elevated *INO1* expression triggers overproduction and export of inositol (Greenberg et al, 1982). We measured inositol export by the halo assay, by spreading inositol-auxotrophic *ino1Δ* strain evenly on an inositol-free plate and spotting the exporter strain at the centre (Greenberg et al, 1982). Inositol exported from the central yeast diffuses outward, and the neighbouring *ino1Δ* cells uptakes this essential nutrient, divides and forms a halo around the spotted yeast (Fig. 2A). The size of the halo is therefore determined by the amount of inositol exported. While a prominent *ino1Δ* halo was observed around *opi1Δ*, no visible halos were observed around either wild-type or *kcs1Δ* (Fig. 2B), because the latter strains do not produce sufficient inositol for export (Fig. EV1C,D). The halo around *opi1Δkcs1Δ* cells was significantly smaller than that around *opi1Δ* (Fig. 2B,C), suggesting that inositol export is likely impaired in *opi1Δkcs1Δ*.

The halo assay readout could be confounded by factors such as variations in the viability and growth rate of the spotted strains. To evaluate this, we performed a colony formation assay on SC-ino. We found that although *opi1Δkcs1Δ* colonies emerged more slowly than *opi1Δ*, they produced similar numbers of viable colonies on the day when halo assay results are normally analysed (EV1E). Thus, the slower growth rate of *opi1Δkcs1Δ* may have exaggerated the reduction in *ino1Δ* halo size. However, LC-MS analysis, which is free from the bias caused by yeast growth rate, demonstrates that the *opi1Δkcs1Δ* mutant has significantly higher levels of inositol than *opi1Δ* (Fig. 1C). Hence, we conclude that inositol export is impaired in *opi1Δkcs1Δ*, and we further supported this finding by analysing the *kcs1Δ* single mutant using a different assay later in the study.

### Inositol export is regulated by inositol pyrophosphates

**Figure 3:**
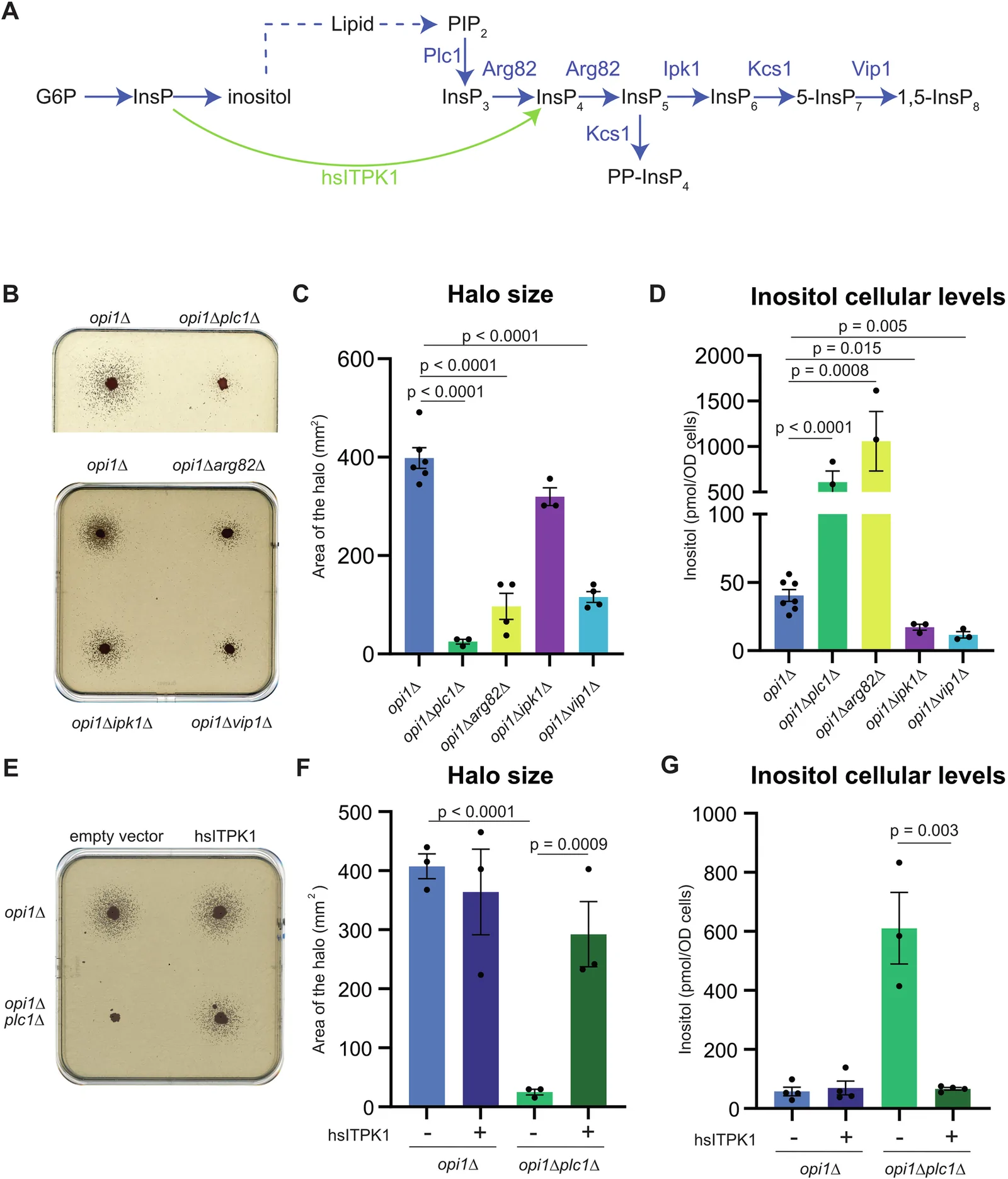
PP-InsP synthesis is required to promote inositol export in yeast. (**A**) Diagram describing the InsP and PP-InsP biosynthesis pathway. The conserved yeast pathways were shown in blue, and the human ITPK1 pathway, which bypasses the requirement of Plc1 to generate InsPs and PP-InsPs, was shown in green. (**B**) *ino1Δ* halo size was reduced on plates spread with *opi1Δplc1Δ*, *opi1Δarg82Δ* and *opi1Δvip1Δ*. *opi1Δ* (yBS45), *opi1Δ plc1Δ* (yBS111), *opi1Δarg82Δ* (yBS109), *opi1Δipk1Δ* (yBS187) and *opi1Δvip1Δ* (yBS189) were analysed by the halo assay. (**C**) Quantification of (**B**). Graphs represent averages ±  s.e.m (*n* ≥ 3 biological replicates, *opi1Δ* vs. *opi1Δplc1Δ*: *P* < 0.0001. *opi1Δ* vs. *opi1Δarg82Δ*: *P* < 0.0001. *opi1Δ* vs. *opi1Δvip1Δ*: *P* < 0.0001, using two-tailed Student’s *t*-test). (**D**) Intracellular levels of inositol were increased in *opi1Δplc1Δ* and *opi1Δarg82Δ*, whereas they were decreased in *opi1Δipk1Δ* and *opi1Δvip1Δ*. Yeast strains from (**B**) were grown to mid-log phase in SC-ino, and sugar extracts were analysed by LC-MS. Graphs represent averages ± s.e.m (*n* ≥ 3 biological replicates, *opi1Δ* vs. *opi1Δplc1Δ*: *P* < 0.0001. *opi1Δ* vs. *opi1Δarg82Δ*: *P* = 0.0008. *opi1Δ* vs. *opi1Δipk1Δ*: *P* = 0.015*. opi1Δ* vs. *opi1Δvip1Δ*: *P* = 0.005, using two-tailed Student’s *t*-test). (**E**) Human ITPK1 restored inositol export in *opi1Δplc1Δ* yeast. *ino1Δ* transformed with an empty *URA3* vector (pCA45) was spread on SD-ura-ino. Exporter strains examined were: *opi1Δ* or *opi1Δplc1Δ* yeast strains transformed with an empty *URA3* vector (yBS176 and yBS178, respectively) or hsITPK1(yBS180 and yBS182, respectively). (**F**) Quantification of (**E**). Graphs represent averages ±  s.e.m (*n* = 3 biological replicates, *opi1Δ* + vector vs. *opi1Δplc1Δ* + vector: *P* < 0.0001, *opi1Δplc1Δ* +  vector vs. *opi1Δplc1Δ* + hsITPK1, *P* = 0.0009, using two-tailed Student’s *t*-test). (**G**) Human ITPK1 rescued the inositol accumulation phenotype in *opi1Δplc1Δ* yeast. Strains used in (**E**) were harvested in mid-log phase in SD-ura-ino media. Sugar extracts were analysed by LC-MS. Graphs represent averages  ± s.e.m (*n* ≥ 3 biological replicates, *opi1Δplc1Δ* +  vector vs. *opi1Δplc1Δ* + hsITPK1: *P* = 0.003, using two-tailed Student’s *t*-test). Source data are available online for this figure.

In budding yeast, InsPs and PP-InsPs are synthesised through the sequential actions of the inositol kinases Arg82, Ipk1, Kcs1 and Vip1, starting from InsP_3_, which is generated by the activity of phospholipase C (Plc1) on lipid-bound PIns(4,5)P_2_ (Fig. 3A). To investigate if it is the Kcs1 protein, or a specific alteration of InsPs and/or PP-InsPs metabolism that regulates inositol export, we screened all the mutants affecting the pathway.

**Figure EV2:**
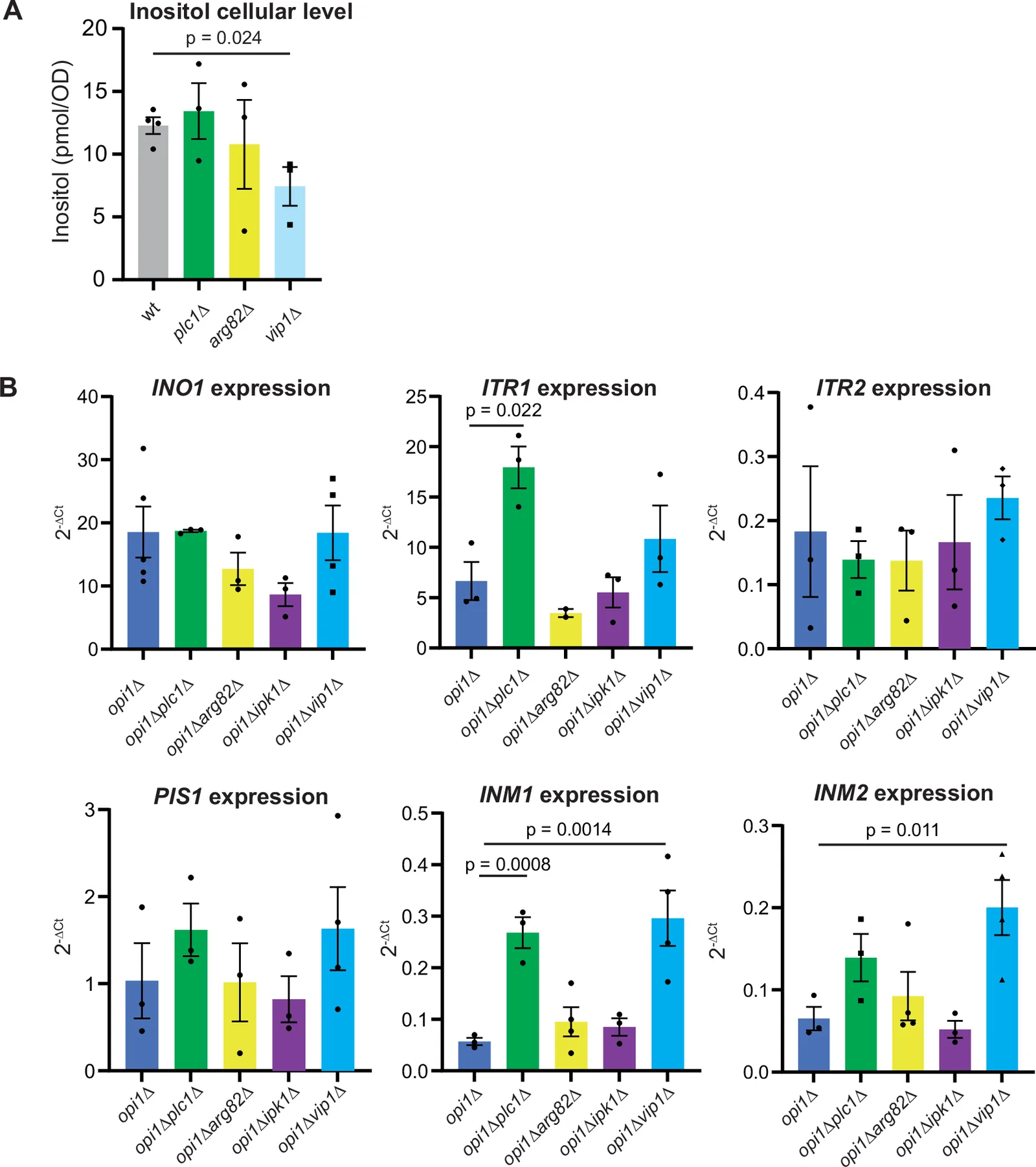
Further analysis of mutants in the inositol-phosphate metabolic pathway. (**A**) Intracellular levels of inositol remain unchanged in *plc1Δ* or *arg82Δ* and was reduced in *vip1Δ* cells. Wild type (yBS1), *plc1Δ* (yBS81) or *arg82Δ* (yBS92) cells were grown to mid-log phase in SC-ino media and sugar extracts were analysed by LC-MS. Graphs represent averages ± s.e.m (*n* ≥ 3 biological replicates, wt vs. *vip1Δ*: *P* = 0.024, using two-tailed Student’s *t*-test). (**B**) Evaluation of mRNA levels of key genes controlling inositol metabolism revealed changes in *opi1Δplc1Δ* and *opi1Δvip1Δ* yeast. Yeast strains used in Fig. 3B were grown to mid-log phase in SC-ino media. RNA extraction and RT-qPCR were performed as described in Methods. Graphs represent averages ± s.e.m (*n* ≥ 3 biological replicates, *ITR1* expression in *opi1Δ* vs. *opi1Δplc1Δ*: *P* = 0.022, *INM1* expression in *opi1Δ* vs. *opi1Δplc1Δ*: *P* = 0.0008, *INM1* expression in *opi1Δ* vs. *opi1Δvip1Δ*: *P* = 0.0014, *INM2* expression in *opi1Δ* vs. *opi1Δvip1Δ*: *P* = 0.011, using two-tailed Student’s *t*-test on the ln(2^−ΔCt^) values).

We first observed that deletion of *PLC1* in the *opi1*Δ background caused a significant reduction in the *ino1Δ* halo size, accompanied by a drastic increase in intracellular inositol levels (Fig. 3B–D), indicating reduced inositol export. This effect was *OPI1*-dependent, as the *plc1Δ* single mutant did not show significant changes in intracellular inositol levels (Fig. EV2A). In addition to its essential role in generating InsPs and consequently PP-InsPs in budding yeast, Plc1 also regulates lipid metabolism by producing diacylglycerol (DAG), a substrate for phosphatidic acid synthesis (Macrae et al, 2025). To understand which function of Plc1 regulates inositol export, we expressed the human ITPK1, which bypasses the requirement for Plc1 to generate InsPs and thus PP-InsPs in yeast (Fig. 3A) (Desfougeres et al, 2019). Heterologous expression of ITPK1 restored both intracellular inositol levels and *ino1Δ* halo size in the *opi1Δplc1Δ* mutant (Fig. 3E–G), further supporting a role for InsPs/PP-InsPs synthesis in regulating inositol export.

We next examined the individual contributions of specific InsPs-kinases towards inositol export. Arg82 carries out the sequential phosphorylation of InsP_3_ and InsP_4_ (Fig. 3A). Like *opi1Δplc1Δ* and *opi1Δkcs1Δ*, *opi1Δarg82Δ* reduced the size of the *ino1Δ* halo (Fig. 3B,C) and increased intracellular inositol levels (Fig. 3D). The *arg82Δ* single mutant did not change intracellular levels of inositol (Fig. EV2A), suggesting its impact on inositol export is *OPI1*-dependent. Further, *opi1Δarg82Δ* did not significantly change the expression of *INO1* or other genes associated with inositol metabolism compared to *opi1Δ* (Fig. EV2B). This indicates the mutant has a bona fide defect in inositol export. Therefore, blocking the synthesis of InsP/PP-InsP beyond InsP_3_ is detrimental to inositol export.

Ipk1 is an enzyme that converts InsP_5_ to InsP_6_, and the accumulated InsP_5_ in *ipk1Δ* could be phosphorylated by Kcs1 to form PP-InsP_4_ (Saiardi et al, 2002; York et al, 1999). In contrast to *opi1Δplc1Δ*, *opi1Δarg82Δ* and *opi1Δkcs1Δ*, *opi1Δipk1Δ* showed a similarly-sized halo as *opi1Δ*, and even reduced intracellular inositol levels (Fig. 3B–D). Therefore, blocking the synthesis of InsP_6_ does not affect inositol export. Instead, PP-InsP_4_ functionally substitutes for InsP_7_ in regulating inositol export, like it does for vesicular trafficking (Saiardi et al, 2005), polyP production (Lonetti et al, 2011) and bioenergetic metabolism (Szijgyarto et al, 2011).

Vip1 works downstream of Kcs1 and primarily phosphorylates 5-InsP_7_ to form 1,5-InsP_8_ (Fig. 3A). In contrast to all other mutants evaluated, *opi1Δvip1Δ* simultaneously reduced intracellular inositol levels and *ino1Δ* growth halo (Fig. 3B–D). This suggests that inositol synthesis is also reduced in the mutant, thus confounding the analysis on export. Notably, *vip1Δ* regulates inositol synthesis independently of *OPI1*, as the *vip1Δ* single mutant also reduced inositol levels compared to wild-type yeast (Fig. EV2A). Further, the effect on inositol synthesis is not attributed to gene expression changes, as *INO1* expression was intact (Fig. EV2B) and *INM1* and *INM2* expression were paradoxically upregulated (Fig. EV2B). Therefore, Vip1 regulates inositol synthesis through an unknown mechanism, and we established its role in inositol export by a different approach later in the study.

### Inositol exporter identified as Pho84

**Figure 4:**
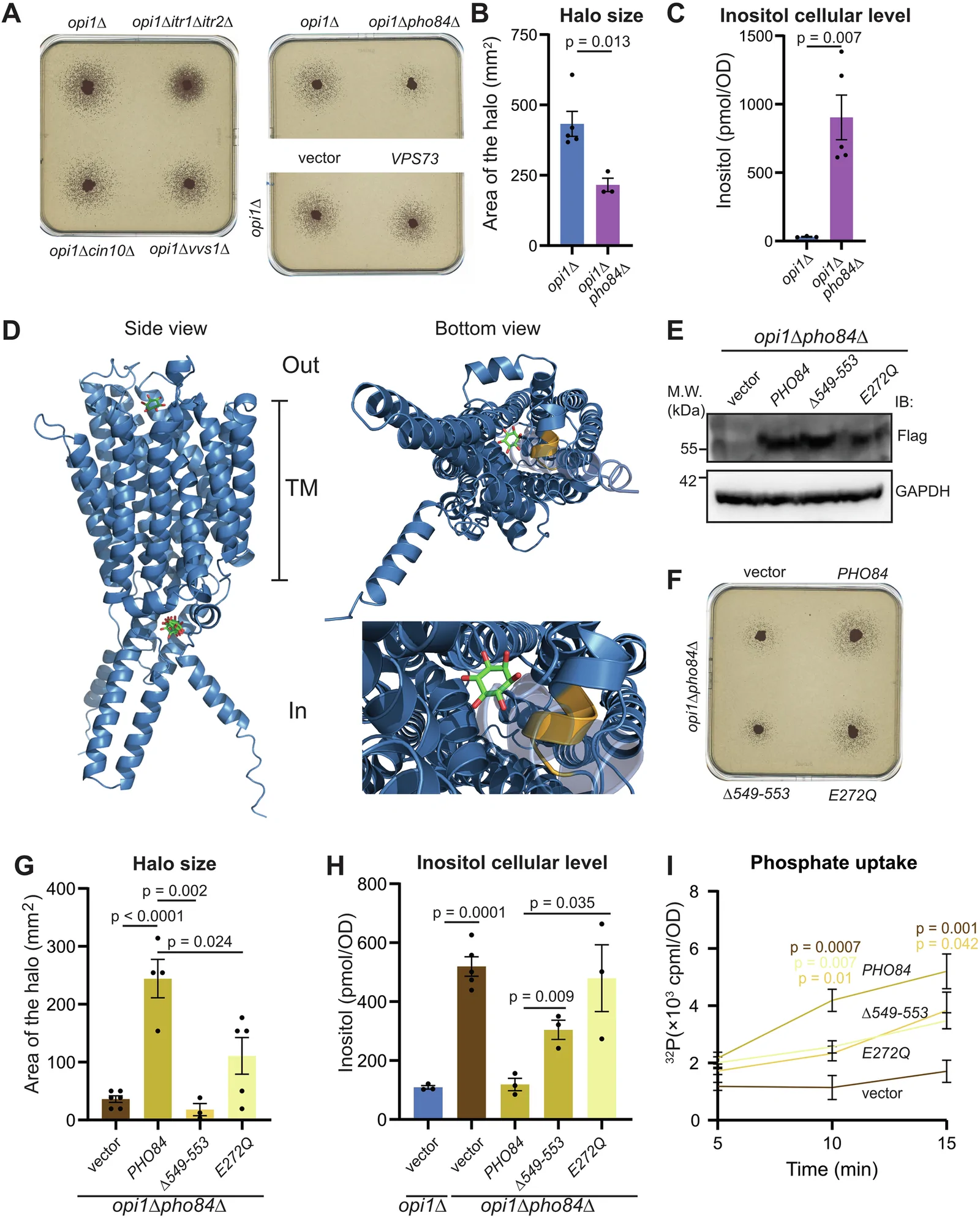
Pho84 acts as an inositol exporter in yeast. (**A**) Pho84 regulates inositol export from *opi1Δ*, whereas Itr1, Itr2, Cin10, Vvs1 and Vps73 do not. Inositol export from *opi1Δ* (yBS45), *opi1Δ itr1Δ itr2Δ* (yBS174), *opi1Δ cin10Δ* (yBS78), *opi1Δ vvs1Δ* (yBS286) and *opi1Δ pho84Δ* (yBS148) yeast strains were analysed by halo assay on SC -ino. *opi1Δ* transformed with empty *CEN* plasmid (yBS359) or pADH-*VPS73* (yBS368) was analysed by halo assay on SC -ura -ino. (**B**) *opi1Δ pho84Δ* significantly reduced *ino1Δ* halo size. Graphs represent averages  ± s.e.m (*n* ≥ 3 biological replicates. *P* = 0.013, using two-tailed Student’s *t*-test). (**C**) *opi1Δ pho84Δ* accumulates high levels of intracellular inositol. Strains used in (**B**) were harvested in log phase in SC-ino media. Graphs represent averages ± s.e.m (*n* ≥ 3 biological replicates, *P* = 0.007, using two-tailed Student’s *t*-test). (**D**) Predicted inositol binding sites on Pho84. Structural modelling was generated using DiffDock-L (https://neurosnap.ai). The side view image shows that four out of five top-predicted binding sites interact with a region within the cytoplasmic domain (In) and one interacts with the extracellular domain (Out). The top predicted inositol docking site is highlighted in the bottom view, and amino acids 549-553 of Pho84 (highlighted in bronze) is within close proximity (an enlarged view is shown at the bottom). (**E**) Western blotting demonstrating the expression of plasmid-borne Pho84-Flag proteins. *opi1Δpho84Δ* cells transformed with vector (yBS362), prom-*PHO84*-Flag (yBS363), prom-*pho84*-*Δ549-553*-Flag (yBS382) and prom-*pho84*-*E272Q*-Flag (yBS375) were grown in 0.2 mM phosphate SC -ura -ino media for 3 h to induce Pho84 expression. Protein extracts were prepared as described in Methods, and Pho84 protein expression was detected using anti-Flag immunoblotting. GAPDH was used as a loading control. (**F**) Pho84 sugar-binding mutants show reduced ability to export inositol. Strains from (**E**) were analysed by the halo assay on SC -ura -ino. (**G**) Quantification of (**F**). Graphs represent averages ± s.e.m (*n* ≥ 3 biological replicates, vector vs. *PHO84*: *P* < 0.0001, *PHO84* vs. *pho84*-*Δ549-553*: *P* = 0.002, *PHO84* vs. *pho84*-*E272Q*: *P* = 0.024, using two-tailed Student’s *t*-test). (**H**) Inositol is accumulated inside *opi1Δpho84Δ*, and the accumulation is reversed by the presence of *PHO84*, but not by *pho84*-*Δ549-553* or *pho84-E272Q*. Strains from (**E**) were grown in SC -ura -ino and sugar extracts were analysed by LC-MS. Graphs represent averages ± s.e.m (*n* ≥ 3 biological replicates, *opi1Δ* + vector vs. *opi1Δpho84Δ* + vector: *P* = 0.0001, *opi1Δpho84Δ* +  *PHO84* vs. *opi1Δpho84Δ * + *pho84*-*Δ549-553*: *P* = 0.009, *opi1Δpho84Δ* +  *PHO84* vs. *opi1Δpho84Δ* + *pho84*-*E272Q*: *P* = 0.035, using two-tailed Student’s *t*-test). (**I**) Inositol export mutants of *PHO84* also show reduced ability to import phosphate. *pho84Δ* cells transformed with vector (yBS393), prom-*PHO84*-Flag (yBS392), prom-*pho84*-*Δ549-553*-Flag (yBS398) and prom-*pho84*-*E272Q*-Flag (yBS396) were analysed for their ability to take up ^32^Pi, as described in Methods. Graphs represent averages ± s.e.m (*n* = 4 biological replicates, using two-tailed Student’s *t*-test comparing to the corresponding time points of +*PHO84*, vector 10 min: *P* = 0.0007, *Δ549-553* 10 min: P = 0.007, *E272Q* 10 min: *P* = 0.01; vector 15 min: *P* = 0.001, *E272Q* 15 min: *P* = 0.042). Source data are available online for this figure.

Although it has been known for decades that *opi1*Δ yeast exports inositol (Greenberg et al, 1982), little is known about the exporters carrying out the process. We first tested the possibility that the inositol importers Itr1 and Itr2 act in reverse in the *opi1Δ* background, in which intracellular inositol levels are increased. The size of the *ino1Δ* halo around the *opi1Δitr1Δitr2Δ* triple mutant did not change compared to *opi1Δ* (Fig. 4A). Instead, the density of *ino1*Δ colonies around *opi1Δitr1Δitr2Δ* (Fig. 4A) was higher, because the triple mutant is unable to reimport exported inositol, thus better supporting the growth of the surrounding *ino1*Δ cells.

**Figure EV3:**
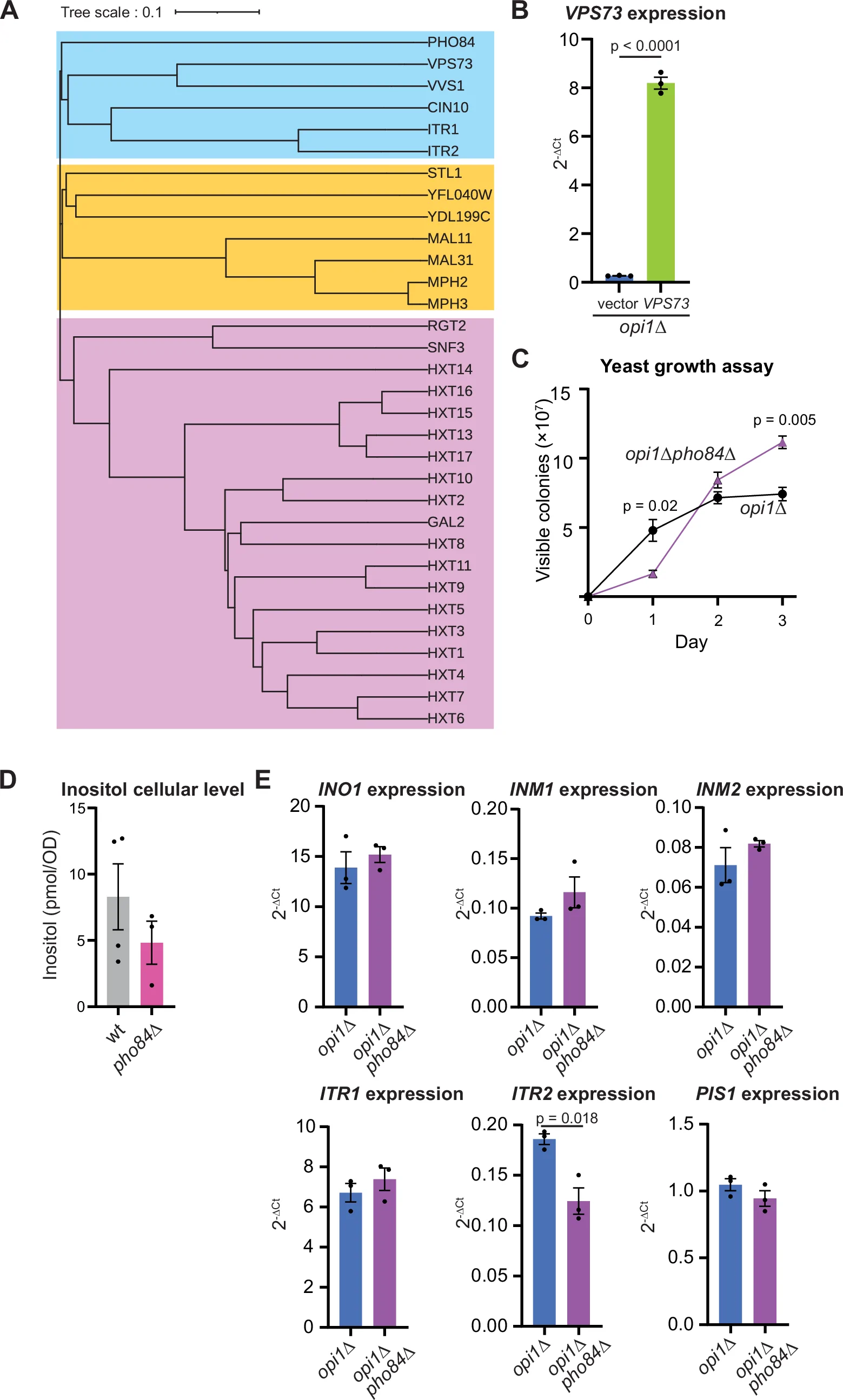

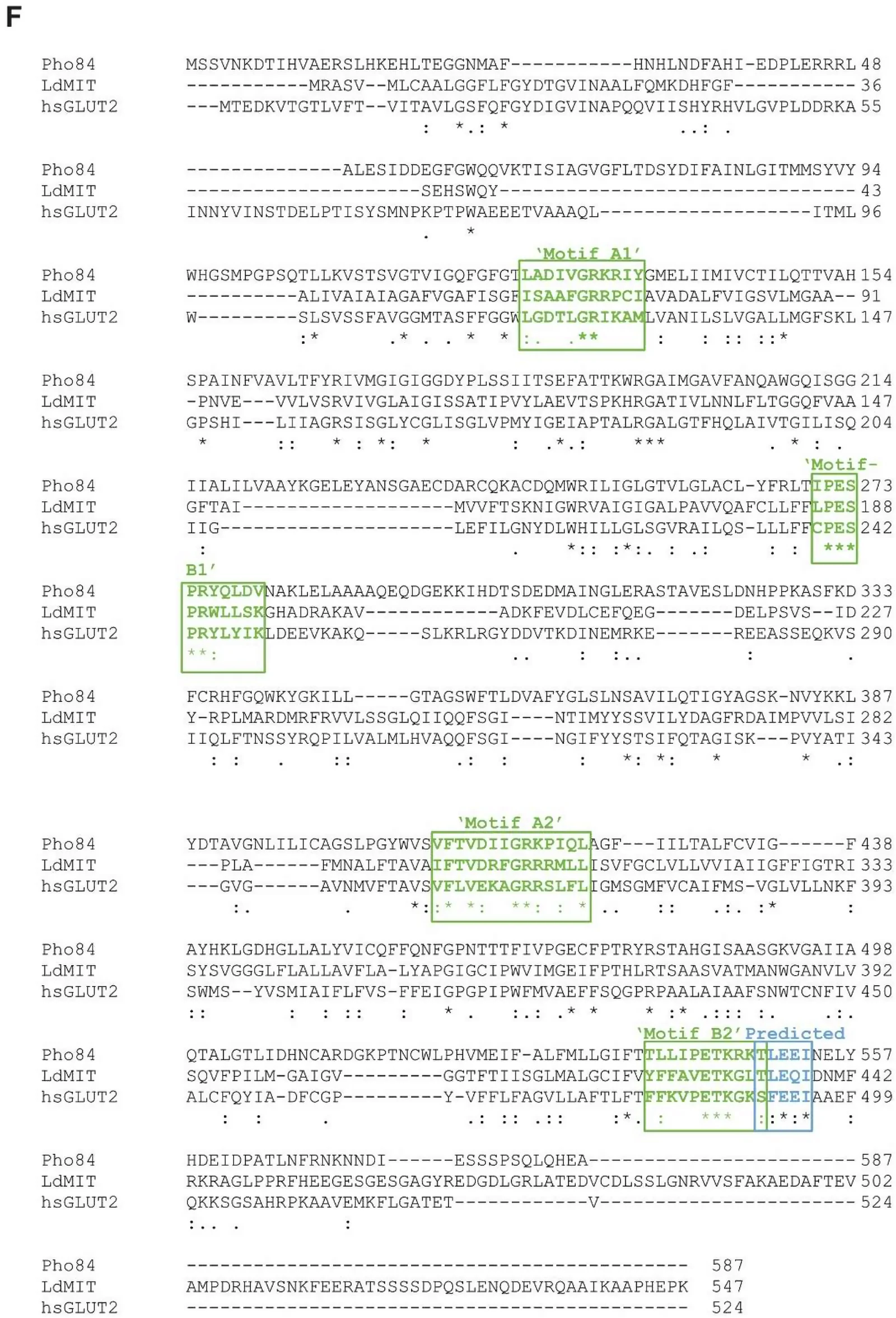
Sequence homology and gene expression analysis of inositol exporters in yeast. (**A**) Phylogenetic tree of the yeast sugar transporters. Transporters with the highest sequence homology to the inositol importers Itr1 and Itr2 were highlighted in a blue box. Amino acid sequences of sugar transporters were extracted from the *Saccharomyces* genome database, and the phylogenetic tree was generated using Clustal Omega. https://www.ebi.ac.uk/jdispatcher/msa/clustalo. (**B**) RT-qPCR confirmed overexpression of *VPS73* under the ADH promoter. RNA was extracted from strains used in Fig. 4A and analysed as described in Methods. Graphs represent averages ± s.e.m (*n* = 3 biological replicates, *P* < 0.0001, using two-tailed Student’s *t*-test on the ln(2^-ΔCt^) values). (**C**) *opi1Δpho84Δ* shows slow growth initially but ultimately has higher viability than *opi1Δ*. Yeast growth assay was performed as described in Methods. Graphs represent averages ± s.e.m (*n* = 3 biological replicates, Day 1: *P* = 0.02, Day 3: *P* = 0.005, using two-tailed Student’s *t*-test). (**D**) Intracellular inositol levels were unchanged in *pho84Δ* single mutant. Wild type (yBS1) and *pho84Δ* (yNF1) yeast cells were grown in SC -ino and sugar extracts were analysed by LC-MS. Graphs represent averages ± s.e.m. (*n* ≥ 3 biological replicates, not significant, using two-tailed Student’s *t*-test). (**E**) Expression of genes regulating inositol homoeostasis is unchanged in *opi1Δpho84Δ* yeast, except for *ITR2*. Graphs represent averages ± s.e.m (*n* = 3 biological replicates, *ITR2* expression in *opi1Δ* vs. *opi1Δpho84Δ*: *P* = 0.018, using two-tailed Student’s *t*-test on the ln(2^−ΔCt^) values). (**F**) Alignment of Pho84, LdMIT and hsGLUT2 showed sequence homology in the inositol binding motives. The alignment was generated using multi sequence alignment (Clustal Omega). Highlighted in green are the inositol-binding motives identified in LdMIT. Highlighted in blue is the region predicted to interact with inositol based on structural modelling.

Itr1 and Itr2 belong to a large family of facilitated hexose-transporters (Ozcan and Johnston, 1999), leading to the hypothesis that the inositol exporter(s) might show sequence homology to these importers. Generation of the phylogenetic tree of all 34 yeast hexose transporters revealed that Cin10, Vps73, Vvs1 and Pho84 branch together with Itr1 and Itr2 (Fig. EV3A). We thus generated mutants of these genes in the *opi1Δ* background. *opi1Δvvs1Δ* and *opi1Δcin10Δ* did not alter inositol export (Fig. 4A). We attempted to generate the *opi1Δvps73Δ* mutant, but it was inviable, and therefore resorted to *VPS73* overexpression, which did not affect inositol export (Figs. 4A and EV3B). Surprisingly, deletion of *PHO84*, a long-established phosphate transporter, significantly reduced *ino1*Δ halo size (Fig. 4A,B). Colony formation assay showed that although *opi1Δpho84Δ* colonies emerged slowly compared to *opi1Δ* on day 1, they showed a ~50% increase in colony numbers compared to *opi1Δ* on day 3 (Fig. EV3C), when the halo assay is normally imaged. Hence, the halo assay may have underestimated the export defect of *opi1Δpho84Δ*, because increased number of cells were present to produce and export inositol. Indeed, when we measured intracellular inositol levels, *opi1Δpho84Δ* showed a drastic accumulation of inositol inside the cells (Fig. 4C). The increased concentration of inositol was *OPI1*-dependent, as the *pho84Δ* single mutant did not change inositol concentration (Fig. EV3D). Complementarily, RT-PCR analysis indicated that, except for a reduction in *ITR2* levels, which could not explain the accumulation of inositol inside the cells, expression of other genes involved in inositol metabolism did not change in *opi1Δpho84Δ* (Fig. EV3E), further upholding the idea that Pho84 is directly involved in inositol export.

**Figure EV4:**
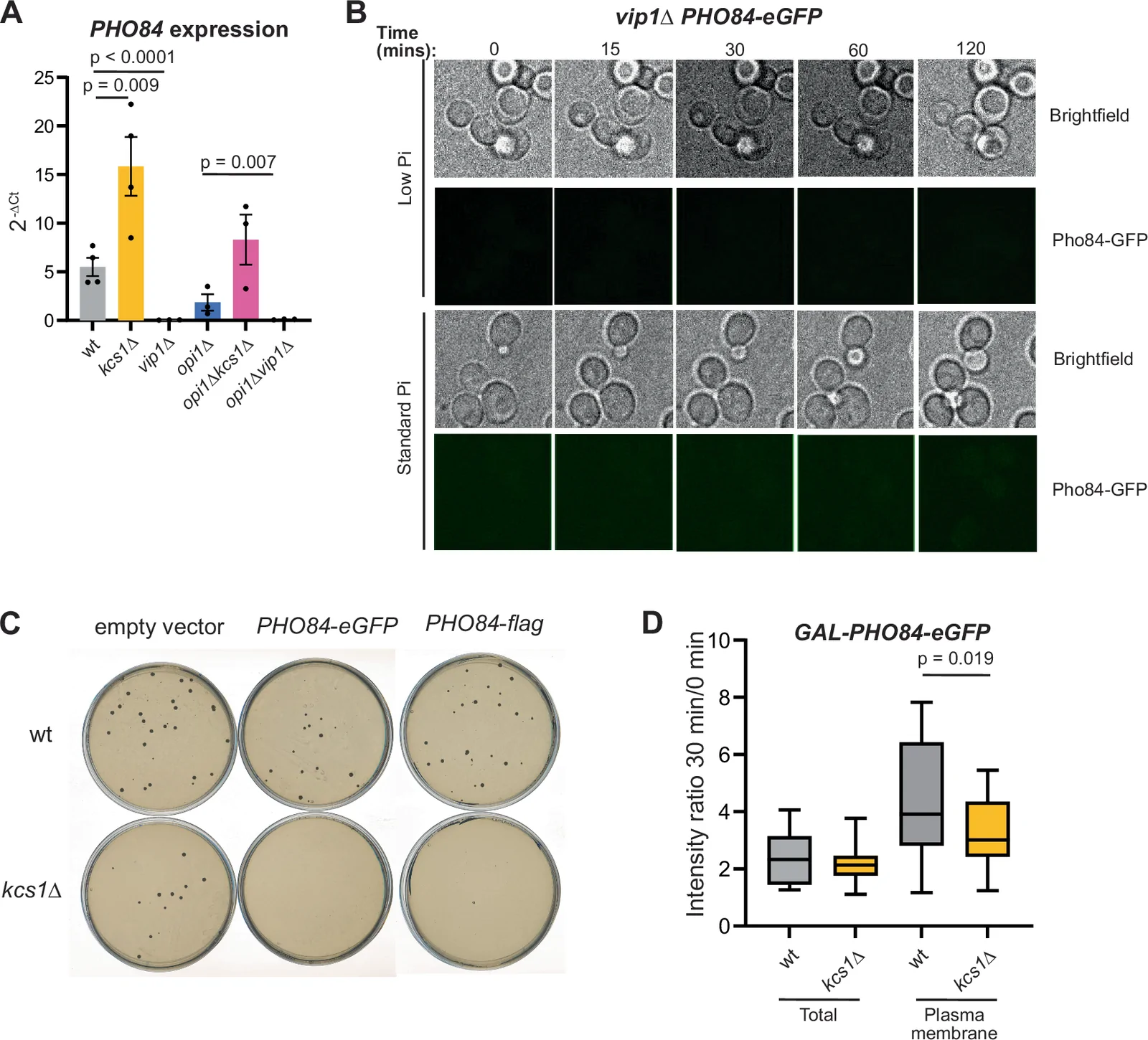
Pho84 is regulated by Vip1 and Kcs1 in distinct manners. (**A**) *PHO84* mRNA levels were upregulated in *kcs1Δ* (yBS2) and *opi1Δkcs1Δ* (yBS186), whereas they were downregulated in *vip1Δ* (yBS67) and *opi1Δvip1Δ* (yBS189). RNA was extracted from log phase cells grown in SC-ino media, and RT-qPCR was performed as described in Fig. 1D. Graphs represent averages ± s.e.m (*n* ≥ 3 biological replicates, wt vs. *kcs1Δ*: *P* = 0.009, wt vs. *vip1Δ*: *P* < 0.0001, *opi1Δ* vs. *opi1Δvip1Δ*: *P* = 0.007, using two-tailed Student’s *t*-test comparing ln(2^−ΔCt^) values). (**B**) Pho84-eGFP is undetectable in BY4741 *vip1Δ* cells (yNF10). Live imaging was performed similarly to Fig. 5C. (**C**) Additional copies of Pho84 cause lethality in *kcs1Δ* yeast. Empty vector (pRS316), *CEN-prom-PHO84-eGFP* (pBS122) and *CEN-prom-PHO84-flag* (pBS114) plasmids were transformed into BY4741 wild type (yXZ3) or *kcs1Δ* (yXZ4) and plated on SD-ura. (**D**) Gal-Pho84-eGFP signal around the plasma membrane was reduced in *kcs1Δ* yeast. Wild type and *kcs1Δ* cells transformed with pRS426-*pGAL-PHO84-eGFP* (pBS423 and pBS426) were imaged 0 and 30 min after adding 2% galactose to induce the expression of Pho84-eGFP. GFP signal intensity in the whole cell and around the cell periphery (an approximation to the plasma membrane) were measured by ImageJ, corrected for background signal and the ratio of intensity at 0 and 30 min were calculated. ~25 cells from two biological repeats were analysed and presented as a box and whisker plot. From top to the bottom of each plot: max, upper quartile, median, lower quartile, min. *P* = 0.019, using two-tailed Student’s *t*-test.

Pho84 was first cloned in the early ‘90 by complementation screening following the constitutive synthesis of repressible acid phosphatase in a *pho84-mutant* strain (Bun-Ya et al, 1991). Although it belongs to the sugar transporter family, its ability to transport sugar was never studied, as later works solely focused on its role as a phosphate starvation-induced inorganic phosphate (Pi) transporter (Tomar and Sinha, 2014; Wykoff et al, 2007). Our finding that *PHO84* deletion downregulates inositol export (Fig. 4A–C) led us to visit the idea that Pho84 might play a role in transporting sugar, specifically inositol. To identify potential inositol binding sites, we first applied AlphaFold-based structural modelling and ligand docking using DiffDock-L (https://neurosnap.ai). Among the top five predicted inositol binding sites, one interacts with the extracellular domain, whereas the other four are positioned on the cytosolic side near the channel entry formed by the transmembrane helices (Fig. 4D, side view). A closer examination of the docking position with the highest confidence score revealed a proximity to a stretch of amino acid 549-TIEEL-553 (Fig. 4D, bottom view). To verify the importance of this region to inositol export, we generated the *pho84*-Δ(549-553) mutant. The mutant form was expressed at a similar level as the wild-type Pho84 (Fig. 4E) but failed to rescue the inositol export defect of *opi1*Δ*pho84*Δ (Fig. 4F–H), supporting the idea that Pho84 directly transports inositol. We next compared Pho84 with *Leishmania donovani* inositol transporter (LdMIT). Four inositol-binding domains were established in LdMIT (Seyfang and Landfear, 2000), all of which are conserved in Pho84 (Fig. EV3F). Interestingly, one of these domains (motif B2) overlaps with the Pho84 549-TIEEL-553 region identified by our molecular docking analysis. We mutated a different inositol binding motif (B1) in Pho84, whose mutation reduced inositol binding in LdMIT. Changing the conserved glutamic acid 272 residue to glutamine (Figs. EV3F and 4E) partially but significantly reduced Pho84’s ability to export inositol (Fig. 4F–H). These analyses thus support a model in which Pho84 directly interacts with inositol on its cytoplasmic side and transports it outside the cell. We further assessed whether Pho84’s ability to export inositol is linked to its role in taking up phosphate under phosphate depletion. Both inositol-exporting mutants of *PHO84* partially reduced the rate of ^32^P uptake (Fig. 4I). Since neither E272 nor 549-TIEEL-553 is located within the channel previously identified to bind and transport phosphate (Samyn et al, 2012), Pho84 might act as a phosphate-inositol antiporter, or coordinate their opposite transport via an indirect mechanism worthy of future exploration.

### Pho84 mediates a new crosstalk pathway balancing phosphate and inositol homoeostasis

**Figure 5:**
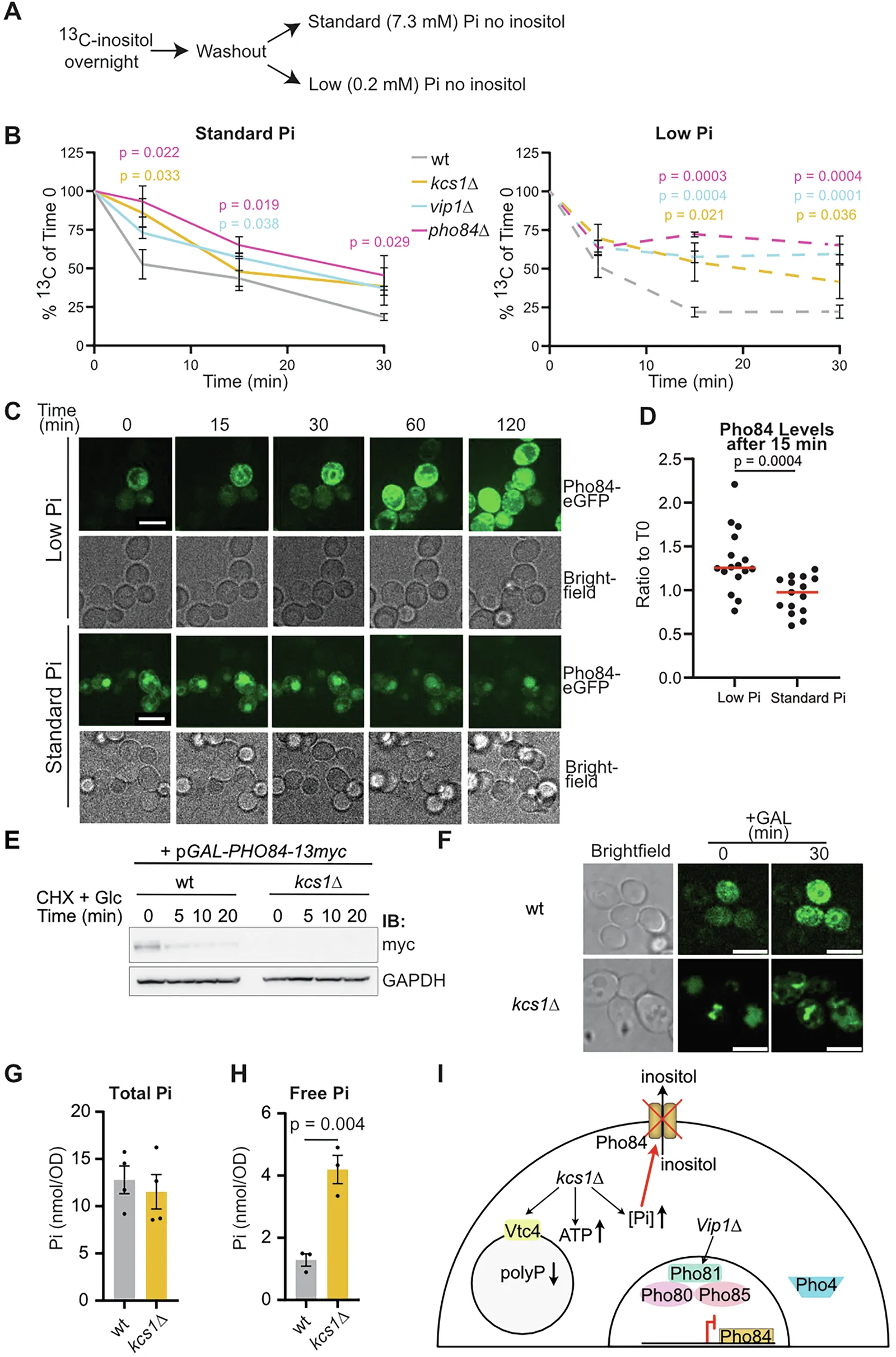
Inositol export is regulated by cellular phosphate and inositol pyrophosphate metabolism. (**A**) Schematics describing the yeast inositol export assay. Briefly, cells were labelled overnight with 50 µM ^13^C_6_-inositol. They were subsequently switched to growing in inositol-free media in standard or low Pi. (**B**) Pho84 contributes to inositol export in standard Pi and is the main exporter in low Pi. Wild type (yBS1), *pho84Δ* (yNF1), *kcs1Δ* (yBS2) and *vip1Δ* (yBS67) strains were analysed as described in (**A**). Cells were harvested 5, 15 and 30 min after switching to inositol-free media, and sugar extracts were analysed by LC-MS. Data were presented as percentage of time 0 value in the same experiment ± s.e.m (*n* ≥ 3 biological replicates. High Pi- comparing to wt matching time points, *pho84Δ* 5 min: *P* = 0.022, *kcs1Δ* 5 min: *P* = 0.033, *pho84Δ* 15 min: *P* = 0.019, *vip1Δ* 15 min: *P* = 0.038, *pho84Δ* 30 min: *P* = 0.029. Low Pi- comparing to wt at the corresponding time points, *pho84Δ* 15 min: *P* = 0.0003, *kcs1Δ* 15 min: *P* = 0.021, *vip1Δ* 15 min: *P* = 0.0004, *pho84Δ* 30 min: *P* = 0.0004, *kcs1Δ* 30 min: *P* = 0.036, *vip1Δ* 30 min: *P* = 0.0001. Statistics were performed using one-tailed Student’s *t*-test and were presented in the same colours as the strains. (**C**) Pho84 proteins are expressed at basal levels on the plasma membrane of some cells in standard Pi, and the levels were elevated by low Pi. BY *PHO84*-eGFP yeast (yNF4) was grown similarly as described in (**A**) and was analysed by live cell imaging. Scale bar = 5 µm. Shown are the representative cells of two independent imaging experiments. (**D**) Pho84 levels are increased in low Pi as early as 15 min after media switch, while remaining similar in standard Pi. Pho84-eGFP signals of Fig. 5C at the cell periphery were quantified, and the intensity at 15 min was compared to that of 0 min in the same cell. Red lines indicate median values (*n* = 15 from 2 biological repeats, *P* = 0.0004, using Mann–Whitney test). (**E**) Pho84 protein levels are reduced in *kcs1Δ*. Plasmid-borne p*GAL*-*PHO84-13MYC* was transformed into W303 wild-type (yBS421) and *kcs1Δ* (yBS422). The transformants were cultured in SC -ura + 2% raffinose + 0.02% glucose overnight, and *PHO84-13MYC* was induced for 30 min by the addition of 2% galactose. Transcription and translation were quenched by the addition of 2% glucose and 0.25 mg/mL cycloheximide. Pho84-13myc and GAPDH (loading control) were detected by anti-myc and anti-GAPDH immunoblotting. Shown is the representative of two biological repeats. (**F**) Galactose-induced expression of Pho84-GFP is present but mislocalised in *kcs1Δ*. Plasmid-borne p*GAL*-*PHO84-eGFP* was transformed into BY4741 wild-type (yBS423) and *kcs1Δ* (yBS426). Images were taken 30 min after galactose-induced expression. (**G**, **H**) Free Pi, not total Pi levels, change in *kcs1Δ*. Wild type (yBS1) and *kcs1Δ* (yBS2) were grown in the pre-washout condition of (**A**), and total Pi or free Pi levels were measured as described in Methods (*n* ≥ 3 biological replicates, free Pi: *P* = 0.004, using two-tailed Student’s *t*-test). (**I**) Model describing the roles of Kcs1 and Vip1 kinases in coordinating inositol and phosphate homoeostasis. Source data are available online for this figure.

The newly found function of Pho84 in exporting inositol prompted us to investigate a potential crosstalk connecting phosphate, inositol pyrophosphate and inositol metabolism. To measure the rate of inositol export without the confounding effect of endogenous synthesis, we established an in vivo assay that measures the export of exogenously added ^13^C_6_-inositol. Briefly, cells were incubated overnight with 50 μM ^13^C_6_-inositol, a concentration of exogenous inositol that represses endogenous inositol synthesis. We subsequently switched the yeast to inositol-free media containing standard (7.3 mM) or low (0.2 mM) phosphate (Fig. 5A). The resulting concentration gradient of inositol stimulates the export of intracellular ^13^C_6_-inositol. The high glucose content in yeast growth media saturates the LC-MS column, which prohibits accurate measurement of extracellular inositol. We therefore assessed inositol export by measuring the reduction of intracellular ^13^C_6_-inositol. In wild type cells, ^13^C_6_-inositol levels reduced to ~25% after 30 min in standard phosphate conditions (Fig. 5B, standard Pi). The rate of export was accelerated in low phosphate media, where ^13^C_6_-inositol levels dropped to 25% after 15 min (Fig. 5B, low Pi). The absence of a further ^13^C_6_-inositol reduction indicates that the exported inositol and its re-entry had reached equilibrium. *PHO84* deletion slowed down the rate of inositol export by ~50% in standard phosphate media (Fig. 5B, standard Pi) and stopped exporting inositol after 5 min in low phosphate media (Fig. 5B, low Pi). These results suggest that inositol export is critically regulated by phosphate levels, in a Pho84-dependent manner.

The immediate activation of Pho84-dependent inositol export, as well as the fast response to phosphate levels, contrasts the simplistic view that Pho84 is only expressed and active under prolonged phosphate starvation. Reports from several proteomic studies demonstrated that on average ~5000 Pho84 proteins are present per cell in standard growth media (Nagaraj et al, 2012; Thakur et al, 2011; Webb et al, 2013). To take advantage of modern imaging technologies since the earlier characterisation of Pho84-GFP (Petersson et al, 1999), we revisited Pho84 dynamics by C-terminal tagging with an enhanced GFP and applying live cell imaging to track its localisation within single cells over time. Identical experimental conditions were applied as the washout assay (Fig. 5A), except that BY4741 yeast was used to avoid autofluorescence arising from mutations in the *ADE2* and *TRP1* genes in W303. Pho84-eGFP signals were detectable on the plasma membrane, vacuolar membrane and vacuolar lumen prior to washout (Fig. 5C, time 0). The intensity of the Pho84-eGFP signal varies from cell to cell, indicative of dynamic regulatory pathways. Upon inositol washout in low phosphate, Pho84-eGFP signals significantly increased as early as 15 min (Fig. 5C,D) and became highly intense and observable in all cells after 60 min (Fig. 5C). In contrast, when cells were switched to standard phosphate media without inositol, the plasma membrane Pho84-eGFP signal was initially sustained but disappeared over time (Fig. 5C,D). The imaging experiment hence clarified two features of Pho84: (1) it is present at basal levels in phosphate-replete conditions and is subject to dynamic turnover; (2) its plasma membrane abundance increased within minutes of exposure to low phosphate, supporting an additional role of phosphate in regulating the protein at the post-translational level.

We further investigated how PP-InsPs regulate inositol export by characterising *vip1Δ*. As previously reported, *PHO84* expression is abolished in *vip1Δ* (Fig. EV4A,B) (Chabert et al, 2023; Lee et al, 2007). Since we were unable to disentangle Vip1’s roles in inositol synthesis and export using the halo assay, we applied the ^13^C_6_-inositol washout assay and found that *vip1Δ* phenocopied *pho84Δ*’s export defect in both low and standard phosphate media (Fig. 5B). Hence, Vip1 regulates inositol export by controlling *PHO84* transcription.

Paradoxically, *PHO84* transcription was upregulated in *kcs1Δ* (Nishizawa et al, 2008) (Fig. EV4A), although the mutant showed an inositol export defect (Fig. 5B). We therefore considered the possibility that Kcs1 regulates Pho84 at the post-translational level. We were unable to generate a C-terminally tagged Pho84 in the *kcs1Δ* background either by genomic integration or plasmid transformation (Fig. EV4C) and therefore resorted to the generation of a plasmid-borne Pho84 under a galactose-inducible promoter. In wild-type yeast, Pho84-13myc protein was abundantly expressed 30 min after induction with galactose and underwent rapid degradation upon quenching with glucose and cycloheximide (Fig. 5E). In contrast, Pho84-13myc protein levels were dramatically reduced in *kcs1Δ* yeast upon galactose induction, suggesting that the protein may be unstable. We further analysed pGAL-Pho84-eGFP. While the addition of galactose elevated Pho84-eGFP signal equally in wild type and *kcs1Δ* cells after 30 min (Figs. 5F and EV4D), signal increase on the plasma membrane was significantly reduced in *kcs1Δ* cells (Fig. 5F and EV4D). Instead, Pho84-eGFP signals were mostly present as intense puncta in the cytoplasm of *kcs1Δ* yeast (Fig. 5F), reminiscent of Pho84 localisation under high phosphate conditions (Fig. 5C). A previous study suggested that high phosphate levels inactivate Pho84 by causing its endocytosis and degradation (Mouillon and Persson, 2005). Since *kcs1Δ* is known to abolish inorganic polyphosphate formation (Auesukaree et al, 2005; Lonetti et al, 2011) and simultaneously increase the phosphate-rich adenylate pools (Szijgyarto et al, 2011), we asked if cellular free phosphate levels are altered. While the total phosphate levels did not change in *kcs1Δ* (Fig. 5G), free phosphate levels increased more than threefold (Fig. 5H). Therefore, *kcs1Δ* creates a high phosphate condition that reduces Pho84 protein stability (Mouillon and Persson, 2005), thereby decreasing inositol export.

In summary, both Vip1 and Kcs1 kinases play central roles in coordinating phosphate and inositol metabolism, by directly regulating Pho84 at the transcriptional and post-translational levels, respectively (Fig. 5I).

### Conservation of inositol export mechanisms in mammalian cells

**Figure 6:**
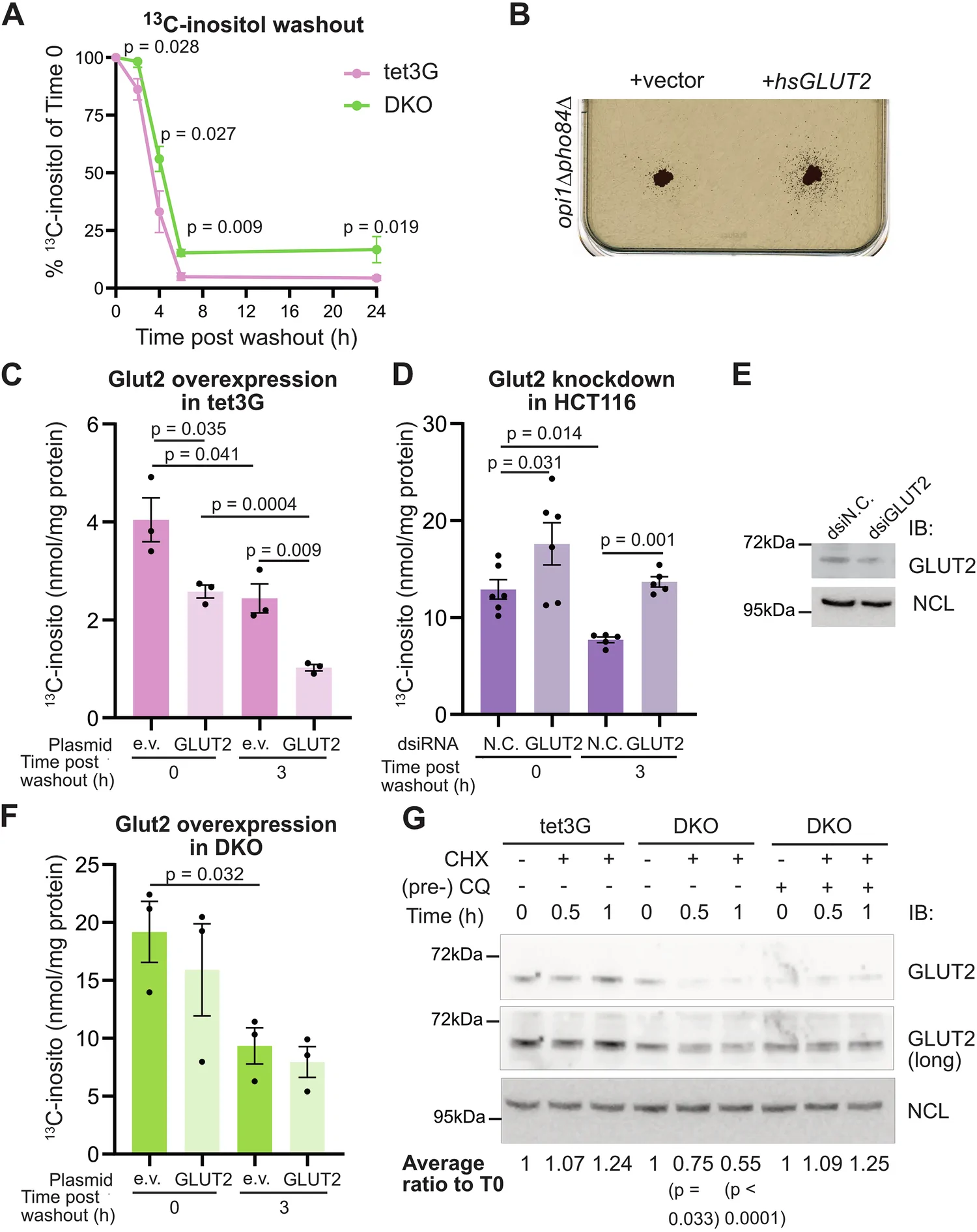
The inositol export pathway is conserved in human cells. (**A**) HCT116 colon cancer cell line exports inositol, and the rate of inositol export is reduced in the DKO cell line. The wild-type parental cell line to DKO (tet3G) and the DKO line were prelabelled with 40 µM ^13^C_6_-inositol for at least three cell divisions and subsequently switched to inositol-free media. Sugar extraction was performed at the indicated time points and inositol levels were presented as average percentage to time 0 ±  s.e.m (*n* ≥ 3 biological replicates, 2 h: *P* = 0.028, 4 h: *P* = 0.027, 6 h: *P* = 0.009, 24 h: *P* = 0.019, statistics was performed using one-tailed Student’s *t*-test). (**B**) Heterologous expression of *GLUT2* partially rescues the inositol export defect of *opi1Δpho84Δ*. *opi1Δpho84Δ* (yBS148) freshly transformed with empty vector (pBS110) or pADH-*GLUT2*-tCYC (pBS125) were analysed by halo assay. (**C**) Overexpression (O/E) of *GLUT2* decreases the steady-state level of inositol and accelerates inositol export in tet3G. Empty *eGFP* vector (pBS53, labelled as E.V.), or pCMV-*GLUT2-eGFP* (pBS141) was transfected into tet3G. ^13^C_6_-inositol labelling was performed as described in (**A**), and ^13^C_6_-inositol levels were measured prior to, or 3 h after inositol washout. (*n* ≥ 3 biological replicates, tet3G + e.v. T0 vs. tet3G + GLUT2 T0: *P* = 0.035; tet3G + e.v. T0 vs. tet3G + e.v. 3 h: *P* = 0.041; tet3G + GLUT2 T0 vs. tet3G + GLUT2 3 h: *P* = 0.0004; tet3G + e.v. 3 h vs. tet3G + GLUT2 3: *P* = 0.009, using two-tailed Student’s *t*-test). (**D**) GLUT2 knockdown increases steady-state inositol levels and slows down inositol export. dsiRNA Negative Control (NC) or of GLUT2 were transfected into HCT116 cells as described in Methods. ^13^C_6_-inositol labelling was performed as described in (**A**), and ^13^C_6_-inositol levels were measured prior to, or 3 h after inositol washout. (*n* ≥ 5 biological replicates, HCT116 + dsiNC T0 vs. HCT116 + dsiGLUT2 T0: *P* = 0.031; HCT116 +dsiNC T0 vs. HCT116+ dsiNC 3 h: *P* = 0.014, HCT116 + dsiNC 3 h vs. HCT116+ dsiNC 3 h: *P* = 0.001, using paired two-tailed Student’s *t*-test). (**E**) Glut2 protein was partially knocked down by dsiRNA. Protein extracts were analysed by anti-Glut2 or anti-NCL (loading control) immunoblotting. (**F**) Overexpression (O/E) of *GLUT2* does not change the steady-state level of inositol, or affect inositol export in DKO. DKO was transfected and analysed as described in (**C**). (*n* = 3 biological replicates, DKO+e.v. T0 vs. DKO +e.v. 3 h: *P* = 0.032, using two-tailed Student’s *t*-test). (**G**) Glut2 protein is unstable in DKO cells in a lysosome-dependent manner. Cells initially grown in the presence of 40 µM inositol were switched to inositol-free media, in the presence of 100 μg/mL cycloheximide. DKO cells were also pretreated with chloroquine for 2 h, before inositol washout in the presence of cycloheximide 100 μg/mL and 50 μM chloroquine (pre-CQ). Shown are the representative blots. The ratio of Glut2 to NCL signal was quantified and compared to T0 of each cell line/treatment (*n* ≥ 3 biological replicates, DKO CHX T0 v T30: *P* = 0.033, DKO CHX T0 v T60: *P* < 0.0001, using one-tailed Student’s *t*-test). Source data are available online for this figure.

Next, we investigated whether inositol export pathways are conserved in human cells. To analyse inositol export, we used tet3G (Wilson et al, 2019), a derivative of the colorectal cancer cell line HCT116. Tet3G cells were prelabelled with ^13^C_6_-inositol for at least 3 cell divisions and subsequently switched to inositol-free media. ^13^C_6_-inositol showed a steady decrease over time, and only 4% of ^13^C_6_-inositol remained after 6 h. No further reduction was seen between 6 and 24 h, indicating that cellular inositol levels had reached equilibrium with the media (Fig. 6A). An *ip6k1-/-ip6k2-/-* double knockout cell line (DKO) isogenic to tet3G, which was previously shown to have no detectable levels of PP-InsPs (Wilson et al, 2019), reduced the rate of ^13^C_6_-inositol exit (Fig. 6A). Hence, an inositol export pathway is present in human cells and is regulated by PP-InsPs.

**Figure EV5:**
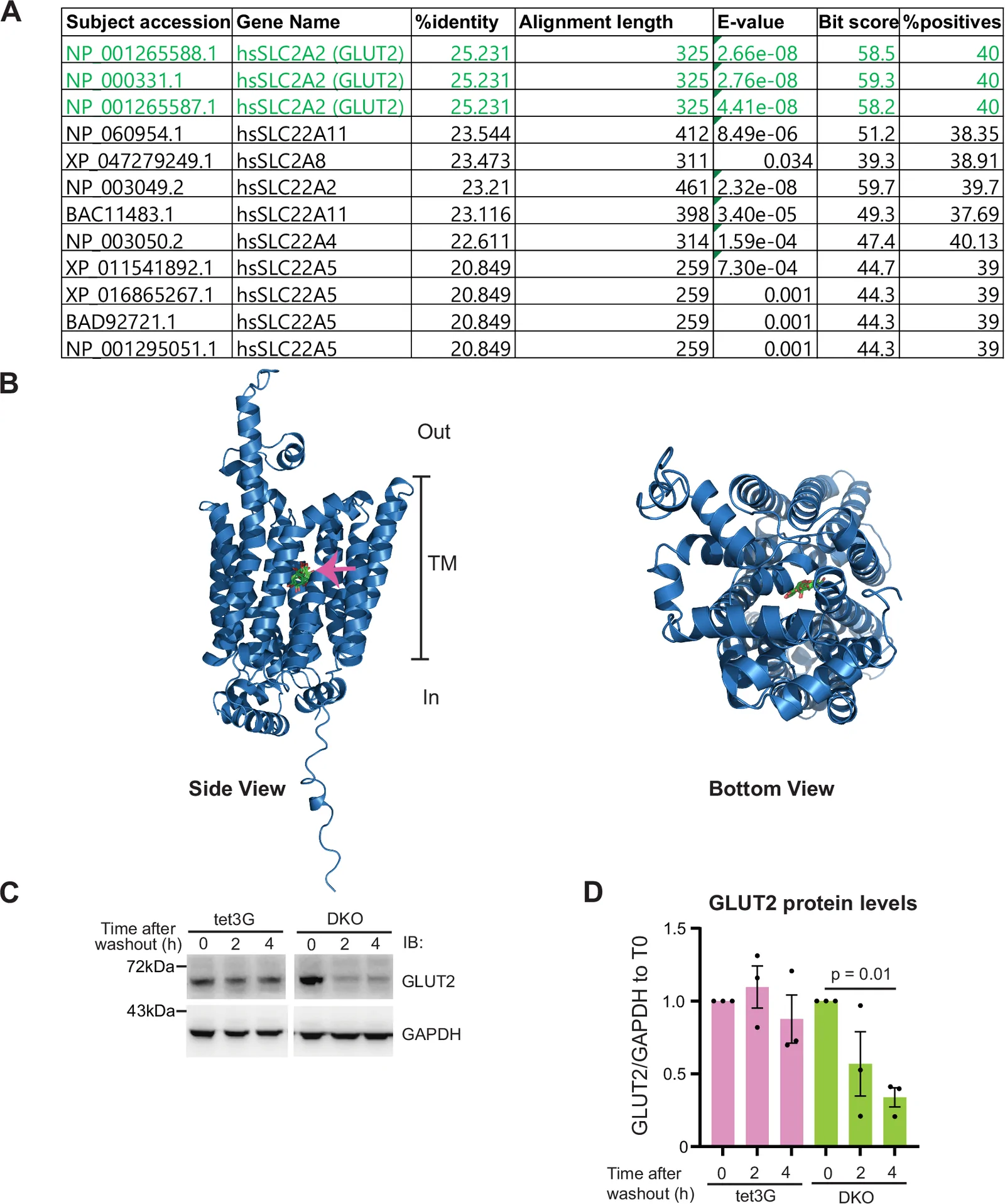
In silico analysis of the human GLUT2 protein demonstrates its potential as an inositol transporter. (**A**) A blast search of Pho84 within the human proteome revealed a number of proteins with sequence homology, with GLUT2 having the highest confidence scores. Shown is the Tree View generated by the Fast minimum evolution method. (**B**) Predicted inositol binding sites within GLUT2. Structural modelling was generated using DiffDock-L(https://neurosnap.ai). The top five inositol binding sites were found within the channel formed by the transmembrane domain, as indicated by a pink arrow. (**C**) GLUT2 protein levels are regulated by IP6K. Cells were pre-cultured in inositol-replete media and subsequently switched to inositol-free media. Protein extracts were prepared from the indicated time points and analysed by Western blotting using anti-GLUT2 or anti-GAPDH (loading control) antibodies. (**D**) Quantification of three biological repeats of (**C**). Graphs represent averages ± s.e.m (*n* = 3 biological replicates, DKO T0 vs. DKO 4 h: *P* = 0.01, using two-tailed Student’s *t*-test).

We next searched for the mammalian homologue of Pho84 and found Slc2a2 (GLUT2) had the highest confidence score (Fig. EV5A). GLUT2 was initially recognised as a low-affinity glucose transporter, but normal fasting glucose levels and glucose tolerance remained stable in *glut2*^−/−^ mice (Guillam et al, 1997; Bathina et al, 2023), suggesting that GLUT2 may have alternative functions. Structural modelling predicted inositol binding sites at the centre of the channel formed by the transmembrane helices of GLUT2 (Fig. EV5B). To test the possibility that GLUT2 exports inositol, we first expressed human GLUT2 in *opi1Δpho84Δ* yeast and observed a partial rescue of its inositol export defect (Fig. 6B). We subsequently overexpressed GLUT2 in tet3G and found it significantly reduced both steady-state intracellular inositol concentration and accelerated the loss of ^13^C_6_-inositol after inositol washout (Fig. 6C). We next examined the effect of GLUT2 knockdown on inositol export. We performed these experiments using the unmodified HCT116 cell line, which had higher steady-state inositol levels than tet3G (Fig. 6D), but a similar rate of inositol export as tet3G after washout (compare time 0 and 3 h in 6A, 6C and 6D). A dsiRNA induced knockdown of GLUT2 (Fig. 6E) significantly increased steady-state intracellular inositol concentration and slowed down the loss of ^13^C_6_-inositol after inositol washout (Fig. 6D), demonstrating that GLUT2 contributes to inositol export in vivo.

We subsequently examined how PP-InsPs might regulate GLUT2. Overexpressing GLUT2 in DKO did not reduce inositol levels in steady-state or accelerated the loss of ^13^C_6_-inositol (Fig. 6F). This suggests that like in the yeast system, PP-InsPs might regulate GLUT2 at the protein level. Indeed, while GLUT2 was stable in tet3G after inositol washout, its levels were significantly reduced in DKO (Fig. EV5C,D). We further validated DKO’s effect on GLUT2 protein levels by performing a cycloheximide chase experiment. Although GLUT2 was stable in tet3G within the duration of the experiment, its levels were significantly reduced in the DKO cells (Fig. 6G). Since previous studies suggested that GLUT2 undergoes lysosomal degradation, we studied whether DKO triggers GLUT2 degradation in a lysosome-dependent manner. Cells pretreated with a lysosomal inhibitor chloroquine, for 2 h showed a general reduction in the levels of GLUT2 in DKO (Fig. 6G, pre-CQ+), but the levels remained constant when combined with cycloheximide (Fig. 6G). Hence, PP-InsP is required to prevent lysosome-mediated degradation of GLUT2. In conclusion, we identified the human GLUT2 as an inositol exporter, and its stability is regulated by inositol pyrophosphates.

## Discussion

### Dynamic flux of inositol

Unlike other hexose sugars such as glucose and fructose, inositol is not typically metabolised by eukaryotic cells as an energy source. Instead, it functions as an osmolyte and serves as the structural backbone of signalling molecules such as PInsPs and InsPs (Su et al, 2023). These molecules modulate key cellular processes, including membrane trafficking, calcium signalling and energy metabolism (Kim et al, 2024). Consequently, inositol homoeostatic metabolism is an upstream regulator of many fundamental aspects of cell biology. Although previous works characterised the individual pathways regulating inositol levels, such as endogenous synthesis (Culbertson and Henry, 1975), import (Vawter et al, 2019), utilisation (Paulus and Kennedy, 1960) and recycling (McAllister et al, 1992), the concept of dynamic inositol efflux, as depicted in Fig. 1A, has not been considered. This lacuna is partly attributed to the lack of a robust and user-friendly method to quantify cellular levels of inositol. We recently developed a simple yet reliable sugar extraction-LC-MS workflow to measure the concentrations of the three major sugars from yeast and human cells: fructose, glucose and inositol (Su et al, 2025). Combining this workflow with *opi1Δ*-based genetic analyses, we identified a previously unknown inositol exporter, Pho84/GLUT2 and the associated regulatory mechanisms and found that many features of the inositol export pathway are evolutionarily conserved from yeast to human.

We are aware that our findings may have been affected by technical limitations associated with the halo assay and the LC-MS method used. However, our overall analysis summarising the results of the halo assay, intracellular measurement of inositol accumulation in *opi1Δ*, as well as loss of ^13^C_6_-inositol-signal, represents the most significant attempt to date to elucidate the mechanism of inositol export.

### Pho84 is a yeast inositol exporter

Inositol export was first observed in *opi1Δ* yeast more than three decades ago (Greenberg et al, 1982), followed by a yeast screen that identified many mutants with *opi1Δ*-like phenotype (Hancock et al, 2006), i.e. increased inositol export. However, challenged by the vast number of hexose transporters in eukaryotic cells, no work had been done to identify the inositol exporter. Here, we discovered that Pho84, a well-studied phosphate starvation-induced phosphate importer, can also export inositol. The two functions of Pho84 are likely to be linked, as the mutants diminishing inositol export activity also reduces its ability to uptake phosphate. Whether Pho84 acts as an inositol-phosphate antiporter, or a moonlighting protein (Espinosa-Cantu et al, 2020) will thus require future structural biology and recombinant protein-based in vitro studies.

Notably, Pho84 acts as the main inositol exporter in phosphate-restrictive conditions (Fig. 5B), which resemble the majority of natural habitats of wild yeast. In phosphate-replete conditions, however, other inositol exporter(s) is(are) active and account for ~50% of the activity. Future work will therefore be needed to identify alternative yeast inositol exporters.

### The human GLUT2 protein exports inositol

The human facilitative sugar transporter family (SLC2A) carries out the transport of sugars along concentration gradients (Holman, 2020). The H(+)-myo-inositol transporter HMIT (GLUT13), is a member of this family (Uldry et al, 2001), suggesting that other members of the family may also transport inositol. By searching for a homologue of Pho84, we discovered that GLUT2, a human low-affinity glucose transporter, is capable of exporting inositol in both yeast and human cells. Interestingly, GLUT2 was previously reported to transport glycerophospho-inositol (GroPIns) (Mariggio et al, 2006), suggesting that it has relatively low specificity and can transport both inositol- or inositol-containing GroPIns. Although the structures of other SLC2A family members, such as GLUT1 (Deng et al, 2014) and GLUT3 (Deng et al, 2015), have been solved, that of GLUT2 has not. Future structural biology and biophysical works are therefore needed to identify the structural elements within GLUT2 to carry out inositol transport.

GLUT2 shows organ-specific expression and function (Kellett, 2001). In the context of intestinal epithelium, GLUT2’s affinity for glucose exceeds that of fasting blood glucose levels, enabling it to deliver glucose into the bloodstream (Holman, 2020). Our discovery that GLUT2 is capable of exporting inositol, hence provides a mechanism by which inositol absorbed by the intestinal epithelium could be transported to the bloodstream. GLUT2 is also highly expressed in the liver and kidney. Although the prevailing view is that GLUT2 contributes to glucose reabsorption in the proximal tubule (Ghezzi et al, 2018), its capacity to export inositol commands further investigations into its role in renal physiology (Ahmad et al, 2022), especially considering the role of inositol as an osmolyte. It is also noteworthy that Fanconi-Bickel syndrome, with symptoms in the kidney, bone and liver, is a rare genetic disorder caused by mutations in GLUT2 (Santer et al, 1997). Interestingly, another form of Fanconi Syndrome, defined by defects in renal proximal tubules, is associated with mutations in inositol polyphosphate 5-phosphatase (OCRL) (Lowe, 2005). Hence, inositol metabolism may also be critical to renal physiology and disease. Lastly, GLUT2 is also expressed in neurons (Arluison et al, 2004), where PtdInsP cycles control neuronal activities. Lithium is known to deplete inositol by uncompetitive inhibition of IMPA1-based recycling of the molecule (Berridge et al, 1989; Dollins et al, 2021; Saiardi and Mudge, 2018; Su et al, 2025). This underlies the basis of lithium- based mood stabilisation for patients with bipolar disorder (BP) (Berridge et al, 1989; Su et al, 2025). However, a significant percentage of BP patients are insensitive to lithium treatment or develop resistance after long-term use (Herrera-Rivero et al, 2024). It is therefore imperative to consider whether the dynamic flux of inositol, balanced by the influx and efflux pathways (Fig. 1A), plays a role in determining the efficacy of lithium in the treatment of BP.

### Inositol exporters mediate a crosstalk network containing inositol, phosphate, and PP-InsP

Although inositol and phosphate are essential nutrients to most living organisms, excessive amount of either is detrimental to the cell, thus commanding tight regulations on their levels. Previous studies demonstrated that PP-InsPs are key regulators of phosphate metabolism, controlling cellular pools of free phosphate (Wilson et al, 2019), synthesis of phosphate-containing molecules such as polyphosphate (Lonetti et al, 2011) and ATP (Szijgyarto et al, 2011), as well as protein phosphorylation targets such as Akt (Chakraborty et al, 2010). In this work, we demonstrated a new regulatory mechanism in which PP-InsPs regulate inositol metabolism by modulating levels of the yeast inositol exporter Pho84 and the human inositol exporter GLUT2. In yeast, Vip1 regulates the PHO regulon that directly controls the transcription of Pho84 (Fig. EV4A,B). The yeast Kcs1, on the other hand, modulates cellular free phosphate levels (Fig. 5H), whose elevation was previously shown to trigger Pho84 internalisation from the plasma membrane (Mouillon and Persson, 2005). In human cells, where the PHO regulon is absent, IP6K1/2 modulate cellular free phosphate levels (Wilson et al, 2019), which correspond to an alteration of GLUT2 protein stability in DKO in inositol-free conditions (Fig. 6G). Furthermore, both our work (Figs. 5C and 6G) and previous studies (Hou et al, 2009; Mouillon and Persson, 2005) suggest that Pho84/GLUT2 proteins undergo dynamic subcellular localisation on the vacuolar/lysosomal membrane, the plasma membrane and within the vacuolar/lysosomal lumen. It was proposed that Pho84/GLUT2 are degraded in the vacuole/lysosome, respectively. Interestingly, a genetic screen looking for *opi1*- phenotype identified mutations in many genes in the AP-3 and ESCRT pathways (Hancock et al, 2006). Since these pathways are required for targeting proteins to the vacuolar/lysosomal membrane and lumen, respectively, blocking these pathways might promote Pho84/GLUT2-localisation to the plasma membrane, thus stimulating inositol export. In this context, it is worth noting that PP-InsP- driven pyrophosphorylation of the AP3B1 subunit regulates its interaction with kinesin Kif3A, which controls intracellular trafficking of the HIV-1 Gag protein and its release from the cell (Azevedo et al, 2009). Future works are therefore needed to understand whether this mechanism regulates Pho84/GLUT2 membrane targeting. More importantly, the demonstrated ability of PP-InsPs to control a fundamental aspect of inositol homoeostasis, such as inositol export from cells, warrants the need to study the dynamic flux of the inositol ring within the InsPs and PP-InsPs metabolic and signalling networks in both physiology and disease.

## Methods

**Table:** Reagents and tools table

Yeast strains		
Strain	Genotype	Source
yBS1	W303-1A	Lab collection
yBS2	W303 *kcs1Δ::LEU2*	Lab collection
yBS45	W303 *opi1Δ::LEU2*	This study
yBS67	W303 *vip1Δ*::*KANMX*	This study
yBS78	W303 *opi1Δ::LEU2 cin10Δ::URA3*	This study
yBS81	W303 *plc1Δ::KANMX*	This study
yBS88	W303 *ino1Δ::KANMX*	This study
yBS92	W303 *arg82Δ::KANMX*	This study
yBS94	W303 *ino1Δ::KANMX* + pCA45	This study
yBS109	W303 *opi1Δ::LEU2 arg82Δ::KANMX*	This study
yBS111	W303 *opi1Δ::LEU2 plc1Δ::KANMX*	This study
yBS148	W303 *opi1Δ::LEU2 pho84Δ::KANMX*	This study
yBS174	W303 *opi1Δ::LEU2 itr1Δ::SpHIS5 itr2Δ::URA3*	This study
yBS176	W303 *opi1Δ::LEU2* + pCA45	This study
yBS178	W303 *opi1Δ::LEU2* + pBS61	This study
yBS180	W303 *opi1Δ::LEU2 plc1Δ::KANMX* + pCA45	This study
yBS182	W303 *opi1Δ::LEU2 plc1Δ::KANMX* + pBS61	This study
yBS186	W303 *opi1Δ::KANMX kcs1Δ::LEU2*	This study
yBS187	W303 *opi1Δ::LEU2 ipk1Δ::KANMX*	This study
yBS189	W303 *opi1Δ::LEU2 vip1Δ::KANMX*	This study
yBS286	W303 *opi1Δ::LEU2 vvs1Δ::SpHIS*	This study
yBS335	W303 *pho84Δ::KANMX* + pBS125 (pRS316-pADH-Glut2-tCYC)	This study
yBS336	W303 *opi1Δ::LEU2 pho84Δ::KANMX* + pBS125 (pRS316-pADH-Glut2-tCYC)	This study
yBS358	BY4741 *opi1Δ::LEU2*	This study
yBS359	W303 *opi1Δ::LEU2* + pBS110 (pRS316-pADH-tCYC)	This study
yBS362	W303 *opi1Δ::LEU2 pho84Δ::KANMX* + pBS110 (pRS316-pADH-tCYC)	This study
yBS363	W303 *opi1Δ::LEU2 pho84Δ::KANMX* + pBS114 (pRS316-prom-*PHO84-flag*)	This study
yBS368	W303 *opi1Δ::LEU2* + pBS112 (pRS316-pADH-*VPS73*-tCYC)	This study
yBS375	W303 *opi1Δ::LEU2 pho84Δ::KANMX* + pBS115 (pRS316-prom-*pho84-E272Q-flag*)	This study
yBS382	W303 *opi1Δ::LEU2 pho84Δ::KANMX* + pBS121 (pRS316-prom-*pho84-Δ*(*548-553*)-Flag-tCYC)	This study
yBS392	W303 *pho84Δ::KANMX* + pBS114 (pRS316-pADH-*PHO84*-Flag)	This study
yBS393	W303 *pho84Δ::KANMX* + pBS110 (pRS316-pADH-tCYC)	This study
yBS396	W303 *pho84Δ::KANMX* + pBS114 (pRS316-pADH-*pho84*-*E272Q*-Flag)	This study
yBS398	W303 *pho84Δ::KANMX* + pBS114 (pRS316-pADH-*pho84-Δ549-553*-Flag)	This study
yBS407	W303 *PHO84-13MYC*::*TRP1*	This study
yBS421	W303 wt + pRS426-p*GAL-PHO84-13MYC*	This study
yBS422	W303 *kcs1Δ::LEU2* + pRS426-p*GAL-PHO84-13MYC*	This study
yBS423	BY4741 wild type + pRS426-p*GAL-PHO84-eGFP*	This study
yBS426	BY4741 *kcs1Δ::KANMX* + pRS426-p*GAL-PHO84-eGFP*	This study
yXZ3	BY4741 *MATa his3Δ1 leu2Δ0 met15Δ0 ura3Δ0*	Lab collection
yXZ4	BY4741 *kcs1Δ::KANMX*	This study
yXZ5	BY4741 *ino1Δ*::*KANMX*	This study
yNF1	W303 *pho84Δ::KANMX*	This study
yNF4	BY4741 *PHO84-eGFP*::CaURA3	This study
yNF10	BY4741 *vip1Δ*::*KANMX PHO84-eGFP*::CaURA3	This study
**Oligonucleotides**		
**Oligo#**	**Description**	**Sequence**
oBS3	*INO1_*KO_f	CTTGATTTATTCTGTTTCATTCCCTTTTTTTTCCAGTGAAAAAGAAGTAACAcagctgaagcttcgtacgc
oBS4	*INO1_*KO_r	GTTTTTTTATAGGTAGGCGGAAAAAGAAAAAGAGAGTCGTTGAAATGAGAgcataggccactagtggatctg
oBS11	*TUB1*_qPCR_f	GGCGATGTTCTGGATAGGATTAG
oBS12	*TUB1*_qPCR_r	CAGTACCACCACCAAGAGAATG
oBS54	*OPI1_*KO_f	TTAAAGCGTGTGTATCAGGACAGTGTTTTTAACGAAGATACTAGTCATTGcagctgaagcttcgtacgc
oBS55	*OPI1_*KO_r	GTATAATATTATTACTGGTGGTAATGCATGAAAGACCTCAATCTGTCTCGGgcataggccactagtggatctg
oBS72	*PLC1*_5’_f	CGACATAAGCAGCCTCAGGT
oBS73	*PLC1*_3’_r	TTGTCGTGGTACTTCGTCGTC
oBS74	*VIP1_*5’_f	ATTCCTTGCTGCTGTTTCGC
oBS75	*VIP1_*3’_r	CCTTGCGTTTCAGCTTCCAC
oBS76	*ARG82*_5’_f	GGAGGAGACGAGGGAGTCTT
oBS77	*ARG82_*3’_r	TGCCGAGTAAATTTCTTGCAAAC
oBS127	*PHO84*_ko_f	GATGCAACCACATTGCAC
oBS128	*PHO84*_ko_r	CGATATGCTCTGCTCTGG
oBS141	*CIN10*_rev	AGCATATCTGAAAGCCGCCCCAATGCATTATGATGCCACTTGACAACTCAgcataggccactagtggatctg
oBS148	*ITR1*_KO_f	CTACTGTATACATCTCTTTCATTTTCTTTGTTTCTGCAGTGGGTGTTAAAcagctgaagcttcgtacgc
oBS149	*ITR2*_KO_r	CTTCCTTCTTTTTTGTTCTTTATCTTTTATTTTTTGTTGGTGTAGCAGTgcataggccactagtggatctg
oBS158	*ITR1*_KO_r	CTATGTATTTGAATATTCAATTGCGTCTCCTTCCTTTTACCTCGTGAAAGGAgcataggccactagtggatctg
oBS188	hsGLUT2_for	CGCGTCGACTATGACAGAAGATAAGGTCAC
oBS189	hsGLUT2_rev	TCAGCGGCCGCTTACACAGTCTCTGTAGCTC
oBS169	*ITR2_*KO_f	GAGGCAGGACGCAGTAAAAGCAAGAAAACTGCTTTTTTTTCATGGCTGAAcagctgaagcttcgtacgc
oBS279	XhoI_t*CYC1*_f	CCGGCTCGAGGGCCGCATCATGTAA
oBS280	KpnI_t*CYC1*_r	CGGGGTACCGCAAATTAAAGCCTTCGAG
oBS298	*VPS73*_HindIII_f	CCCAAGCTTATGAATAGGATTCTATCTAGTGC
oBS299	*VPS73*_XhoI_r	CCCGCTCGAGTTAGCCGCTCCTCTTGGT
oBS300	*INO1*_qPCR_f	GGTTAGGTGGCAACAATGGC
oBS301	*INO1*_qPCR_r	TGGTTGCTTAACGCCTTCCT
oBS306	*VVS1*_KO_f	CTCTTGGAACAACAACAAAACACAGAGACTAAGGCATAACAAACTAAGTCcagctgaagcttcgtacgc
oBS307	*VVS1*_KO_r	CTAGAAAGAAGTAAAAAATAGTTGCTTAGGTATATATGACTGGCTACAGAgcataggccactagtggatctg
oBS310	*INM2*_qPCR_f	TCACGGGTTCAGAAGTGCAG
oBS311	*INM2*_qPCR_r	CACCAACCGGCACAAACATC
oBS314	*PHO84*_Ctag_f	AGAATAATGACATTGAATCTTCCAGCCCATCTCAACTTCAACATGAAGCA CGGATCCCCGGGTTAATTAA
oBS315	*PHO84*_Ctag_r	GTTTTTGTATTATTTGTTCTAGTTTACAAGTTTTAGTGCATCTTTGAGGCTTGAATTCGAGCTCGTTTAAAC
oBS319	NotI_*PHO84*_prom	TCAGCGGCCGCCAGTATTACGCACGTTGG
oBS320	XhoI_*PHO84*_Flag_r	CCCGCTCGAGTCACTTGTCGTCATCGTCTTTGTAGTCTGCTTCATGTTGAAGTTGAG
oBS327	*PHO84*_qPCR_f	GGTACCGTTCTAGGGTTGGC
oBS328	*PHO84*_qPCR_r	GCCATCTTGTTCTTGTGCGG
oBS329	*pho84-E272Q*_f	CAATCTCCTAGATATCAATTGG
oBS330	*pho84-E272Q*_r	TGGAATAGTTAATCTGAAATACAAAC
oBS335	*ITR2*_qPCR_f	TGATTACAAGTGTGGGCGCT
oBS336	*ITR2*_qPCR_r	CCAAAACTTGTGCGCGGTAA
oBS352	*pho84-*Δ(549-553)_f	AACGAGCTATACCACGATG
oBS353	*pho84-*Δ(549-553)_r	CTTTCTCTTAGTTTCTGGG
oBS367	XmaI_*PHO84*_f	TCCCCCCGGGATGAGTTCCGTCAATAAAGATAC
oBS368	SacII_ t*ADH*_r	TCCCCGCGGCTGTTATCCCTAGCGGATC
oBS373	SalI_p*GAL*_f	CGCGTCGACCGGATTAGAAGCCGCCGA
oBS374	XmaI_p*GAL*_r	TCCCCCCGGGCTCCTTGACGTTAAAGTATAG
oXZ7	*CIN10*_F2	GGGTTTTTGATAATGGAATTGATGTTCATTATGTCTTTCGTACCTACCATCGGATCCCCGGGTTAATTAA
oXZ8	*INM1*_qPCR_f	ATGTGAAAGGGGAGACCGTG
oXZ9	*INM1*_qPCR_r	GAGCCCAAGAGAGGTACAGC
oXZ10	*PIS1*_qPCR_f	CTACTGGATGCGCTAGACGG
oXZ11	*PIS1*_qPCR_r	AACACATCAAGCCAGCGGTA
oXZ12	*ITR1*_qPCR_f	GGTGGTTTCCTCCGGTTTCT
oXZ13	*ITR1*_qPCR_r	CCTCTCACGTTTTGGGGGAA
oNF1	Pho84 Ctag F5	AGAATAATGACATTGAATCTTCCAGCCCATCTCAACTTCAACATGAAGCAGGTGACGGTGCTGGTTTA
oNF2	Pho84 Ctag R3	GTTTTTGTATTATTTGTTCTAGTTTACAAGTTTTAGTGCATCTTTGAGGCTTTCGATGAATTCGAGCTCG
**Plasmids**		
**Plasmid #**	**Plasmid information**	
pBS1	pUG6 *KANMX*	
pBS2	pUG27 *SpHIS*	
pBS4	pUG72 *URA3*	
pBS5	pUG73 *LEU2*	
pBS53	eGFP-C1	
pBS61	p*ADH*-GST-hsITPK1, 2 µ, *URA3*	
pBS110	pRS316-p*ADH*-t*CYC*	
pBS112	pRS316-p*ADH*-*VPS73*-t*CYC*	
pBS114	pRS316-prom-*PHO84*-Flag-t*CYC*	
pBS115	pRS316-prom-*pho84-E272Q*-Flag-t*CYC*	
pBS121	pRS316-prom-*pho84*-Δ(549-553)-Flag-t*CYC*	
pBS122	pRS316-prom-*PHO84*-eGFP-t*CYC*	
pBS125	pRS316-p*ADH*-hs*GLUT2*-t*CYC*	
pBS132	pRS426-p*GAL*	
pBS135	pRS426-p*GAL*-*PHO84-eGFP-tADH*	
pBS136	pRS426-p*GAL*-*PHO84-13MYC-tADH*	
pBS141	pCMV-GLUT2-eGFP	
PKT209	yeGFP-CaURA	
pCA45	pADH-GST empty vector, 2 µ, *URA3*	
pYD008	pFA6a-13MYC-TRP	
**Antibodies**		
Anti-Flag M2	Merck	Cat#F1804
Anti-GAPDH	Thermo Fisher Scientific	Cat #MA5-15738
Anti-Glut2	Abcam	Cat#ab54460
Anti-MYC (9E10)	Santa Cruz Biotechnology	Cat# sc-40
Anti-Nucleolin (NCL)	Abcam	Cat#ab50279
Anti-mouse IgG, HRP-linked antibody	Santa Cruz Biotechnology	Cat#sc-2005
Anti-rabbit IgG, HRP-linked Antibody	Cell Signalling	Cat#7074P2
**Bacterial and virus strains**		
DH5α	NEB	Cat#C2987H
**Chemicals, peptides, and recombinant proteins**		
Yeast nitrogen base without amino acids or inositol	Formedium	Cat#CYN3702
Yeast nitrogen base without amino acids, inositol or phosphate	Formedium	Cat#CYN8010
CSM mix	MP Biomedical	Cat#4500-022
CSM -ura	Formedium	Cat#DCS0169
Myo-inositol	Fluka	Cat#57570
^13^C_6_-inositol	Synthesised by the Fiedler lab	
[^3^H]-inositol	Revvity	Cat#NET114A
[^32^P]-orthophosphate	Revvity	Cat#NEX053001MC
MCE membrane filter	Millipore	Cat#HAWP02500
Ultima-FLO AP liquid scintillation mixture	Revvity	Cat#6013591
Agar	Formedium	Cat#AGA03
Acetonitrile HPLC grade	Merck	Cat#10001334
Methanol HPLC grade	Merck	Cat#34860
Acidic phenol	Merck	Cat#P4682
Chloroform	VWR	Cat#22711.324
Tetrabutylammoniumhydrogen sulphate	Merck	Cat#155837
Turbo DNase	Thermo Fisher Scientific	Cat#AM1907
Superscript II reverse transcriptase	Thermo Fisher Scientific	Cat#18064014
NuPAGE 4–12% protein gel	Thermo Fisher Scientific	Cat#NP0321BOX
PVDF membrane	Bio-Rad	Cat#162-0177
Concanavalin A	Merck	Cat#C2010
Polyvinyl alcohol	Merck	Cat#341584
Foetal bovine serum	Thermo Fisher Scientific	Cat#A5256801
Dialysed foetal bovine serum	Thermo Fisher Scientific	Cat#26400-044
DMEM	Thermo Fisher Scientific	Cat#21969035
Glutamax	Thermo Fisher	Cat#35050038
	Scientific	
Sodium pyruvate	Thermo Fisher	Cat#11360070
	Scientific	
RIPA lysis buffer	Millipore	Cat#20-188
Phos-stop	Roche	Cat#4906845001
cOmplete™, EDTA-free Protease Inhibitor Cocktail	Roche	Cat#04693132001
dsiRNA negative control	Integrated DNA Technology	Cat# hs.Ri.SLC2A2.13.1
dsiRNA of GLUT2	Integrated DNA Technology	Cat#51-01-14-04
Cycloheximide	Cambridge Bioscience	Cat#C001
Chloroquine	Cambridge Bioscience	Cat#HY-17589A
**Critical commercial assays**		
Lipofectamine-3000	Thermo Fisher Scientific	Cat#L3000001
Bradford assay kit	Bio-Rad	Cat#5000006
SybrGreen mix	Bio-Rad	Cat#1725271
Pico ECL	Thermo Fisher Scientific	Cat#34577
Femto ECL	Thermo Fisher Scientific	Cat#34094
**Cell lines**		
HCT116 tet3G	Lab collection	Wilson et al, 2019
HCT116 *ip6k1*^-/-^ *ip6k2*^-/-^	Lab collection	Wilson et al, 2019
HCT116	ATCC	Ccl-247

All yeast strains, oligos, plasmids and other reagents used in this study are listed in the Reagents and tools table.

### Yeast strain construction

All knockout yeasts were generated by homologous recombination using oligos and plasmids listed in the Reagents and tools table (Gueldener et al, 2002). eGFP tag was amplified from pKT209 (Sheff and Thorn, 2004) using primers oNF1 and oNF2 and integrated to the C-terminus of *PHO84* by homologous recombination. 13MYC tag was amplified from pFA6a-13MYC-TRP1 using primers oBS314 and oBS315 and integrated to the C-terminus of *PHO84* by homologous recombination.

### Molecular cloning

Specific coding and promoter sequences were generated using oligos listed in the Reagents and tools table. The promoter sequence of *ADH* (p*ADH*) was amplified by PCR and cloned into SpeI-HindIII-digested pRS316. The terminator sequence of *CYC1* (t*CYC1*) was amplified using primers oBS279 and oBS280 by PCR and cloned into XhoI and KpnI-digested pRS316-p*ADH*. The endogenous promoter and coding sequence of *PHO84* was amplified from W303 genomic DNA using primers oBS319 and oBS320, generating a NotI-prom-*PHO84*-Flag-XhoI fragment. The PCR product was digested and ligated with NotI and XhoI-digested pRS316-p*ADH*-t*CYC1*, thus producing pRS316-prom-*PHO84*-Flag-t*CYC1* (pBS114). To generate mutants of *PHO84*, oligos listed in the Reagents and tools table were first subject to 5’ phosphorylation with T4 polynucleotide kinase (New England Biolabs), and the phosphorylated primers were used for high-fidelity PCR with Q5 polymerase, using pBS114 as a template. The PCR product was ligated using Quick ligase (New England Biolabs) and transformed into DH5α. HindIII-*VSP73*-XhoI was amplified from W303 genomic DNA using primers oBS298 and oBS299, digested and ligated with HindIII-XhoI-digested pRS316-p*ADH*-t*CYC1*, generating pRS316-p*ADH*-*VPS73*-t*CYC1* (pBS112). The endogenous promoter, *PHO84-eGFP* and t*CYC1* was amplified from the genomic DNA of *PHO84-eGFP* (yNF3) using primers oBS319 and oBS280, digested with NotI and KpnI and cloned into pRS316. p*GAL* was amplified from pYES2-NTA, using primers oBS373 and oBS374. The PCR product was digested with SalI and XmaI and cloned into pRS426. *PHO84*-*13MYC*-t*ADH* or *PHO84*-*eGFP*-t*ADH* was amplified from the genomic DNA of yBS407 or yNF4, using primers oBS367 and oBS368. XmaI-SacII digested PCR product was cloned into pRS426-pGAL, thus creating pRS426-p*GAL*-*PHO84*-*13MYC*-t*ADH* or pRS426-p*GAL*-*PHO84*-*eGFP*-t*ADH*.

Human GLUT2 was cloned by amplifying the coding sequence from the cDNA of HepG2 cell line using primers oBS188 and oBS189, digested with SalI and NotI and cloned into pCMV-myc, or a pYES2-NT/A-based vector. GLUT2-tCYC was amplified using primers oBS188 and oBS280, digested with SalI and KpnI and subcloned into pBS110, generating pRS316-pADH-GLUT2-tCYC. GLUT2 without a stop codon was amplified, digested with NheI and SalI, to replace the eGFP sequence in the eGFP-C1 plasmid. The resulting plasmid was digested with SalI and XmaI, and ligated with a PCR fragment of eGFP containing these sites, thus generating pCMV-GLUT2-eGFP.

### Yeast growth

All yeast growth media was prepared with 18 MΩ-cm ultra-pure water. Inositol-free synthetic complete (SC -ino) or dropout media (SD -ino) were made with 0.69% yeast nitrogen base without amino acid and inositol (Formedium), 1 × CSM synthetic complete or dropout supplement mix (Formedium) and 2% D-glucose. Low phosphate media was made with 0.65% yeast nitrogen base without amino acid, inositol and phosphate (Formedium), 0.2 mM potassium phosphate monobasic (Sigma), 1 × CSM synthetic complete or dropout supplement mix (Formedium) and 2% D-glucose. Solid media was made with 2% agar (Formedium) and the same composition of SC or SD as the liquid culture. SC+ino media was supplemented with the indicated concentrations of ^12^C or ^13^C_6_ inositol (Harmel et al, 2019). Most yeast growths were done at 30 °C. Exceptions were inositol washout assays, live imaging, galactose-induction experiments and phosphate uptake measurements, which were performed at room temperature (~22 °C).

### Measurement of intracellular inositol levels by LC-MS

Yeast cells were grown overnight in 25 ml SC-ino media and were harvested in mid-logarithmic phase, when A_600_ reached 0.8–1.2 OD. 20 OD cells were pelleted, washed twice with cold sterile water, before resuspending in 2 ml cold acetonitrile: methanol: water (40:40:20) in a 2 ml Eppendorf tube. After brief vortexing, tubes were placed horizontally at −20 °C overnight. Supernatant containing sugar extracts was collected after centrifuging at 4 °C for 10 min and dried in a temperature-controlled centrifuge at <45 °C in a chilled speed vacuum. LC-MS analysis was performed as previously described (Su et al, 2025), and quantification was performed by comparing to a known amount of inositol standards injected in the same sample set.

### RNA extraction and reverse transcription

Yeast strains were grown in SC-ino media, and 5–10 OD of cells in mid-logarithmic phase were harvested, washed once with diethyl pyrocarbonate (DEPC)-treated cold water and snap frozen in liquid nitrogen before storage at −80 °C. Cell pellets were resuspended in 400 µl 10 mM Tris pH 7.5, 10 mM EDTA, 0.5% SDS and mixed with 400 µl acidic phenol pH 4.2 (Sigma). The mixture was vortexed for 30 s and heated at 65 °C for 30 min with occasional vortexing. After cooling for 3 min on ice, the mixture was centrifuged for 5 min at maximum speed at 4 °C. The top layer was mixed with 50:50 acidic phenol: chloroform, vortexed and centrifuged. The final top layer was incubated with 1/10 volume 3 M sodium acetate pH 5.2 and 2.5 volumes of 100% ethanol for 1 h on ice. After centrifuging for 15 min at max speed at 4 °C, the pellet was washed once with 80% ethanol and resuspended in DEPC-treated water.

For reverse transcription, 1 µg RNA was treated with the Turbo DNA-free kit (Thermo Fisher). About 100 ng DNase-treated RNA was mixed with oligo dT (Thermo Fisher) and Superscript II reverse transcriptase (Thermo Fisher). cDNA was diluted 1:5 and analysed by qPCR using SsoAdvanced SYBR green supermix (Bio-Rad) and a CFX connect real-time system (Bio-Rad). Ct values of target genes were compared to those of *TUB1* (loading control) in the same sample using the 2^−ΔCt^ method. Statistics were performed by performing an unpaired Student’s *t*-test on the ln(2^−ΔCt^) values of at least three biological repeats.

### Measurement of bulk lipid-associated and cytosolic inositol metabolites

Fresh yeast precultures in synthetic complete (SC) media (Formedium) were used to inoculate (OD_600_ = 0.05) 5 ml of SC media containing 5 µCi/ml of [^3^H]-inositol (Revvity). Cells were grown overnight at 30 °C, collected by centrifugation for 2 min at 2000 × *g*, and washed twice in water. InsPs were extracted by adding 300 ml of 1 M perchloric acid containing 5 mM EDTA and glass beads. Cells were broken by vortexing for 5 min at 4 °C and then centrifuged for 5 min at 15,000 × *g* at 4 °C. The supernatant was transferred to a new tube, and the cell debris and beads were washed with 200 ml of 1 M perchloric acid containing 5 mM EDTA. The supernatant of the wash was pooled with the supernatant from the previous step, which together represent the water-soluble, cytosolic components. The lipids were recovered from the yeast debris by adding 500 μl of cell collection solution (50:20:2 MeOH/H_2_O/HCl), 400 μl chloroform, and 300 μl of water containing 3 mM of tetrabutylammoniumhydrogen sulphate (TBAHS). Tubes were vortexed for 30 s, incubated on ice for 15 min and then centrifuged for 3 min at 5000 × *g*. To further purify the lipid phosphoinositides, the chloroform layer was collected and washed with an equal volume of synthetic upper phase (1:48:47 chloroform/MtOH/0.6 M HCl). The tubes were vortexed for 60 s and incubated on ice for 15 min before centrifugation for 5 min at 5000 × *g*. The collected chloroform layer represents the lipid components. Aliquots of 10–50 ml of the cytosolic and lipid components were mixed with 4 ml of Ultima-FLO AP liquid scintillation mixture (Revvity), and radioactivity was measured using Tri-CARB 4910TR β-counter (PerkinElmer).

### Yeast halo assay

Starter cultures of exporter strains were grown in SC or SD -ino overnight. *ino1Δ* was grown in SC or SD media supplemented with 10 µM inositol. Strains were pelleted and washed twice with sterile deionised water. *ino1Δ* cells were spread evenly on solid media at 0.09 OD per cm^2^. 1 OD of exporter strains were pelleted, washed twice with sterile deionised water, resuspended in 500 µl water and 2.5 µl was spotted on top of *ino1Δ*. Plates were incubated at 30 °C, and images were taken after 3 days.

### Yeast viability assay

1 OD of overnight cultures of yeast grown in SC-ino media were diluted in 1 ml SC-ino and further diluted 10,000-fold. About 100 µl of the diluted culture was plated on SC-ino solid media in duplicate, and visible colonies were counted on Days 1, 2, and 3 after plating.

### Structural modelling

Structural modelling and ligand docking were performed using DiffDock-L (Corso et al, 2024) hosted on https://neurosnap.ai/.

### Pho84 Western blotting

Pre-cultures of yeast were grown in SD-Ura-ino and subsequently switched to 0.2 mM Pi SD-Ura-ino for 3 h at room temperature. 25 OD yeast cells were pelleted and incubated in 1 ml 0.2 M sodium hydroxide, 0.5% β-mercaptoethanol for 10 min. The resulting pellet was resuspended in 160 µl 8 M urea, 50 mM HEPES pH 6.8, 5% SDS, 0.5 mM EDTA and 5 mM dTT and shaken with glass beads for 3 min at 4 °C. The extracts were supplemented with 2.5% β-mercaptoethanol and bromophenol blue, heated at 70 °C for 10 min and loaded on a 4–12% NuPAGE bis-tris gel (Thermo Fisher). Proteins were transferred to a PVDF membrane (Bio-Rad) using a semi-dry transfer unit, Membranes were blocked in 5% milk in Tris-buffered saline + 0.1% Tween-20 (TBST), incubated in 1:1000 anti-Flag M2 (Merck), or 1:1000 anti-GAPDH (Thermo Fisher) and 1:5000 anti-mouse HRP (Santa Cruz Technology). 50:50 Femto and Pico ECL substrates were used (Thermo Fisher) and signal was acquired with the Alliance Q9 imaging system.

### Phosphate uptake assay

Yeast carrying the pRS316-derived plasmid of PHO84 wild type and mutated forms expressed under the control of the Pho84 promoter were grown overnight in SD-ura supplemented with 50 µM inositol. Yeast (OD_600_ = 3) were harvested and washed twice in phosphate-free SD-ura and then resuspended in 6 mL phosphate-free SD-ura + 50 µM inositol and shaken for 2 h at room temperature. Phosphate uptake was performed at room temperature and started by adding 0.1 mM potassium phosphate, pH 5.2, supplemented with 2 μCi Phosphorus-32 (^32^P) (Revvity, NEX053001MC). At the indicated time, 1 mL of yeast culture were harvested by filtration on MCE membrane filter (Millipore, HAWP02500). Membranes were washed twice with ice-cold 0.1 mM phosphate media and subsequently transferred in a 20 ml scintillation glass vials. Filter were dissolved in 5 ml of Ultima-FLO AP liquid scintillation mixture (Revvity) and radioactivity was measured using Tri-CARB 4910TR β-counter (PerkinElmer).

### Live cell imaging

Cells were cultured in standard Pi media with 50 μM *myo*-inositol until mid-log phase before being transferred to a 4 Chamber Lab-Tek^®^ Chambered #1.0 Borosilicate Coverglass Systems coated with 5 mg/ml Concanavalin A in 50 mM CaCl_2_ and 50 mM MnCl_2_. Cells were washed once, and incubated in SC-ino media with 0.2 mM or 7.3 mM phosphate. Imaging was performed in a temperature-controlled chamber at 25 °C with a Nikon Plan Apo VC 100x/1.40 Oil DIC N2 Objective on a PerkinElmer UltraVIEW VoX microscope, consisting of a Nikon Ti-E stand attached to a Yokogawa CSU-X1 Confocal Scanner Unit. Live images were acquired by a Hamamatsu C9100-13 EM-CCD Camera using the Volocity Software at a fixed rate of 15 min per timepoint across 2.5 h, with shutters managed for balanced protection and speed. XY positions were visited using the UltraVIEW XY Stage and autofocused at each point for every timepoint using the Nikon Ti PFS. At each XY position, 12 z-slices were captured across 8 μm by moving the UltraView Focus Drive upwards using both a transmitted light channel (exposure time = 300 ms, gain = 149) and a 488 nm channel (exposure time = 20 ms, laser power = 20%, gain = 149). Imaging analyses were then performed with Fiji.

### Galactose-induced PHO84 expression

Cultures were grown in SD-ura + 2% raffinose + 0.02% glucose overnight until A_600_ reached ~0.3–0.5. *PHO84-13MYC or -eGFP* expression was induced by the addition of 2% galactose for 30 min at room temperature. To perform the cycloheximide pulse-chase experiment, de novo synthesis of Pho84-13myc protein was quenched by the addition of 2% glucose and 0.25 mg/ml cycloheximide (Cambridge Bioscience). Approximately 20 OD cells were harvested, treated with 1.85 M sodium hydroxide and 7.5% β-mercaptoethanol for 20 min and precipitated with 7.5% TCA for 20 min on ice. Cells were snap frozen in liquid nitrogen and lysed in 300 µl buffer containing 6 M guanidine hydrochloride, 100 mM sodium phosphate, pH 8.0 and 10 mM Tris, pH 8.0 by beating with glass beads. Extracts were centrifuged for 20 min and supernatant was subject to buffer exchange using an Amicon 3k column, into 8 M urea, 100 mM sodium phosphate, pH 6.8 and 10 mM Tris, pH 6.8. About 50 µl extracts were then mixed with an equal volume of sample loading buffer containing 8 M urea, 200 mM Tris pH 6.8, 1 mM EDTA, 5% SDS, 0.1% bromophenoblue and 1.5% dTT, and incubated at 60 °C for 10 min. Western blotting was performed as described above, and anti-myc 9E10 (Santa Cruz Technology) was used at a 1:1000 dilution in 2% milk TBST.

### Yeast phosphate measurement

Yeast cells were grown in SC + 50 µM inositol until mid-log phase. To measure total phosphate, 0.5 OD cells were pelleted, washed twice with 1 ml sterile water, before boiling in 1 M sulfuric acid for 20 min. The samples were centrifuged for 3 min at 13,000 × *g*, and 100 µl supernatant was used for Malachite green assay (Freimoser et al, 2006).

To measure free phosphate, 2 OD cells were pelleted, washed twice with 1 ml sterile water, before resuspending in 200 µl ice-cold 0.1% NP-40. Acid-washed glass beads were pre-washed four times with 10 volumes of sterile water, on a horizontal rotator for 24 h at 4 °C. About 50 µl beads were added to the cell suspension and shaken for 2 min at 4 °C. The samples were centrifuged for 3 min at 13,000 × *g*, and 100 µl supernatant was used for Malachite green assay (Carter and Karl, 1982).

### Mammalian cell culture

All cells were grown in DMEM (Invitrogen) supplemented with 10% FBS (Sigma) and 4.5 g/l of glucose in a humidified atmosphere with 5% CO_2_. To modify inositol and/or phosphate content, a custom-made media without inositol (Thermo Fisher) was used, supplemented with indicated concentrations of inositol and 10% dialysed FBS (Sigma). Cells were rinsed once with a wash buffer (25 mM HEPES, pH 7.4, 140 mM NaCl) before incubating in the nutrient-adjusted media. All cells were used within 10 days of defrosting to obtain consistent results.

For plasmid transfection, tet3G or DKO cells were seeded in six-well plates in a custom-made medium supplemented with 40 µM ^13^C_6_-inositol and were transfected when the cells reached about 30% confluency. About 2.5 μg plasmid DNA was used with Lipofectamine 3000 (Thermo Fisher). Fresh 40 µM ^13^C_6_-inositol was given 24 h prior to the inositol washout experiment.

For dsiRNA transfection, HCT116 (ATCC) was seeded and cultured as described above. When cells reached about 20–25% confluency, 50 pmol dsiRNA of GLUT2 (Integrated DNA technology, hs.Ri.SLC2A2.13.1) or DsiNegative control (Integrated DNA technology, 51-01-14-04) complexed with Lipofectamine 3000 was added. After 18 h, an additional 37.5 pmol dsiRNA complexed with Lipofectamine 3000 was added. After 4 h, fresh 40 µM ^13^C_6_-inositol was given, and inositol measurement and washout were performed 24 h later.

Sugar extraction was performed when cells reached 80–100% confluency. Cells were washed twice with ice-cold wash buffer (25 mM HEPES pH 7.4, 140 mM NaCl), and each well was incubated with 500 µl ice-cold 40:40:20 methanol:acetonitrile:water for 10 min on ice, with gentle rotation every 2 min. Sugar extracts were dried in a temperature-controlled speed vacuum (<45 °C) and stored at −20 °C. To analyse the sugar composition, dried extracts were dissolved in 100 µl 50:50 acetonitrile:water, incubated on ice for 20 min, before being centrifuged for 10 min at 14,000 rpm at 4 °C. 10 µl extract was injected, and LC-MS was performed as described previously. The amount of inositol was quantified by comparing to the inositol standard injected on the same day and was normalised to the amount of protein in the well.

For the cycloheximide chase experiment, cells were cultured in custom-made media, 10% dialysed FBS supplemented with 40 μM inositol and fresh media was given 24 h before the experiment. Cells were washed once with phosphate-buffered saline and subsequently given inositol-free media containing 100 μg/mL cycloheximide (Cambridge Bioscience). Chloroquine-mediated lysosomal inhibition was performed by pretreating cells with 50 μM chloroquine (Cambridge Bioscience) for 2 h prior to the inositol washout in the presence of cycloheximide and chloroquine.

Cells were lysed in a cold buffer containing 25 mM bicine pH 7.6, 150 mM sodium chloride, 1× PhosSTOP™ (Roche), 1× cOmplete™, EDTA-free Protease Inhibitor Cocktail (Roche) and 2 mM sodium pyrophosphate (Merck), rotated for 20 min and sonicated twice for 10 s at 20% power. Extracts were centrifuged for 10 min at 13,000 × *g*. The soluble fraction was mixed with LDS loading buffer and 2.5% β-mercaptoethanol, and heated at 60 °C for 10 min. Western blotting was performed similarly to that described for yeast extracts. Membranes were incubated in 1:1000 diluted anti-GLUT2(Abcam) or 1:5000 diluted anti-nucleolin (Abcam) overnight and 1:10,000 diluted anti-rabbit HRP secondary (Cell Signalling) for 3 h at 4 °C .

### Statistical test

All statistical analyses, except Fig. 5D, were performed using one or two-tailed Student’s *t*-test. One-tailed tests were applied when prior knowledge on the direction of the change existed. A paired *t*-test was applied to Fig. 6D because siRNA knockdown efficiency varied in independent transfection experiments. The Mann–Whitney test was used for Fig. 5D because the data were not normally distributed.

## Supplementary information

Source data Fig. 1

Source data Fig. 2

Peer Review File

Source data Fig. 3

Source data Fig. 4

Source data Fig. 5

Source data Fig. 6

Expanded View Figures

## Data Availability

No large-scale data amenable to data repository deposition were generated in this study. The source data of this paper are collected in the following database record: biostudies:S-SCDT-10_1038-S44318-026-00888-9.
