# Supplementary material for: Pho84/GLUT2 are cellular myo-inositol exporters regulated by inositol pyrophosphates
Source: EMBO J. 2026 Aug 7;45(18):6527–56. doi: 10.1038/s44318-026-00888-9 (PMC13578697; doi:10.1038/s44318-026-00888-9)
Supplement: Supplementary file 1 [file 44318_2026_888_MOESM3_ESM.pdf]

THE EMBO JOURNAL

# Pho84/GLUT2 are cellular *myo*-inositol exporters regulated by inositol pyrophosphates

Xue Su, Xinjie Zhang, Valeria Fedeli, An-Li Ko, Jiarui Fu, Dorothea Fiedler, and Adolfo Saiardi

*Corresponding author: Adolfo Saiardi (a.saiardi@ucl.ac.uk)*

## Review Timeline:

| | |
|---|---|
| Submission Date: | 18th Dec 25 |
| Editorial Decision: | 2nd Feb 26 |
| Revision Received: | 22nd May 26 |
| Editorial Decision: | 1st Jul 26 |
| Revision Received: | 3rd Jul 26 |
| Accepted: | 22nd Jul 26 |

*Editor: Daniel Klimmeck*

## Transaction Report:

(Note: With the exception of the correction of typographical or spelling errors that could be a source of ambiguity, letters and reports are not edited. Depending on transfer agreements, referee reports obtained elsewhere may or may not be included in this compilation. Referee reports are anonymous unless the Referee chooses to sign their reports.)

## 1st Editorial Decision 2nd Feb 2026

Dear Dr Saiardi,

Thank you again for the submission of your manuscript (EMBOJ-2025-123375) to The EMBO Journal, as well as for your patience with our feedback at this time. As mentioned earlier, your study was assessed by three reviewers with expertise in inositol signaling and yeast metabolism, whose comments are enclosed below.

As you will see from the experts' reports, the referees acknowledge the analysis and potential interest and value of your findings. However, they also express important issues that will need to be conclusively addressed before they can support publication at the EMBO Journal. In more detail, the referees detail alternative assays and complementary controls which are required to strengthen the evidence for GLUT2's role as an inositol exporter, and provide unequivocal support for the claims made. Further, the reviewers raise a number of issues related to the presentation of the findings, clarity of their discussion, that would need to be conclusively addressed to achieve the level of robustness and clarity needed for The EMBO Journal.

Given the overall interest stated and broader angle of your findings, we are able to invite you to revise your manuscript experimentally to address the referees' comments. However, I need to stress that we do require strong support from the referees on a revised version of the study in order to move on to publication of the work.

I would appreciate if you could contact me during the next weeks for exchange e.g. a video call to discuss your perspective on the comments and potential plan for revisions.

Please feel free to contact me if you have any questions or need further input on the referee comments.

We generally allow three months as standard revision time. As a matter of policy, competing manuscripts published during this period will not negatively impact on our assessment of the conceptual advance presented by your study. However, we request that you contact the editor as soon as possible upon publication of any related work, to discuss how to proceed. Should you foresee a problem in meeting this three-month deadline, please let us know in advance and we may be able to grant an extension.

When submitting your revised manuscript, please carefully review the instructions below.

Please feel free to approach me any time should you have additional questions related to this.

Thank you for the opportunity to consider your work for publication.

I look forward to your revision.

Kind regards,

Daniel Klimmeck

Daniel Klimmeck, PhD
Senior Editor
The EMBO Journal

Instruction for the preparation of your revised manuscript:

1) a .docx formatted version of the manuscript text (including legends for main figures, EV figures and tables). Please make sure that the changes are highlighted to be clearly visible.

2) individual production quality figure files as .eps, .tif, .jpg (one file per figure).

3) a .docx formatted letter INCLUDING the reviewers' reports and your detailed point-by-point response to their comments. As part of the EMBO Press transparent editorial process, the point-by-point response is part of the Review Process File (RPF), which will be published alongside your paper.

4) a complete author checklist, which you can download from our author guidelines (https://wol-prod-cdn.literatumonline.com/pb-assets/embo-site/Author Checklist%20-%20EMBO%20J-1561436015657.xlsx). Please insert information in the checklist that is also reflected in the manuscript. The completed author checklist will also be part of the RPF.

5) Please note that all corresponding authors are required to supply an ORCID ID for their name upon submission of a revised manuscript.

6) It is mandatory to include a 'Data Availability' section after the Materials and Methods. Before submitting your revision, primary datasets produced in this study need to be deposited in an appropriate public database, and the accession numbers and database listed under 'Data Availability'. Please remember to provide a reviewer password if the datasets are not yet public (see https://www.embopress.org/page/journal/14602075/authorguide#datadeposition).
In case you have no data that requires deposition in a public database, please state so in this section. Note that the Data Availability Section is restricted to new primary data that are part of this study.
*** Note - All links should resolve to a page where the data can be accessed. ***

7) Our journal encourages inclusion of *data citations in the reference list* to directly cite datasets that were re-used and obtained from public databases. Data citations in the article text are distinct from normal bibliographical citations and should directly link to the database records from which the data can be accessed. In the main text, data citations are formatted as follows: "Data ref: Smith et al, 2001" or "Data ref: NCBI Sequence Read Archive PRJNA342805, 2017". In the Reference list, data citations must be labeled with "[DATASET]". A data reference must provide the database name, accession number/identifiers and a resolvable link to the landing page from which the data can be accessed at the end of the reference. Further instructions are available at .

8) At EMBO Press we ask authors to provide source data for the main manuscript figures. You will receive a separate email with instructions for providing source data with your revised manuscript, including how to upload and organize the files.

Numerical data can be provided as individual .xls or .csv files (including a tab describing the data). For 'blots' or microscopy, uncropped images should be submitted (using a zip archive or a single pdf per main figure if multiple images need to be supplied for one panel).

Additional information on source data and instruction on how to label the files are available

9) We replaced Supplementary Information with Expanded View (EV) Figures and Tables that are collapsible/expandable online (see examples in https://www.embopress.org/doi/10.15252/embj.201695874). A maximum of 5 EV Figures can be typeset. EV Figures should be cited as 'Figure EV1, Figure EV2" etc. in the text and their respective legends should be included in the main text after the legends of regular figures.

- For the figures that you do NOT wish to display as Expanded View figures, they should be bundled together with their legends in a single PDF file called *Appendix*, which should start with a short Table of Content. Appendix figures should be referred to in the main text as: "Appendix Figure S1, Appendix Figure S2" etc. See detailed instructions regarding expanded view here: .

- Additional Tables/Datasets should be labelled and referred to as Table EV1, Dataset EV1, etc. Legends have to be provided in a separate tab in case of .xls files. Alternatively, the legend can be supplied as a separate text file (README) and zipped together with the Table/Dataset file.

10) When assembling figures, please refer to our figure preparation guideline in order to ensure proper formatting and readability in print as well as on screen:
http://bit.ly/EMBOPressFigurePreparationGuideline

Please remember: Digital image enhancement is acceptable practice, as long as it accurately represents the original data and conforms to community standards. If a figure has been subjected to significant electronic manipulation, this must be noted in the figure legend or in the 'Materials and Methods' section. The editors reserve the right to request original versions of figures and the original images that were used to assemble the figure.

11) For data quantification: please specify the name of the statistical test used to generate error bars and P values, the number (n) of independent experiments (specify technical or biological replicates) underlying each data point and the test used to

calculate p-values in each figure legend. The figure legends should contain a basic description of n, P and the test applied. Graphs must include a description of the bars and the error bars (s.d., s.e.m.).

Please remember: Digital image enhancement is acceptable practice, as long as it accurately represents the original data and conforms to community standards. If a figure has been subjected to significant electronic manipulation, this must be noted in the figure legend or in the 'Materials and Methods' section. The editors reserve the right to request original versions of figures and the original images that were used to assemble the figure.

Further information is available in our Guide For Authors: https://www.embopress.org/page/journal/14602075/authorguide

We realize that it is difficult to revise to a specific deadline. In the interest of protecting the conceptual advance provided by the work, we recommend a revision within 3 months (3rd May 2026). Please discuss the revision progress ahead of this time with the editor if you require more time to complete the revisions.

------------------------------------------------

Referee #1:

myo-inositol cellular export through Pho84/GLUT2 is regulated by inositol pyrophosphates

Su et al. (Saiardi)

This study implicates Pho84, a well-established transmembrane transporter of inorganic phosphate into the cell, as an agent of inositol export from the cell - in both yeast and mammals. Given the paucity of prior studies of inositol export, this paper is a significant advance.

The manuscript is very well written, with the Introduction and opening section of Results offering a succinct but comprehensive summary of inositol synthesis and metabolism, focusing on budding yeast and the role of Kcs1 kinase that synthesizes 5-IP7.

They make use of a yeast opi1Δ strain that excretes inositol into the medium and a growth halo assay of excretion to find that kcs1Δ reduces the extent of inositol export. Export is also impaired by deletion of "upstream" enzymes in inositol polyphosphate synthesis Plc1 and Arg82.

Taking a candidate gene approach on the assumption that a hexose transporter akin to the inositol importers Itr1/2 would be a likely inositol exporter, they find that pho84Δ reduces inositol excretion by opi1Δ cells while significantly increasing intracellular inositol.

Using a docking model to pinpoint a candidate inositol binding site on the cytoplasmic side. Of Pho84, the deleted a TIEEL peptide, expressed this mutant Pho84 in opi1Δ pho84Δ cells and find that it failed to rescue the export defect.

Comment/Concern: This experiment lacks a key control in which they determine whether the ΔTIEEL mutation impacts the inorganic phosphate transport activity of Pho84. One wants to know if it is a separation of function mutation.

They then introduce an ostensibly more direct assay of export by loading cells with overnight with exogenous 13C-inositol, washing with inositol free medium, and then tracking the intracellular level of 13C-inositol as a function of time thereafter.

Comment: This is a loss-of-signal assay. It is not clear why they do not measure 13C-inositol in the medium, which is, in principle, a more sensitive gain of signal assay. (In the same vein, why not use 3H-inositol and count radioactivity?)

The export assay indicates that inositol export is enhanced by low phosphate concentration in the medium, which correlated with the finding that Pho84 expression on the cell membrane increased during phosphate limitation.

Extending the scope of mammalian cells, they tested the candidate human Pho84homolog GLUT2 (a glucose transporter) by expressing it in yeast opi1Δ pho84Δ cells, where it partially complemented the export defect in the halo assay. Overexpressing GLUT2 in human HCT116 cells was found to reduce intracellular inositol concentration, which is interpreted as reflecting increased export.

Comment/Concern: The inferences would be more solid if they made an CRISP-knockout cells line lacking GLUT2 and showed it displayed reduced inositol export.

The Discussion section asserts (line 406) that "Pho84 is THE yeast inositol exporter". Yet it is clear that Pho84 deletion reduces but does not abolish inositol excretion, which implies that there is at least one other means by which inositol escapes from yeast cells (as they note on line 417). The section title should be changed to "Pho84 is a yeast inositol exporter"

Minor point:

Line 275: don't you mean "Pho84 deletion downregulates inositol export" ?

Referee #2:

In the manuscript by Su, et al., the authors reported that inositol export is regulated by inositol pyrophosphates via Pho84/GLUT2 in yeast and human cells. Using an inositol overproducing yeast model opi1Δ yeast cells, the authors first found knockout of kcs1 results in increased intracellular inositol levels with inhibited expression of enzyme in de novo inositol synthesis, which leads to their investigation of inositol export using Halo assay. Indeed, kcs1 is required for inositol export. Furthermore, they found inositol export is regulated by inositol pyrophosphates by screening the mutants lacking enzymes for inositol pyrophosphate synthesis. Importantly, pho84 was identified as inositol exporter using structure modeling and halo assay with pho84 mutants. The authors subsequently employed a wild-type yeast model to investigate the distinct functions of Kcs1 and Vip1 in the regulation of Pho84 with standard or low Pi conditions. Finally, human GLUT2 was found to mediate inositol export, and its expression level is controlled by inositol pyrophosphates although the latter only slightly affect inositol export in human cells. Overall, the manuscript is well written, straightforward and clear. It is very novel to link inositol pyrophosphates with inositol export and identifying pho84/GLUT2 mediating inositol export.
Major concerns:
The inositol export in opi1Δ yeast models was done by halo assay which is not a great quantitative assay. Also, how does the proliferation/viability of the exporter strain affect the halo formation, although same amount cells were used for different strains? For example, do opi1Δkcs1Δ cells and kcs1Δ cells proliferate at similar rate and survive similarly on the plate? If one strain has more cells viable on the plate which will export more inositol in total. Is it possible to direct measure inositol export to culture medium by LC-MS since intracellular inositol levels can be done?
Data from Figure 4 and 5 clearly showed that Pho84 is important for inositol export. However, without pho84 in opi1Δ cell, halo size reduced to 40% (Figure 4A,B), suggesting opi1Δ pho84Δ cell can still export inositol. Furthermore, in figure 5B, pho84Δ cells export 50% of 13C inositol in 30min. These data indicate pho84 is not the only inositol exporter or pho84 functions as a regulator for inositol export. Despite membrane localization of pho84 and its importance for inositol export, more detailed structure analysis using recombinant protein is required to determine if pho84/GLUT2 is an exporter.
In figure EV4C, cellular inositol level was not changed in pho84Δ cell, and Figure 5B shows 13C inositol export was reduced. Is pho84 also involved inositol synthesis or utilization?
In Figure 6C, it would be helpful to examine inositol export for cells overexpressing GLUT2, if GLUT2 knockout cells are not available.
Minor points:
Line 275, "Pho84 downregulates inositol export" should be loss of Pho84 downregulates inositol export.

Referee #3:

General Summary
Su et al. identify Pho84 in yeast and GLUT2 in mammalian cells as inositol exporters regulated by PP-InsPs. Using an opi1Δ background with LC-MS quantification and genetic screening, they show that Kcs1 and Vip1 control export through distinct mechanisms-Kcs1 post-translationally, Vip1 at the transcriptional level. The findings extend to mammalian cells, supporting evolutionary conservation. The work fills a longstanding gap in our understanding of inositol homeostasis and connects PP-InsP signaling to both phosphate and inositol metabolism. This is a strong study identifying the first inositol exporters in yeast and mammals. The genetic dissection through the InsP pathway is thorough, and the $^{13}C_6$-washout assay effectively separates synthesis from export. Structural modeling paired with functional validation (E272Q, Δ549-553 mutants) supports direct inositol binding.

Concerns
The model proposes that KCS1 deletion elevates free phosphate, which triggers Pho84 degradation. This is plausible but could be more strongly supported. The authors report difficulty generating kcs1Δ PHO84-eGFP strains due to apparent toxicity (Fig. EV5C), yet endogenous PHO84 transcript levels are elevated in kcs1Δ. This could be clarified. Direct quantification of Pho84 protein in kcs1Δ would strengthen the model. Alternatively, testing whether exogenous phosphate reproduces the kcs1Δ export defect would be informative.

The data support GLUT2 as an inositol exporter, but its regulation is less well defined than for Pho84. GLUT2 protein decreases in DKO cells after washout (Fig. 6D-E)-is this proteasomal or lysosomal? Since Pho84 undergoes vacuolar degradation, clarifying whether GLUT2 follows an analogous route would strengthen the evolutionary comparison.

Minor comments:

- BY4741 and W303 show different basal inositol levels (Fig. EV1C-D). A note on whether this reflects export capacity differences would be helpful.
- In Fig. 5B, pho84Δ retains ~50% export activity under standard phosphate conditions, indicating additional transporter(s). Any candidates from the phylogenetic screens?

**Manuscript EMBOJ-2025-123375**

**Point To Point reply**

We truly appreciate the reviewers' positive judgments and insightful suggestions. We are delighted that they found our work to be "a significant advance" (Referee #1), "very novel" (Referee #2), and fulfilling "a longstanding gap" (Referee #3). In the revised version of our manuscript, we addressed their concerns as detailed below.

**Referee #1:**
*myo-inositol cellular export through Pho84/GLUT2 is regulated by inositol pyrophosphates Su et al. (Saiardi)*
*This study implicates Pho84, a well-established transmembrane transporter of inorganic phosphate into the cell, as an agent of inositol export from the cell - in both yeast and mammals. Given the paucity of prior studies of inositol export, this paper is a significant advance. The manuscript is very well written, with the Introduction and opening section of Results offering a succinct but comprehensive summary of inositol synthesis and metabolism, focusing on budding yeast and the role of Kcs1 kinase that synthesizes 5-IP7. They make use of a yeast opi1Δ strain that excretes inositol into the medium and a growth halo assay of excretion to find that kcs1Δ reduces the extent of inositol export. Export is also impaired by deletion of "upstream" enzymes in inositol polyphosphate synthesis Plc1 and Arg82.*
*Taking a candidate gene approach on the assumption that a hexose transporter akin to the inositol importers Itr1/2 would be a likely inositol exporter, they find that pho84Δ reduces inositol excretion by opi1Δ cells while significantly increasing intracellular inositol.*

*Using a docking model to pinpoint a candidate inositol binding site on the cytoplasmic side. Of Pho84, the deleted a TIEEL peptide, expressed this mutant Pho84 in opi1Δ pho84Δ cells and find that it failed to rescue the export defect.*

***Comment/Concern:*** *This experiment lacks a key control in which they determine whether the ΔTIEEL mutation impacts the inorganic phosphate transport activity of Pho84. One wants to know if it is a separation of function mutation.*

**Answer:** We appreciate the reviewer's comment and performed a $^{32}$P uptake assay accordingly (new Figure 4I). We found that both the E272Q and the ΔTIEEL mutants showed partially reduced ability to uptake phosphate. Given that they also showed partially reduced ability to export inositol, we concluded that Pho84 might act as a phosphate-inositol antiporter, or coordinate their opposite transport via an indirect mechanism worthy of future exploration. This point was discussed at the end of results section 'Inositol exporter identified as Pho84' as well as the discussion section 'Pho84 is a yeast inositol exporter'.

***Comment:*** *They then introduce an ostensibly more direct assay of export by loading cells with overnight with exogenous 13C-inositol, washing with inositol free medium, and then tracking the intracellular level of 13C-inositol as a function of time thereafter.*
*This is a loss-of-signal assay. It is not clear why they do not measure 13C-inositol in the medium, which is, in principle, a more sensitive gain of signal assay. (In the same vein, why not use 3H-inositol and count radioactivity?)*

**Answer:** We appreciate the reviewer's comment. However, the current LC-MS technology does not allow the detection of extracellular inositol, because the high concentration of glucose (2%, corresponding to 110mM) in the media would saturate the column compromising the resolving power and not allowing a reliable detection of inositol. The application of $^{3}$H-inositol for elucidating its export is hindered by the requirement of utilizing

substantial amounts of radioactivity to preload cells. The growing regulations and limitations surrounding the use of radioactivity compel us to seek alternative methods whenever feasible. In fact, this study was only made possible through the innovative LC-MS sugar separation technique we developed last year. Although we acknowledge that our experimental conditions are not ideal, our implementation of three distinct methods: the halo assay, the measurement of inositol accumulation in a steady state, and the $^{13}C_6$-inositol loss-of-signal experiment, constitutes a comprehensive and multifaceted approach that has enabled us to identify the mechanism of inositol export. We have incorporated these points in the first paragraph of the discussion section in our revised manuscript.

*The export assay indicates that inositol export is enhanced by low phosphate concentration in the medium, which correlated with the finding that Pho84 expression on the cell membrane increased during phosphate limitation. Extending the scope of mammalian cells, they tested the candidate human Pho84homolog GLUT2 (a glucose transporter) by expressing it in yeast opi1Δ pho84Δ cells, where it partially complemented the export defect in the halo assay. Overexpressing GLUT2 in human HCT116 cells was found to reduce intracellular inositol concentration, which is interpreted as reflecting increased export.*
***Comment/Concern:*** *The inferences would be more solid if they made an CRISP-knockout cells line lacking GLUT2 and showed it displayed reduced inositol export.*

**Answer:** We appreciate the reviewer's comment. Given the time frame of the revision, we performed GLUT2 knockdown using dsiRNA, and found that even a partial reduction of GLUT2 proteins led to significantly increased steady-state levels of inositol and reduced rate of inositol export (new Fig. 6D-6E).

***Comment:*** *The Discussion section asserts (line 406) that "Pho84 is THE yeast inositol exporter". Yet it is clear that Pho84 deletion reduces but does not abolish inositol excretion, which implies that there is at least one other means by which inositol escapes from yeast cells (as they note on line 417). The section title should be changed to "Pho84 is a yeast inositol exporter"*

**Answer:** We appreciate the reviewer's comment and have changed the text accordingly.

***Minor point:***

*Line 275: don't you mean "Pho84 deletion downregulates inositol export" ?*

**Answer:** We appreciate the reviewer's comment and have changed the text accordingly.

**Referee #2:**
*In the manuscript by Su, et al., the authors reported that inositol export is regulated by inositol pyrophosphates via Pho84/GLUT2 in yeast and human cells. Using an inositol overproducing yeast model opi1Δ yeast cells, the authors first found knockout of kcs1 results in increased intracellular inositol levels with inhibited expression of enzyme in de novo inositol synthesis, which leads to their investigation of inositol export using Halo assay. Indeed, kcs1 is required for inositol export. Furthermore, they found inositol export is regulated by inositol pyrophosphates by screening the mutants lacking enzymes for inositol pyrophosphate synthesis. Importantly, pho84 was identified as inositol exporter using structure modeling and halo assay with pho84 mutants. The authors subsequently employed a*

*wild-type yeast model to investigate the distinct functions of Kcs1 and Vip1 in the regulation of Pho84 with standard or low Pi conditions. Finally, human GLUT2 was found to mediate inositol export, and its expression level is controlled by inositol pyrophosphates although the latter only slightly affect inositol export in human cells. Overall, the manuscript is well written, straightforward and clear. It is very novel to link inositol pyrophosphates with inositol export and identifying pho84/GLUT2 mediating inositol export.*

***Comments:*** *The inositol export in opi1Δ yeast models was done by halo assay which is not a great quantitative assay. Also, how does the proliferation/viability of the exporter strain affect the halo formation, although same amount cells were used for different strains? For example, do opi1Δkcs1Δ cells and kcs1Δ cells proliferate at similar rate and survive similarly on the plate? If one strain has more cells viable on the plate which will export more inositol in total. Is it possible to direct measure inositol export to culture medium by LC-MS since intracellular inositol levels can be done?*

**Answer:** We agree with the reviewer that the halo assay could be influenced by other factors such as viability and growth rate. We therefore performed a colony formation assay for our most important strains *opi1Δkcs1Δ* and *opi1Δpho84Δ.* To best mimic the condition used in the halo assay, we plated equal OD of cells on SC-ino solid media and counted the number of visible colonies on day 1, 2 and 3. The results are presented as the new EV1E and EV3C.

As the reviewer rightly pointed out, *opi1Δkcs1Δ* indeed showed a slower growth rate compared to *opi1Δ* on day 1 and 2, but equal number of colonies were observed for both on day 3 (new Fig EV1E). This suggests that the halo assay may have exaggerated the export defect of *opi1Δkcs1Δ.* However, the steady-state inositol levels and the $^{13}$C-inositol loss-of-signal assay were both free from any bias caused by growth rate, as equal number of actively dividing cells were used for these two assays. We agree that every technique has its limitations, and therefore discussed the confounding factors of the halo assay in the revised manuscript, while maintaining our argument that inositol export is defective in *kcs1*Δ mutants (line 197-202).

Interestingly, when we performed the same assay for *opi1Δpho84Δ*, we found that although this strain showed a defect in growth rate on day 1, it caught up with *opi1Δ* on later dates and actually showed a 50% increase in colony numbers compared to *opi1Δ* on day 3 (new Fig EV3C). This result means that the halo assay actually underestimated the export defect of *opi1Δpho84Δ*, and explains the difference between the mild halo assay phenotype and the dramatic increase in inositol levels we observed by LC-MS analysis. We discussed this finding thoroughly in the revised manuscript (line 272-276).

We agree with the reviewer that it would have been ideal to measure inositol released into the media. However, the current LC-MS technology does not yet permit such analysis, because the high concentration of glucose (2% corresponding to 110mM) in the media would saturate the column compromising the resolving power and not allowing a reliable detection of inositol. We added this explanation to the discussion section of the revised manuscript (line 453-457).

***Comment:*** *Data from Figure 4 and 5 clearly showed that Pho84 is important for inositol export. However, without pho84 in opi1Δ cell, halo size reduced to 40% (Figure 4A,B), suggesting opi1Δ pho84Δ cell can still export inositol. Furthermore, in figure 5B, pho84Δ cells export 50% of 13C inositol in 30min. These data indicate pho84 is not the only inositol exporter or pho84 functions as a regulator for inositol export. Despite membrane localization of pho84 and its importance for inositol export, more detailed structure analysis using recombinant protein is required to determine if pho84/GLUT2 is an exporter.*

**Answer:** We agree with the reviewer's concern. It is indeed evolutionarily important for each biological system to be supported by redundant pathways. We thoroughly discussed these points in the Discussion section of the manuscript.

However, identifying additional transporters or detailed structural studies of the two transporters are beyond the scope of this study. In fact, while groups specializing in sugar transporters have successfully resolved the co-crystal structure of GLUT1, 3 and 4 with glucose, they have not done so with GLUT2. It is highly likely that the previous attempts failed because GLUT2 instead binds inositol. Therefore, it is important to provide *in vivo* data to the research field, such that structural biologists could re-direct their efforts to understand how GLUT2 transports inositol.

***Comment:*** *In figure EV3C, cellular inositol level was not changed in pho84Δ cell, and Figure 5B shows 13C inositol export was reduced. Is pho84 also involved inositol synthesis or utilization?*

**Answer:** The inositol measurements presented in EV3D (newly labelled as) and Fig. 5B cannot be directly compared because they were done under different experimental conditions and were intended to convey different messages.

In Figure EV3D, the yeast strains did not contain high concentrations of inositol inside the cell, because 1) they did not carry *OPI1* mutation, hence no elevation of endogenous synthesis in a continuous manner; 2) they were grown in inositol-free media, i.e. not loaded with extracellular inositol. As a result, inositol levels in *pho84Δ* yeast had reached an equilibrium with the surrounding media (ino-), and so no accumulation of inositol is observed inside the cells. Figure 5B and the halo assays, in comparison, were performed under a condition where intracellular inositol levels were elevated, either by preloading with $^{13}C$ inositol (Figure 5B), or *OPI1* mutation (halo assays). An inositol concentration gradient was also artificially generated by switching to inositol-free media in both Figure 5B and halo assays, thus allowing us to observe the inositol export defect of *pho84Δ*.

Additionally, we have demonstrated in EV3E that *pho84Δ* cells did not change the expression of genes that are directly involved in the synthesis or utilization of inositol. Therefore, while Pho84 is responsible for the export of inositol from the cell, it does not play a role in regulating other aspects of inositol homeostasis.

We think the confusion may have arisen from insufficient description of our assays, and so we added a few clarification sentences in our revised manuscript to help readers understand our work.

***Comment:*** *In Figure 6C, it would be helpful to examine inositol export for cells overexpressing GLUT2, if GLUT2 knockout cells are not available.*

**Answer:** We agree with the reviewer's comment and have coupled the overexpression experiment with the washout assay. In agreement with our hypothesis, overexpression of GLUT2 significantly accelerated inositol export in tet3G (HCT116 parental) but not in the isogenic DKO cells (new Fig. 6C, 6F). We also performed GLUT2 knockdown using dsiRNA, and found that even a partial reduction of GLUT2 proteins led to significantly increased steady-state levels of inositol and reduced rate of inositol export (new Fig. 6D-6E).

***Minor points:***

*Line 275, "Pho84 downregulates inositol export" should be loss of Pho84 downregulates inositol export.*

**Answer:** We appreciate the reviewer's comment and have changed the text accordingly.

**Referee #3:**

*General Summary*

*Su et al. identify Pho84 in yeast and GLUT2 in mammalian cells as inositol exporters regulated by PP-InsPs. Using an opi1Δ background with LC-MS quantification and genetic screening, they show that Kcs1 and Vip1 control export through distinct mechanisms-Kcs1 post-translationally, Vip1 at the transcriptional level. The findings extend to mammalian cells, supporting evolutionary conservation. The work fills a longstanding gap in our understanding of inositol homeostasis and connects PP-InsP signaling to both phosphate and inositol metabolism. This is a strong study identifying the first inositol exporters in yeast and mammals. The genetic dissection through the InsP pathway is thorough, and the $^{13}C_6$-washout assay effectively separates synthesis from export. Structural modeling paired with functional validation (E272Q, Δ549-553 mutants) supports direct inositol binding.*

***Comment:*** *The model proposes that KCS1 deletion elevates free phosphate, which triggers Pho84 degradation. This is plausible but could be more strongly supported. The authors report difficulty generating kcs1Δ PHO84-eGFP strains due to apparent toxicity (Fig. EV4C), yet endogenous PHO84 transcript levels are elevated in kcs1Δ. This could be clarified. Direct quantification of Pho84 protein in kcs1Δ would strengthen the model. Alternatively, testing whether exogenous phosphate reproduces the kcs1Δ export defect would be informative.*
*13C labelling + High Pi?*

**Answer:** We agree with the reviewer's comment and have performed two different experiments to clarify the point. We constructed pGAL-PHO84-13MYC or pGAL-PHO84-eGFP, which allowed us to observe Pho84 protein in a galactose-inducible system devoid of confounding effects on transcription modulation by Kcs1. We observed that Pho84-13MYC protein was highly unstable in *kcs1Δ* (new Figure 5E) and Pho84-eGFP plasma membrane localisation was significantly reduced in *kcs1Δ* (new Figure 5F, EV4D), reminiscent of the effect of high phosphate (Figure 5C).

***Comment:*** *The data support GLUT2 as an inositol exporter, but its regulation is less well defined than for Pho84. GLUT2 protein decreases in DKO cells after washout (Fig. 6D-E)-is this proteasomal or lysosomal? Since Pho84 undergoes vacuolar degradation, clarifying whether GLUT2 follows an analogous route would strengthen the evolutionary comparison.*

**Answer:** We agree with the reviewer's comment and performed a cycloheximide chase experiment coupled with lysosomal inhibition with chloroquine (new Figure 6G). As expected, GLUT2 was unstable in the presence of cycloheximide in DKO. Although pre-incubation with chloroquine generally decreased GLUT2 levels, its presence rescued GLUT2 instability upon inositol washout (new Figure 6G). Hence, PP-InsP appears to block the lysosomal degradation pathway of GLUT2.

**Minor comments:**

• *BY4741 and W303 show different basal inositol levels (Fig. EV1C-D). A note on whether this reflects export capacity differences would be helpful.*

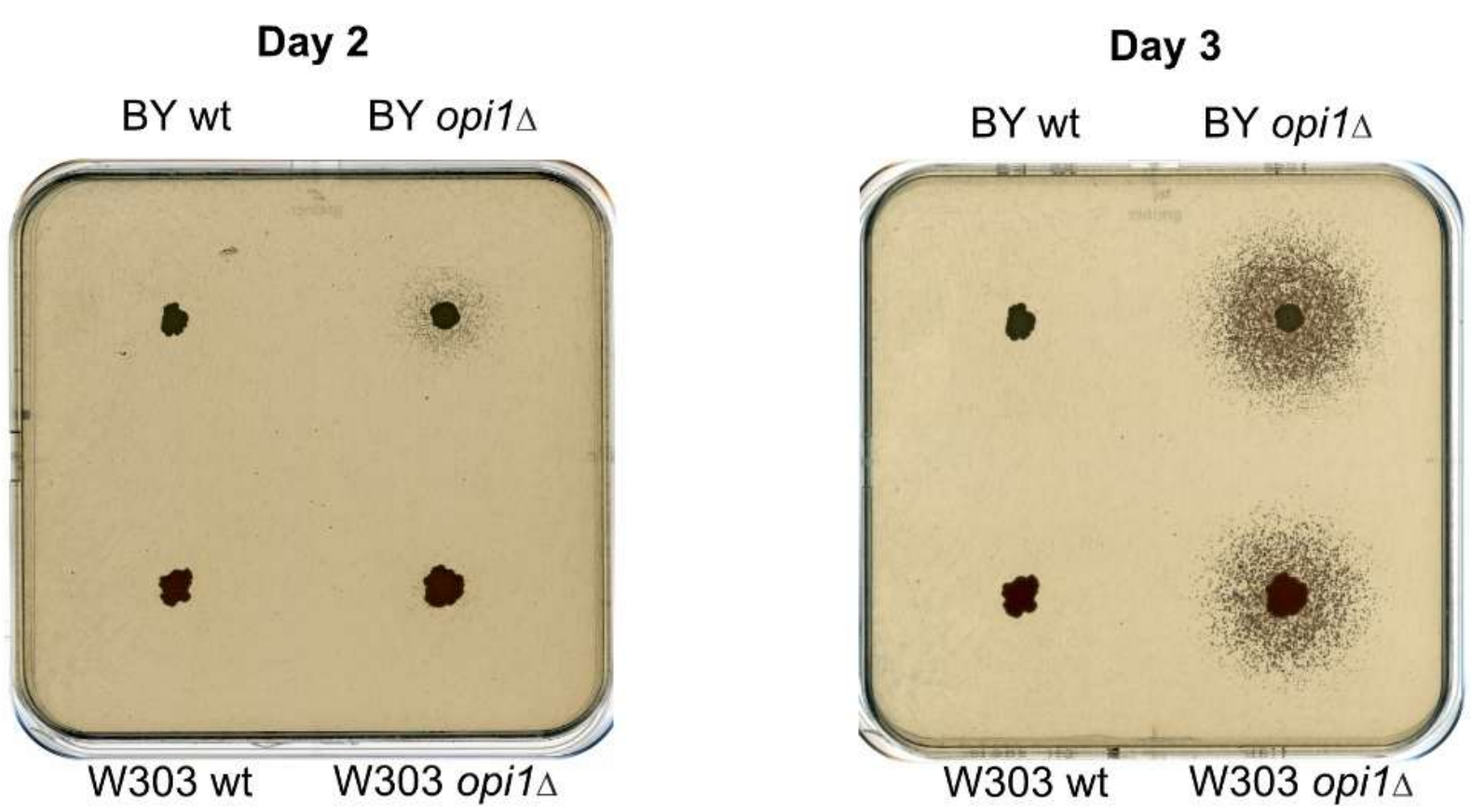


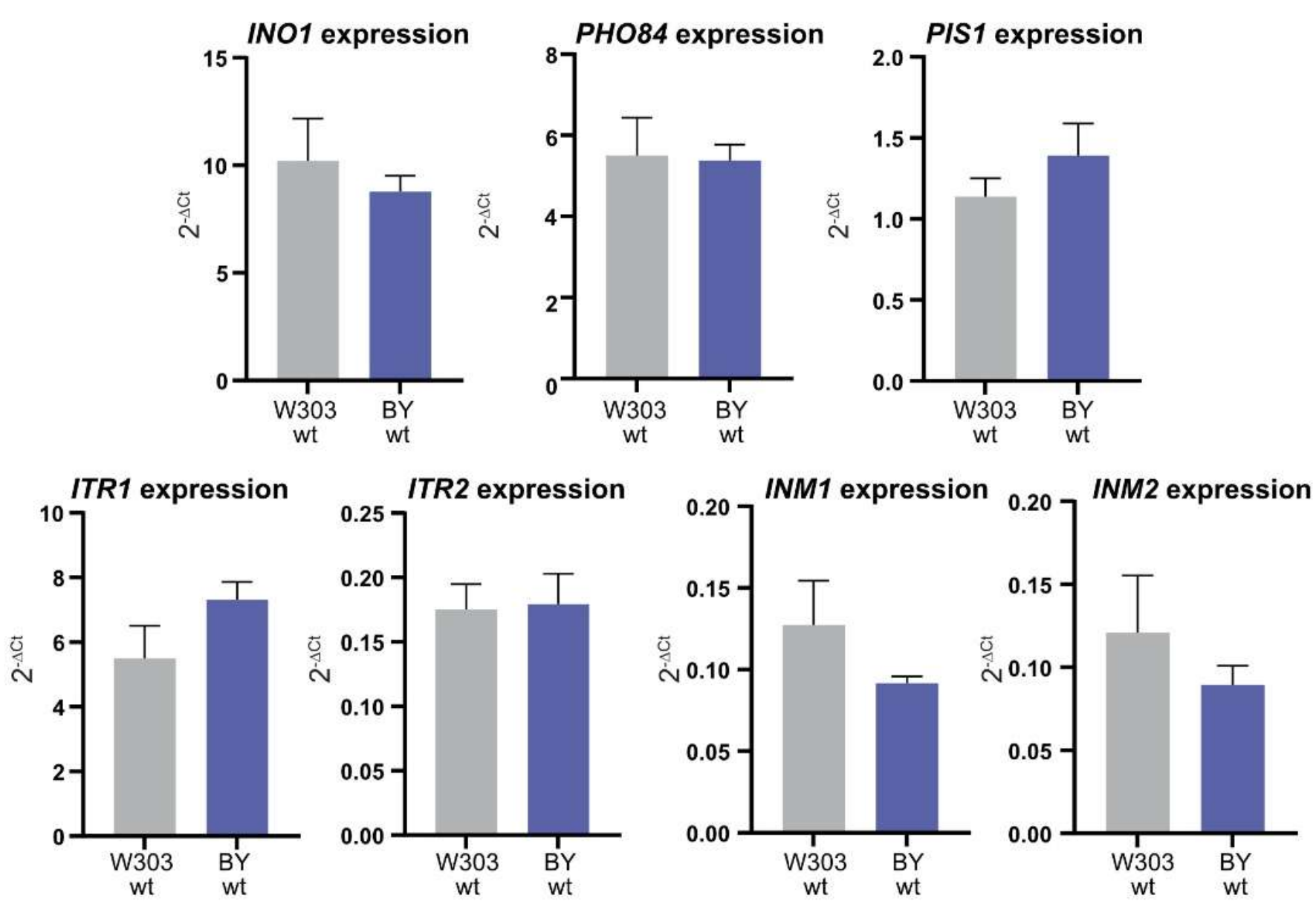


This is an interesting point. To address this point, we performed a halo assay comparing the two yeast background and observed that the BY4741 *opi1*Δ yeast did not show any defect in

exporting inositol and even accelerated the rate of inositol export compared to W303 *opi1Δ* (see above). We also performed RT-qPCRs comparing gene expression in inositol metabolic pathways and did not observe any significant difference between W303 and BY wild type strains (see above). Hence the difference in inositol levels in the two strains is most likely caused by differences in the kinetics of glucose metabolism, the characterization of which is beyond the scope of this study. We provide this analysis here, but not in the paper as yeast strain comparative analysis (well-known and discussed/referenced in the text) would alter the linearity of our manuscript.

• *In Fig. 5B, pho84Δ retains ~50% export activity under standard phosphate conditions, indicating additional transporter(s). Any candidates from the phylogenetic screens?*

**Answer:** We agree with the reviewer's point. It is indeed evolutionarily advantageous for cells to be supported by redundant pathways. We discussed these points in the Discussion section of the manuscript. However, we prefer not to speculate at this point, as further works will be needed to identify any additional transporters.

Adolfo Saiardi

Bessie Su

Dear Adolfo,

Thank you again for sending us your amended manuscript (EMBOJ-2025-123375R) for reconsideration by The EMBO Journal, together with a point-by-point response to the issues raised during peer-review. Please accept my apologies for the unusual protraction in getting back to you due to delayed expert input and detailed discussion in the editorial team. Your revised study was sent back to the referees for their scientific reassessment, and we have received detailed re-reports from all of them, which I enclose below. As you will see, the reviewers state that the work has been satisfactorily enhanced by the revisions and they can now support publication at the EMBO Journal, pending minor revision.

Thus, we are pleased to inform you that your manuscript has been accepted in principle for publication in The EMBO Journal.

Please carefully consider the remaining minor points still raised by referees #2 and #3 by adjusting the discussion of the results in the manuscript text where appropriate.

Also, we now need you to take care of a number of minor issues related to formatting and data annotation, as detailed below.

Please submit a revised version of the manuscript using the link enclosed below.

As you might have seen on our web page, every paper at the EMBO Journal includes a 'Synopsis', displayed on the html and freely accessible to all readers. The synopsis includes a 'model' figure as well as 2-5 one-short-sentence bullet points that summarize the article. I would appreciate if you could provide this figure and the bullet points.

Thank you again for giving us the chance to consider your manuscript for The EMBO Journal, I look forward to hearing from you and receiving your final revised version of the manuscript.

Best regards,

Daniel

Daniel Klimmeck PhD
Senior Editor
The EMBO Journal

Formatting changes required for the revised version of the manuscript:

>> Author Contributions: Remove the author contributions information from the manuscript text. Note that CRediT has replaced the traditional author contributions section as of now because it offers a systematic machine-readable author contributions format that allows for more effective research assessment. and use the free text boxes beneath each contributing author's name to add specific details on the author's contribution.

More information is available in our guide to authors.
https://www.embopress.org/page/journal/14602075/authorguide

>> Provide a completed Author Checklist.
Currently the Source Data Checklist is confused with the Author Checklist.

>> Funding: merge with Acknowledgments.

>> Section order should be corrected as follows: title page with complete author information, abstract, keywords, introduction,

results, discussion, methods, data availability section, acknowledgements, disclosure and competing interests statement, references, main figure legends, tables, expanded figure legends.´.

>> Data availability section: please adjust the text to 'No large-scale data amenable to data repository deposition were generated in this study.'

>> Add a separate 'Statistical Analysis' section to the Methods part, detailing the algorithms and statistical tests applied.

>> Please provide a complete set of source data files. Please upload the checklist as a related manuscript file and labeled "Source Data Checklist".

>> Remove the figures from the manuscript text and only leave the legends.

>> The supplementary information should also be removed from the manuscript text: supplemental figures should be renamed "Figure EV1" etc and uploaded as individual files. The legends should be added to the manuscript text, after the main figure legends and under the heading "Expanded View Figure Legends". Supplementary tables should be uploaded as separate files and renamed Table EV1 - Table EV3. Suppl table should be reflected in the reagents and tools table and can be deleted.

>> Consider additional changes and comments from our production team as indicated below:

- Figure legends:

1. Please note that the figure EV-1F is mislabeled as figure EV-1E, respectively in the manuscript. This needs to be rectified.
2. Please note that the exact p values are not provided in the legends of 1C,D; 2C; 3C,D,F,G; 4B,C,G,H,I; 5B,D,H; 6A,C,D,F; EV-1C,E,F; EV-2A,B; EV-3C; EV-4A,D; EV-5D.
3. Please indicate the statistical test used for data analysis in the legends of figures EV-3C; EV-5D.
4. Please note that information related to n is missing in the legends EV-3C,D,E.
5. Please note that the error bars are not defined in the legends of 5B,G,H; EV-3C,D,E; EV-5D.

Further information is available in our Guide For Authors: https://link.springer.com/journal/44318/submission-guidelines

------------------------------------------------

Referee #1:

The authors have addressed all my concerns with the initial version via inclusion of new results and text modifications.

Referee #2:

All previous comments have been addressed. One more comment from the revised version: In line 427-430, the description of Fig 6G is not quite clear. CHX treatment alone caused a reduction of Glut2 over time in DKO cells, and Glut2 levels remain stable when combined treatment with CQ, indicating lysosome-dependent degradation of Glut2 in DKO cells. Therefore, PP-IPs are required to prevent lysosomal degradation of Glut2.

Referee #3:

I thank the authors for a thorough and responsive revision. My two principal concerns have been satisfactorily addressed: the new $^{32}P$ uptake assay (Fig. 4I) provides the phosphate-transport control I requested for the inositol-export mutants, and the GLUT2 knockdown (Fig. 6D-E) establishes necessity rather than sufficiency alone in mammalian cells. The section title change and the minor textual points are also resolved.

One additional correction, which falls outside my original comments but I flag as it concerns a central conclusion. In the Results (p. 18), the sentence "Hence, PP-InsP is required for lysosome-mediated degradation of GLUT2" seems to be stated backwards relative to the data. Fig. 6G shows GLUT2 is destabilized in DKO cells (PP-InsP absent) and rescued by chloroquine, indicating that PP-InsP prevents lysosomal degradation rather than being required for it. Notably, the authors' own response to Referee #3 states the relationship correctly ("PP-InsP appears to block the lysosomal degradation pathway of GLUT2"), so this is a text error rather than a conceptual one.

With that correction, I am happy to recommend acceptance.

The authors addressed the remaining editorial issues.

Dear Adolfo,

Thank you for submitting the revised version of your manuscript. I have now evaluated your amended manuscript and concluded that the remaining minor concerns have been sufficiently addressed.

I am thus pleased to inform you that your manuscript has been accepted for publication in the EMBO Journal.

Your manuscript will be processed for publication by EMBO Press. It will be copy edited and you will receive page proofs prior to publication. Please note that you will be contacted by Springer Nature Author Services to complete licensing and payment information.

Please note that it is The EMBO Journal policy for the transcript of the editorial process (containing referee reports and your response letter) to be published as an online supplement to each paper. If you do NOT want this, you will need to inform the Editorial Office via email immediately. More information is available here: https://www.embopress.org/transparent-process#Review_Process

You may qualify for financial assistance for your publication charges - either via a Springer Nature fully open access agreement or an EMBO initiative. Check your eligibility: https://www.embopress.org/page/journal/14602075/authorguide#chargesguide

Should you be planning a Press Release on your article, please notify the production team via this form in order to coordinate publication and release dates: https://forms.cloud.microsoft/e/eH4A8JVP6g

If you have any questions, please do not hesitate to contact the Editorial Office. Thank you for your contribution to The EMBO Journal.

Thank you again for this contribution to The EMBO Journal and congratulations on a successful publication! Please consider us again in the future for your most exciting work.

Best regards,

Daniel

Daniel Klimmeck, PhD
Senior Editor
The EMBO Journal
EMBO
Postfach 1022-40
Meyerhofstrasse 1
D-69117 Heidelberg
contact@embojournal.org
------------------------------------------------

*****
Please note that it is The EMBO Journal policy for the transcript of the editorial process (containing referee reports and your response letters) to be published as an online supplement to each paper. If you should prefer removal of any referee-only figures included in the point-by-point response(s), e.g. because they may still be used for future publication or because they have been reproduced from published work by others, please do let us know immediately via response email.

## EMBO Press Author Checklist

| | |
|---|---|
| Corresponding Author Name: | |
| Journal Submitted to: | |
| Manuscript Number: | |

**USEFUL LINKS FOR COMPLETING THIS FORM**
The EMBO Journal - Author Guidelines
EMBO Reports - Author Guidelines
Molecular Systems Biology - Author Guidelines
EMBO Molecular Medicine - Author Guidelines

**Reporting Checklist for Life Science Articles (updated January**
This checklist is adapted from Materials Design Analysis Reporting (MDAR) Checklist for Authors. MDAR establishes a minimum set of requirements in transparent reporting in the life sciences (see Statement of Task: 10.31222/osf.io/9sm4x). Please follow the journal's guidelines in preparing your
**Please note that a copy of this checklist will be published alongside your article.**

**Abridged guidelines for figures**
**1. Data**
The data shown in figures should satisfy the following conditions:
- the data were obtained and processed according to the field's best practice and are presented to reflect the results of the experiments in an accurate and unbiased manner.
- ideally, figure panels should include only measurements that are directly comparable to each other and obtained with the same assay.
- plots include clearly labeled error bars for independent experiments and sample sizes. Unless justified, error bars should not be shown for technical
- if n<5, the individual data points from each experiment should be plotted. Any statistical test employed should be justified.
- Source Data should be included to report the data underlying figures according to the guidelines set out in the authorship guidelines on Data

**2. Captions**
Each figure caption should contain the following information, for each panel where they are relevant:
- a specification of the experimental system investigated (eg cell line, species name).
- the assay(s) and method(s) used to carry out the reported observations and measurements.
- an explicit mention of the biological and chemical entity(ies) that are being measured.
- an explicit mention of the biological and chemical entity(ies) that are altered/varied/perturbed in a controlled manner.
- the exact sample size (n) for each experimental group/condition, given as a number, not a range;
- a description of the sample collection allowing the reader to understand whether the samples represent technical or biological replicates (including how many animals, litters, cultures, etc.).
- a statement of how many times the experiment shown was independently replicated in the laboratory.
- definitions of statistical methods and measures:
  - common tests, such as t-test (please specify whether paired vs. unpaired), simple $\chi^2$ tests, Wilcoxon and Mann-Whitney tests, can be unambiguously identified by name only, but more complex techniques should be described in the methods section;
  - are tests one-sided or two-sided?
  - are there adjustments for multiple comparisons?
  - exact statistical test results, e.g., P values = x but not P values < x;
  - definition of 'center values' as median or average;
  - definition of error bars as s.d. or s.e.m.

**Please complete ALL of the questions below.**
**Select "Not Applicable" only when the requested information is not relevant for your study.**

**Materials**

| Newly Created Materials | Information included in the manuscript? | In which section is the information available? (Reagents and Tools Table, Materials and Methods, Figures, Data Availability Section) |
|---|---|---|
| New materials and reagents need to be available; do any restrictions apply? | Not Applicable | Reagents and Tools Table |

| Antibodies | Information included in the manuscript? | In which section is the information available? (Reagents and Tools Table, Materials and Methods, Figures, Data Availability Section) |
|---|---|---|
| For **antibodies** provide the following information:<br>- Commercial antibodies: RRID (if possible) or supplier name, catalogue number and or/clone number<br>- Non-commercial: RRID or citation | Yes | Reagents and Tools Table |

| DNA and RNA sequences | Information included in the manuscript? | In which section is the information available? (Reagents and Tools Table, Materials and Methods, Figures, Data Availability Section) |
|---|---|---|
| **Short novel DNA or RNA including primers, probes**: provide the sequences. | Yes | Reagents and Tools Table |

| Cell materials | Information included in the manuscript? | In which section is the information available? (Reagents and Tools Table, Materials and Methods, Figures, Data Availability Section) |
|---|---|---|
| **Cell lines:** Provide species information, strain. Provide accession number in repository **OR** supplier name, catalog number, clone number, and/**OR** RRID. | Yes | Reagents and Tools Table |
| **Primary cultures:** Provide species, strain, sex of origin, genetic modification status. | Not Applicable | |
| Report if the cell lines were recently **authenticated** (e.g., by STR profiling) and tested for mycoplasma contamination. | Not Applicable | |

| Experimental animals | Information included in the manuscript? | In which section is the information available? (Reagents and Tools Table, Materials and Methods, Figures, Data Availability Section) |
|---|---|---|
| **Laboratory animals or Model organisms:** Provide species, strain, sex, age, genetic modification status. Provide accession number in repository **OR** supplier name, catalog number, clone number, **OR** RRID. | Yes | Reagents and Tools Table |
| **Animal observed in or captured from the field:** Provide species, sex, and age where possible. | Not Applicable | |
| Please detail **housing and husbandry conditions**. | Not Applicable | |

| Plants and microbes | Information included in the manuscript? | In which section is the information available? (Reagents and Tools Table, Materials and Methods, Figures, Data Availability Section) |
|---|---|---|
| **Plants:** provide species and strain, ecotype and cultivar where relevant, unique accession number if available, and source (including location for collected wild specimens). | Not Applicable | |
| **Microbes:** provide species and strain, unique accession number if available, and source. | Yes | Reagents and Tools Table |

| Human research participants | Information included in the manuscript? | In which section is the information available? (Reagents and Tools Table, Materials and Methods, Figures, Data Availability Section) |
|---|---|---|
| If collected and within the bounds of privacy constraints report on age, sex and gender or ethnicity for all study participants. | Not Applicable | |

| Core facilities | Information included in the manuscript? | In which section is the information available? (Reagents and Tools Table, Materials and Methods, Figures, Data Availability Section) |
|---|---|---|
| If your work benefited from core facilities, was their service mentioned in the acknowledgments section? | Not Applicable | |

**Design**

| Study protocol | Information included in the manuscript? | In which section is the information available? (Reagents and Tools Table, Materials and Methods, Figures, Data Availability Section) |
|---|---|---|
| If study protocol has been **pre-registered, provide DOI in the manuscript**. For clinical trials, provide the trial registration number **OR** cite DOI. | Not Applicable | |
| Report the **clinical trial registration number** (at ClinicalTrials.gov or equivalent), where applicable. | Not Applicable | |

| Laboratory protocol | Information included in the manuscript? | In which section is the information available? (Reagents and Tools Table, Materials and Methods, Figures, Data Availability Section) |
|---|---|---|
| Provide DOI OR other citation details if **external detailed step-by-step protocols** are available. | Not Applicable | |

| Experimental study design and statistics | Information included in the manuscript? | In which section is the information available? (Reagents and Tools Table, Materials and Methods, Figures, Data Availability Section) |
|---|---|---|
| Include a statement about **sample size** estimate even if no statistical methods were used. | Not Applicable | |
| Were any steps taken to minimize the effects of subjective bias when allocating animals/samples to treatment (e.g. **randomization procedure**)? If yes, have they been described? | Not Applicable | |
| Include a statement about **blinding** even if no blinding was done. | Not Applicable | |
| Describe **inclusion/exclusion criteria** if samples or animals were excluded from the analysis. Were the criteria pre-established? If sample or data points were omitted from analysis, report if this was due to attrition or intentional exclusion and provide justification. | Not Applicable | |
| For every figure, are **statistical tests** justified as appropriate? Do the data meet the assumptions of the tests (e.g., normal distribution)? Describe any methods used to assess it. Is there an estimate of variation within each group of data? Is the variance similar between the groups that are being statistically compared? | Yes | Figures |

| Sample definition and in-laboratory replication | Information included in the manuscript? | In which section is the information available? (Reagents and Tools Table, Materials and Methods, Figures, Data Availability Section) |
|---|---|---|
| In the figure legends: state number of times the experiment was **replicated** in laboratory. | Yes | Figures |
| In the figure legends: define whether data describe **technical or biological replicates**. | Yes | Figures |

## Ethics

| Ethics | Information included in the manuscript? | In which section is the information available? (Reagents and Tools Table, Materials and Methods, Figures, Data Availability Section) |
|---|---|---|
| Studies involving **human participants**: State details of **authority granting ethics approval** (IRB or equivalent committee(s), provide reference number for approval. | Not Applicable | |
| Studies involving **human participants**: Include a statement confirming that **informed consent** was obtained from all subjects and that the experiments conformed to the principles set out in the WMA Declaration of Helsinki and the Department of Health and Human Services Belmont Report. | Not Applicable | |
| Studies involving **human participants: For publication of patient photos**, include a statement confirming that consent to publish was obtained. | Not Applicable | |
| Studies involving experimental **animals**: State details of **authority granting ethics approval** (IRB or equivalent committee(s), provide reference number for approval. Include a statement of compliance with ethical regulations. | Not Applicable | |
| Studies involving **specimen and field samples:** State if relevant **permits** obtained, provide details of authority approving study; if none were required, explain why. | Not Applicable | |

| Dual Use Research of Concern (DURC) | Information included in the manuscript? | In which section is the information available? (Reagents and Tools Table, Materials and Methods, Figures, Data Availability Section) |
|---|---|---|
| Could your study fall under dual use research restrictions? Please check biosecurity documents and list of **select agents and toxins** (CDC): https://www.selectagents.gov/sat/list.htm | Not Applicable | |
| If you used a select agent, is the security level of the lab appropriate and reported in the manuscript? | Not Applicable | |
| If a study is subject to dual use research of concern regulations, is the name of the **authority granting approval and reference number** for the regulatory approval provided in the manuscript? | Not Applicable | |

## Reporting

The MDAR framework recommends adoption of discipline-specific guidelines, established and endorsed through community initiatives. Journals have their own policy about requiring specific guidelines and recommendations to complement MDAR.

| Adherence to community standards | Information included in the manuscript? | In which section is the information available? (Reagents and Tools Table, Materials and Methods, Figures, Data Availability Section) |
|---|---|---|
| State if relevant guidelines or checklists (e.g., **ICMJE, MIBBI, ARRIVE, PRISMA**) have been followed or provided. | Not Applicable | |
| For **tumor marker prognostic studies,** we recommend that you follow the **REMARK** reporting guidelines (see link list at top right). See author guidelines, under 'Reporting Guidelines'. Please confirm you have followed these guidelines. | Not Applicable | |
| For **phase II and III randomized controlled trials**, please refer to the **CONSORT** flow diagram (see link list at top right) and submit the CONSORT checklist (see link list at top right) with your submission. See author guidelines, under 'Reporting Guidelines'. Please confirm you have submitted this list. | Not Applicable | |

## Data Availability

| Data availability | Information included in the manuscript? | In which section is the information available? (Reagents and Tools Table, Materials and Methods, Figures, Data Availability Section) |
|---|---|---|
| Have **primary datasets** been deposited according to the journal's guidelines (see 'Data Deposition' section) and the respective accession numbers provided in the Data Availability Section? | Not Applicable | |
| Were **human clinical and genomic datasets** deposited in a public access-controlled repository in accordance to ethical obligations to the patients and to the applicable consent agreement? | Not Applicable | |
| Are **computational models** that are central and integral to a study available without restrictions in a machine-readable form? Were the relevant accession numbers or links provided? | Not Applicable | |
| If publicly available data were reused, provide the respective **data citations in the reference list**. | Not Applicable | |
