# Supplementary material for: Pho84/GLUT2 are cellular myo-inositol exporters regulated by inositol pyrophosphates
Source: EMBO J. 2026 Aug 7;45(18):6527–56. doi: 10.1038/s44318-026-00888-9 (PMC13578697; doi:10.1038/s44318-026-00888-9)
Supplement: Supplementary file 2 [file 44318_2026_888_MOESM8_ESM.pdf]

Expanded View Figures

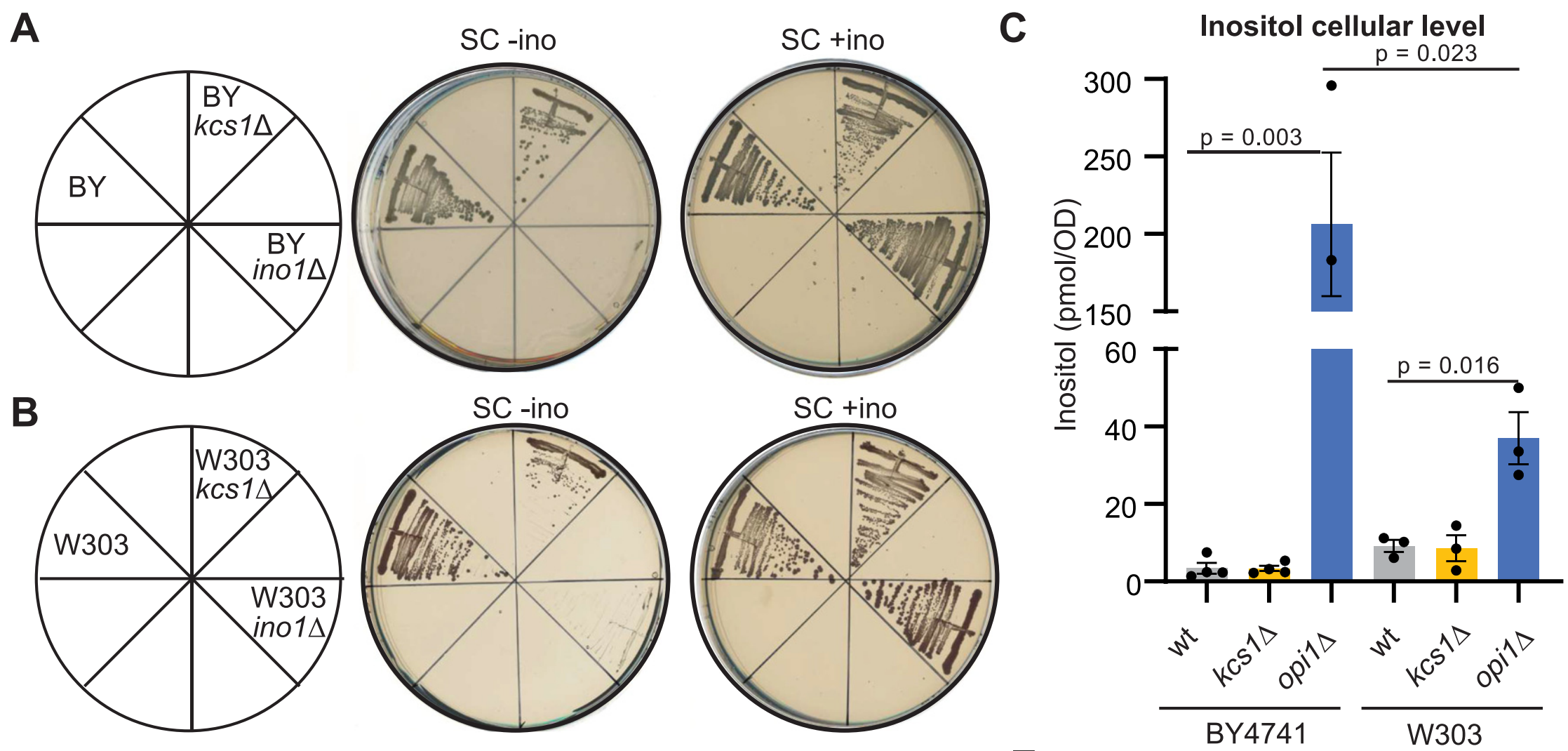

D

Strain	Genotype	Average cellular inositol level ± SEM (pmol/OD)
BY4741	Wild type	3.43±1.61
	kcs1Δ	3.32±0.82
	opi1Δ	206.22±46.46
W303	Wild type	9.21±1.56
	kcs1Δ	8.61±3.36
	opi1Δ	37.04±6.72

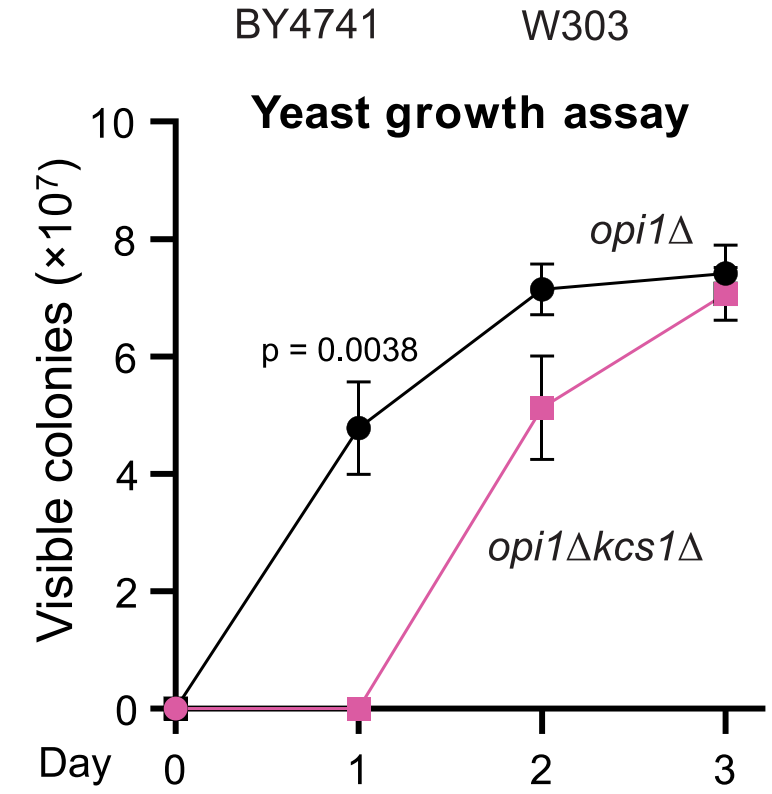

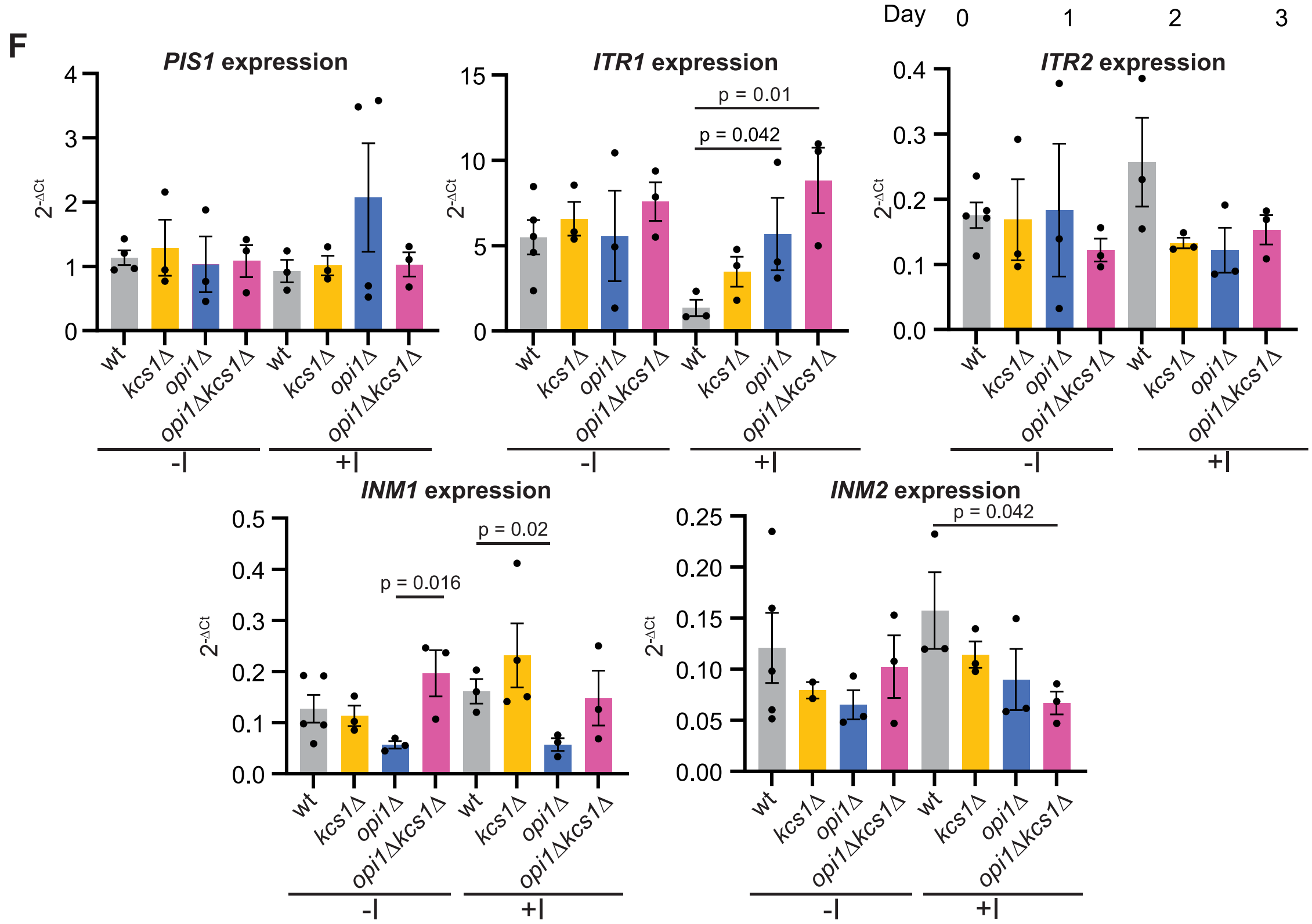

Figure EV1. Phenotypic evaluation of *kcs1Δ* and *opi1Δkcs1Δ* yeast.

(**A**) Backcrossed BY4741 (abbreviated as BY) *kcs1Δ* yeast is inositol prototrophic. Wild-type (yXZ3), *kcs1Δ* (yXZ4) and *ino1Δ* (yXZ5) yeast cells were streaked on synthetic complete media with or without 10 μM *myo*-inositol (abbreviated as SC-ino or SC+ino), as indicated in the schematics. (**B**) W303 *kcs1Δ* yeast is inositol prototrophic. Wild-type W303 (yBS1), *kcs1Δ* (yBS2) and *ino1Δ* (yBS88) yeast cells were streaked on SC-ino or SC+ino, as indicated in the schematics. (**C**) Intracellular inositol levels are unchanged in the *kcs1Δ* mutants. Yeast strains used in (**A**, **B**) were grown to mid-log phase in SC-ino media, and sugar extraction was performed as described in Methods. BY4741 *opi1Δ* (yBS358) and W303 *opi1Δ* (yBS45) yeast were similarly analysed as positive controls for increased intracellular inositol levels. Graphs represent averages ± s.e.m ($n \geq 3$ biological replicates, BY4741 wt vs. BY4741 *opi1Δ*: $P = 0.003$, W303 wt vs. W303 *opi1Δ*: $P = 0.016$, BY4741 *opi1Δ* vs. W303 *opi1Δ* $P = 0.023$, using two-tailed Student's *t*-test). (**D**) Summary of LC-MS results of EV1C. (**E**) *opi1Δkcs1Δ* shows reduced growth rate but maintained viability compared to *opi1Δ*. W303 *opi1Δ* (yBS45) and *opi1Δkcs1Δ* (yBS186) were plated on SC-ino plates as described in Methods. Graphs represent averages ± s.e.m ($n = 3$ biological replicates, Day 1: $P = 0.0038$, using two-tailed Student's t *t*-test). (**F**) Analysis of key genes controlling inositol metabolism revealed changes in *INM1* and *ITR1* levels in *opi1Δkcs1Δ* yeast. Strains and growth conditions were described in Fig. 1C. RNA extraction and RT-qPCR were performed as described in Methods. Graphs represent averages ± s.e.m ($n \geq 3$ biological replicates, *ITR1* expression in wt + I vs. *opi1Δ* + I: $P = 0.042$, wt + I vs. *opi1Δ kcs1Δ* + I: $P = 0.01$, *INM1* expression in *opi1Δ* -I vs. *opi1Δkcs1Δ* -I: P = 0.016, wt + I vs. *opi1Δ* + I: $P = 0.02$, *INM2* expression in wt + I vs. *opi1Δ kcs1Δ* + I: $P = 0.042$, using two-tailed Student's *t*-test on the $\ln(2^{-\Delta Ct})$ values).

 © The Author(s)

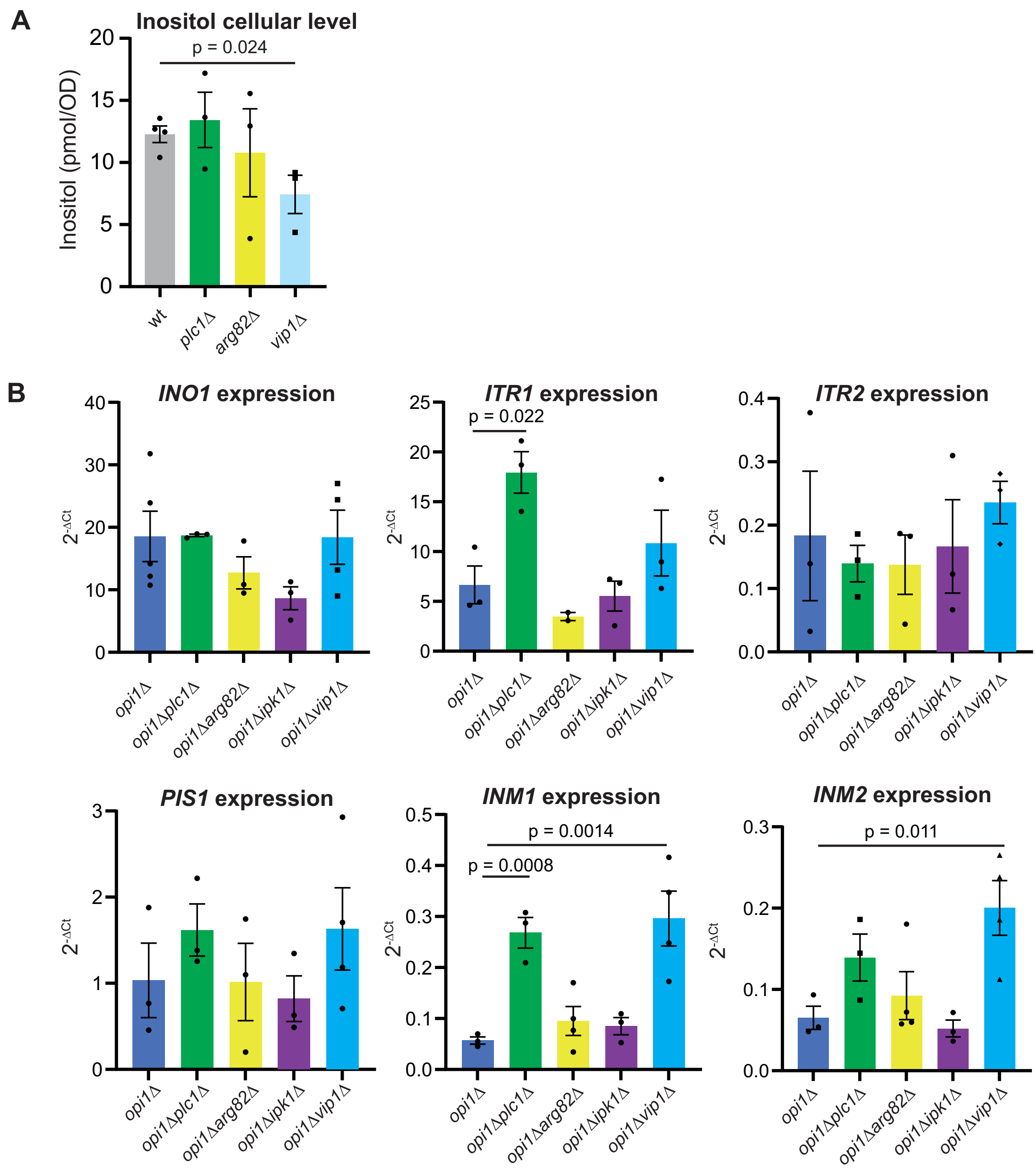

Figure EV2. Further analysis of mutants in the inositol-phosphate metabolic pathway.

(A) Intracellular levels of inositol remain unchanged in *plc1Δ* or *arg82Δ* and was reduced in *vip1Δ* cells. Wild type (yBS1), *plc1Δ* (yBS81) or *arg82Δ* (yBS92) cells were grown to mid-log phase in SC-ino media and sugar extracts were analysed by LC-MS. Graphs represent averages ± s.e.m ($n \geq 3$ biological replicates, wt vs. *vip1Δ*: $P = 0.024$, using two-tailed Student's *t*-test). **(B)** Evaluation of mRNA levels of key genes controlling inositol metabolism revealed changes in *opi1Δplc1Δ* and *opi1Δvip1Δ* yeast. Yeast strains used in Fig. 3B were grown to mid-log phase in SC-ino media. RNA extraction and RT-qPCR were performed as described in Methods. Graphs represent averages ± s.e.m ($n \geq 3$ biological replicates, *ITR1* expression in *opi1Δ* vs. *opi1Δplc1Δ*: $P = 0.022$, *INM1* expression in *opi1Δ* vs. *opi1Δplc1Δ*: $P = 0.0008$, *INM1* expression in *opi1Δ* vs. *opi1Δvip1Δ*: $P = 0.0014$, *INM2* expression in *opi1Δ* vs. *opi1Δvip1Δ*: $P = 0.011$, using two-tailed Student's *t*-test on the $\ln(2^{-\Delta Ct})$ values).

© The Author(s)

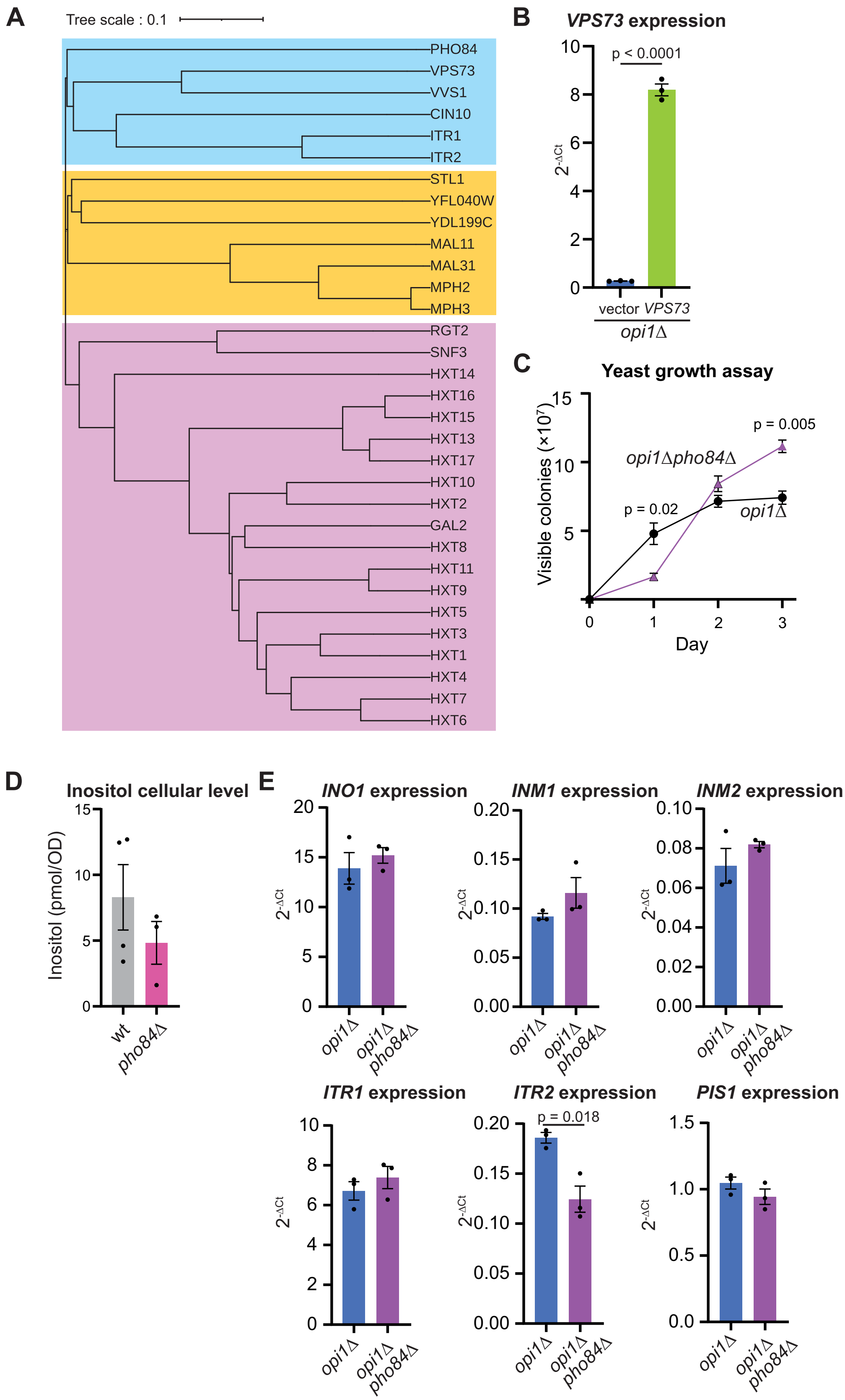
A
Tree scale : 0.1
PHO84
VPS73
VVS1
CIN10
ITR1
ITR2
STL1
YFL040W
YDL199C
MAL11
MAL31
MPH2
MPH3
RGT2
SNF3
HXT14
HXT16
HXT15
HXT13
HXT17
HXT10
HXT2
GAL2
HXT8
HXT11
HXT9
HXT5
HXT3
HXT1
HXT4
HXT7
HXT6
B
VPS73 expression
p < 0.0001
2-ΔCt
vector VPS73
opi1Δ
C
Yeast growth assay
Visible colonies (×10^7)
opi1Δpho84Δ
p = 0.005
p = 0.02
opi1Δ
Day
D
Inositol cellular level
Inositol (pmol/OD)
wt
pho84Δ
E
INO1 expression
INM1 expression
INM2 expression
ITR1 expression
ITR2 expression
p = 0.018
PIS1 expression
2-ΔCt
opi1Δ
opi1Δ pho84Δ

F

```
                                       'Motif A1'
Pho84     MSSVNKDTIHVAERSLHKEHLTEGGNMAF-----------HNHLNDFAHI-EDPLERRRL 48
LdMIT     -----------MRASV--MLCAALGGFLFGYDTGVINAALFQMKDHFGF----------- 36
hsGLUT2   ---MTEDKVTGTLVFT--VITAVLGSFQFGYDIGVINAPQQVIISHYRHVLGVPLDDRKA 55
                               :  *.: *               ..: .

Pho84     --------------ALESIDDEGFGWQQVKTISIAGVGFLTDSYDIFAINLGITMMSYVY 94
LdMIT     ---------------------SEHSWQY-------------------------------- 43
hsGLUT2   INNYVINSTDELPTISYSMNPKPTPWAEEETVAAAQL------------------ITML 96
                               .   *

                                      'Motif A1'
Pho84     WHGSMPGPSQTLLKVSTSTSVGTVIGQFGFGTLADIVGRKRIYGMELIIMIVCTILQTTVAH 154
LdMIT     ----------ALIVAIAIAGAFVGAFISGFISAAFGRRPCIAVADALFVIGSVLMGAA-- 91
hsGLUT2   W---------SLSVSSFAVGGMTASFFGGWLGDTLGRIKAMLVANILSLVGALLMGFSKL 147
                    :*      .* . . *  * : .  **     :   : :: ::*

Pho84     SPAINFVAVLTFYRIVMGIGIGGDYPLSSIITSEFATTKWRGAIMGAVFANQAWGQISGG 214
LdMIT     -PNVE---VVLVSRVIVGLAIGISSATIPVYLAEVTSPKHRGATIVLNNLFLTGGQFVAA 147
hsGLUT2   GPSHI---LIIAGRSISGLYCGLISGLVPMYIGEIAPTALRGALGTFHQLAIVTGILISQ 204
           *      ::   * : *:  *       :  .*.:    ***         . * : .
                                                                'Motif-
Pho84     IIALILVAAYKGELEYANSGAECDARCQKACDQMWRILIGLGTVLGLACL-YFRLTIPES 273
LdMIT     GFTAI------------------MVVFTSKNIGWRVAIGIGALPAVVQAFCLLFFLPES 188
hsGLUT2   IIG--------------------LEFILGNYDLWHILLGLSGVRAILQS-LLLFFCPES 242
           :                           .    *::  :*:. : .:     : : ***
B1'
Pho84     PRYQLDVNAKLELAAAAQEQDGEKKIHDTSDEDMAINGLERASTAVESLDNHPPKASFKD 333
LdMIT     PRWLLSKGHADRAKAV------------ADKFEVDLCEFQEG-------DELPSVS--ID 227
hsGLUT2   PRYLYIKLDEEVKAKQ-----SLKRLRGYDDVTKDINEMRKE-------REEASSEQKVS 290
**:                          ..    :  :..         :        .

Pho84     FCRHFGQWKYGKILL-----GTAGSWFTLDVAFYGLSLNSAVILQTIGYAGSK-NVYKKL 387
LdMIT     Y-RPLMARDMRFRVVLSSGLQIIQQFSGI----NTIMYYSSVILYDAGFRDAIMPVVLSI 282
hsGLUT2   IIQLFTNSSYRQPILVALMLHVAQQFSGI----NGIFYYSTSIFQTAGISK---PVYATI 343
  : :   .    ::         .:  :      :   *: *:   *       *  .:


                          'Motif A2'
Pho84     YDTAVGNLILICAGSLPGYWVSVFTVDIIGRKPIQLAGF---IILTALFCVIG------F 438
LdMIT     ---PLA------FMNALFTAVAIFTVDRFGRRRMLLISVFGCLVLLVVIAIIGFFIGTRI 333
hsGLUT2   ---GVG------AVNMVFTAVSVFLVEKAGRRSLFLIGMSGMFVCAIFMS-VGLVLLNKF 393
    :.         .     *::* *:   **: : * ..    ::   .:. :*       :

Pho84     AYHKLGDHGLLALYVICQFFQNFGPNTTTFIVPGECFPTRYRSTAHGISAASGKVGAIIA 498
LdMIT     SYSVGGGLFLALLAVFLA-LYAPGIGCIPWVIMGEIFPTHLRTSAASVATMANWGANVLV 392
hsGLUT2   SWMS--YVSMIAIFLFVS-FFEIGPGPIPWFMVAEFFSQGPRPAALAIAAFSNWTCNFIV 450
::       :  : ::   :   * .   :.: .* *    * :* .::: :.    .:.
                                                'Motif B2' Predicted
Pho84     QTALGTLIDHNCARDGKPTNCWLPHVMEIF-ALFMLLGIFTTLLIPETKRKTLEEINELY 557
LdMIT     SQVFPILM-GAIGV---------GGTFTIISGLMALGCIFVYFFAVETKGLTLEQIDNMF 442
hsGLUT2   ALCFQYIA-DFCGP---------Y-VFFLFAGVLLAFTLFTFFKVPETKGKSFEEIAAEF 499
   :  :     .            .: :: .::    :*. :   ***   ::*:*     :

Pho84     HDEIDPATLNFRNKNNDI------ESSSPSQLQHEA------------------------ 587
LdMIT     RKRAGLPPRFHEEGESGESGAGYREDGDLGRLATEDVCDLSSLGNRVVSFAKAEDAFTEV 502
hsGLUT2   QKKSGSAHRPKAAVEMKFLGATET------------V----------------------- 524
:.. .         :

Pho84     ---------------------------------------------  587
LdMIT     AMPDRHAVSNKFEERATSSSSDPQSLENQDEVRQAAIKAAPHEPK  547
hsGLUT2   ---------------------------------------------  524
```

Figure EV3. Sequence homology and gene expression analysis of inositol exporters in yeast.

(**A**) Phylogenetic tree of the yeast sugar transporters. Transporters with the highest sequence homology to the inositol importers Itr1 and Itr2 were highlighted in a blue box. Amino acid sequences of sugar transporters were extracted from the *Saccharomyces* genome database, and the phylogenetic tree was generated using Clustal Omega. https://www.ebi.ac.uk/jdispatcher/msa/clustalo. (**B**) RT-qPCR confirmed overexpression of *VPS73* under the ADH promoter. RNA was extracted from strains used in Fig. 4A and analysed as described in Methods. Graphs represent averages ± s.e.m ($n = 3$ biological replicates, $P < 0.0001$, using two-tailed Student's *t*-test on the $\ln(2^{-\Delta Ct})$ values). (**C**) *opi1Δpho84Δ* shows slow growth initially but ultimately has higher viability than *opi1Δ*. Yeast growth assay was performed as described in Methods. Graphs represent averages ± s.e.m ($n = 3$ biological replicates, Day 1: $P = 0.02$, Day 3: $P = 0.005$, using two-tailed Student's *t*-test). (**D**) Intracellular inositol levels were unchanged in *pho84Δ* single mutant. Wild type (yBS1) and *pho84Δ* (yNF1) yeast cells were grown in SC -ino and sugar extracts were analysed by LC-MS. Graphs represent averages ± s.e.m. ($n \geq 3$ biological replicates, not significant, using two-tailed Student's *t*-test). (**E**) Expression of genes regulating inositol homoeostasis is unchanged in *opi1Δpho84Δ* yeast, except for *ITR2*. Graphs represent averages ± s.e.m ($n = 3$ biological replicates, *ITR2* expression in *opi1Δ* vs. *opi1Δpho84Δ*: $P = 0.018$, using two-tailed Student's *t*-test on the $\ln(2^{-\Delta Ct})$ values). (**F**) Alignment of Pho84, LdMIT and hsGLUT2 showed sequence homology in the inositol binding motives. The alignment was generated using multi sequence alignment (Clustal Omega). Highlighted in green are the inositol-binding motives identified in LdMIT. Highlighted in blue is the region predicted to interact with inositol based on structural modelling.

 © The Author(s)

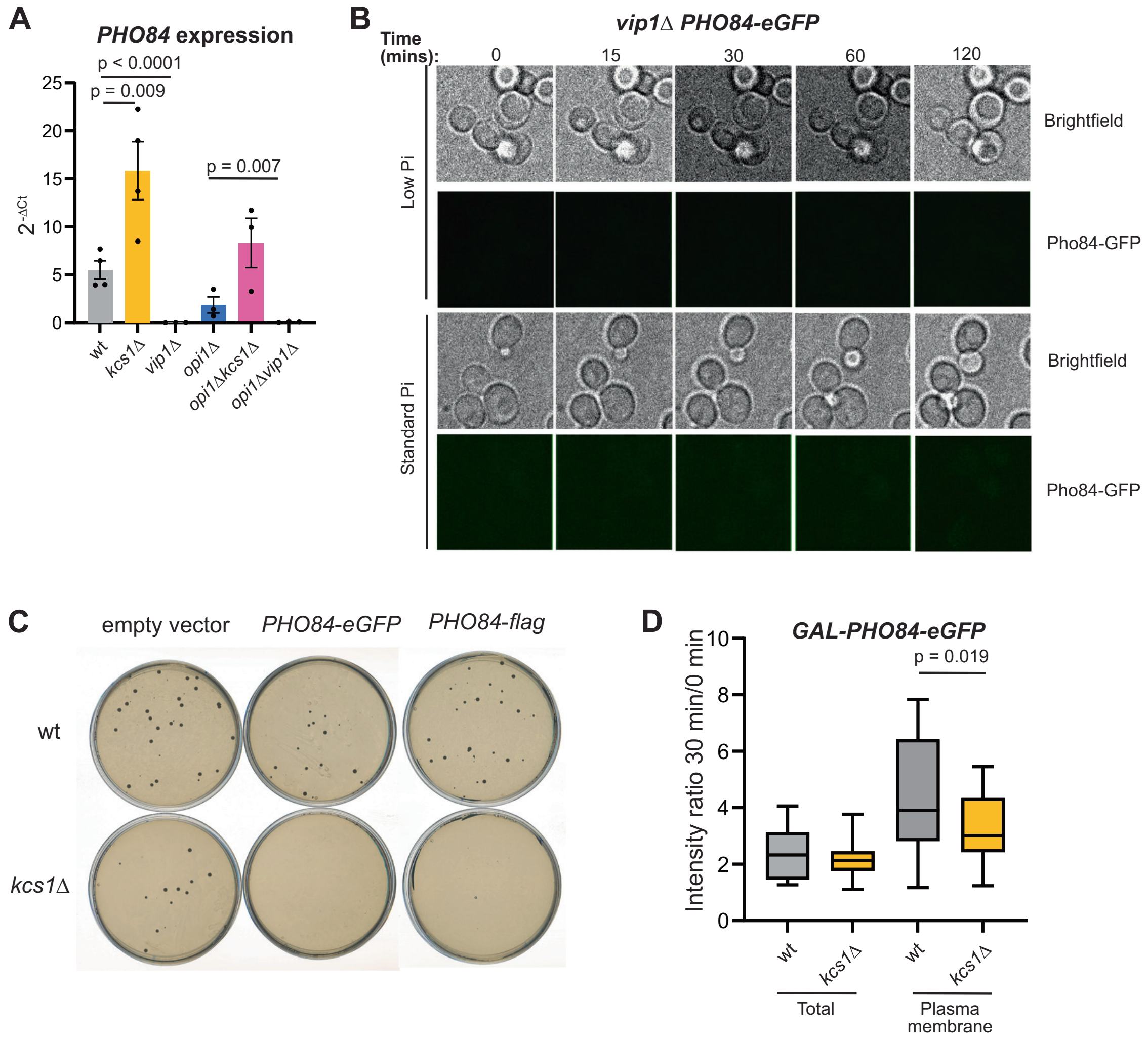

Figure EV4. Pho84 is regulated by Vip1 and Kcs1 in distinct manners.

(**A**) *PHO84* mRNA levels were upregulated in *kcs1Δ* (yBS2) and *opi1Δkcs1Δ* (yBS186), whereas they were downregulated in *vip1Δ* (yBS67) and *opi1Δvip1Δ* (yBS189). RNA was extracted from log phase cells grown in SC-ino media, and RT-qPCR was performed as described in Fig. 1D. Graphs represent averages ± s.e.m ($n \geq 3$ biological replicates, wt vs. *kcs1Δ*: $P = 0.009$, wt vs. *vip1Δ*: $P < 0.0001$, *opi1Δ* vs. *opi1Δvip1Δ*: $P = 0.007$, using two-tailed Student's *t*-test comparing $\ln(2^{-\Delta Ct})$ values). (**B**) Pho84-eGFP is undetectable in BY4741 *vip1Δ* cells (yNF10). Live imaging was performed similarly to Fig. 5C. (**C**) Additional copies of Pho84 cause lethality in *kcs1Δ* yeast. Empty vector (pRS316), *CEN-prom-PHO84-eGFP* (pBS122) and *CEN-prom-PHO84-flag* (pBS114) plasmids were transformed into BY4741 wild type (yXZ3) or *kcs1Δ* (yXZ4) and plated on SD-ura. (**D**) Gal-Pho84-eGFP signal around the plasma membrane was reduced in *kcs1Δ* yeast. Wild type and *kcs1Δ* cells transformed with pRS426-*pGAL-PHO84-eGFP* (pBS423 and pBS426) were imaged 0 and 30 min after adding 2% galactose to induce the expression of Pho84-eGFP. GFP signal intensity in the whole cell and around the cell periphery (an approximation to the plasma membrane) were measured by ImageJ, corrected for background signal and the ratio of intensity at 0 and 30 min were calculated. ~25 cells from two biological repeats were analysed and presented as a box and whisker plot. From top to the bottom of each plot: max, upper quartile, median, lower quartile, min. $P = 0.019$, using two-tailed Student's *t*-test.

© The Author(s)

A

Subject accession	Gene Name	%identity	Alignment length	E-value	Bit score	%positives
NP_001265588.1	hsSLC2A2 (GLUT2)	25.231	325	2.66e-08	58.5	40
NP_000331.1	hsSLC2A2 (GLUT2)	25.231	325	2.76e-08	59.3	40
NP_001265587.1	hsSLC2A2 (GLUT2)	25.231	325	4.41e-08	58.2	40
NP_060954.1	hsSLC22A11	23.544	412	8.49e-06	51.2	38.35
XP_047279249.1	hsSLC2A8	23.473	311	0.034	39.3	38.91
NP_003049.2	hsSLC22A2	23.21	461	2.32e-08	59.7	39.7
BAC11483.1	hsSLC22A11	23.116	398	3.40e-05	49.3	37.69
NP_003050.2	hsSLC22A4	22.611	314	1.59e-04	47.4	40.13
XP_011541892.1	hsSLC22A5	20.849	259	7.30e-04	44.7	39
XP_016865267.1	hsSLC22A5	20.849	259	0.001	44.3	39
BAD92721.1	hsSLC22A5	20.849	259	0.001	44.3	39
NP_001295051.1	hsSLC22A5	20.849	259	0.001	44.3	39

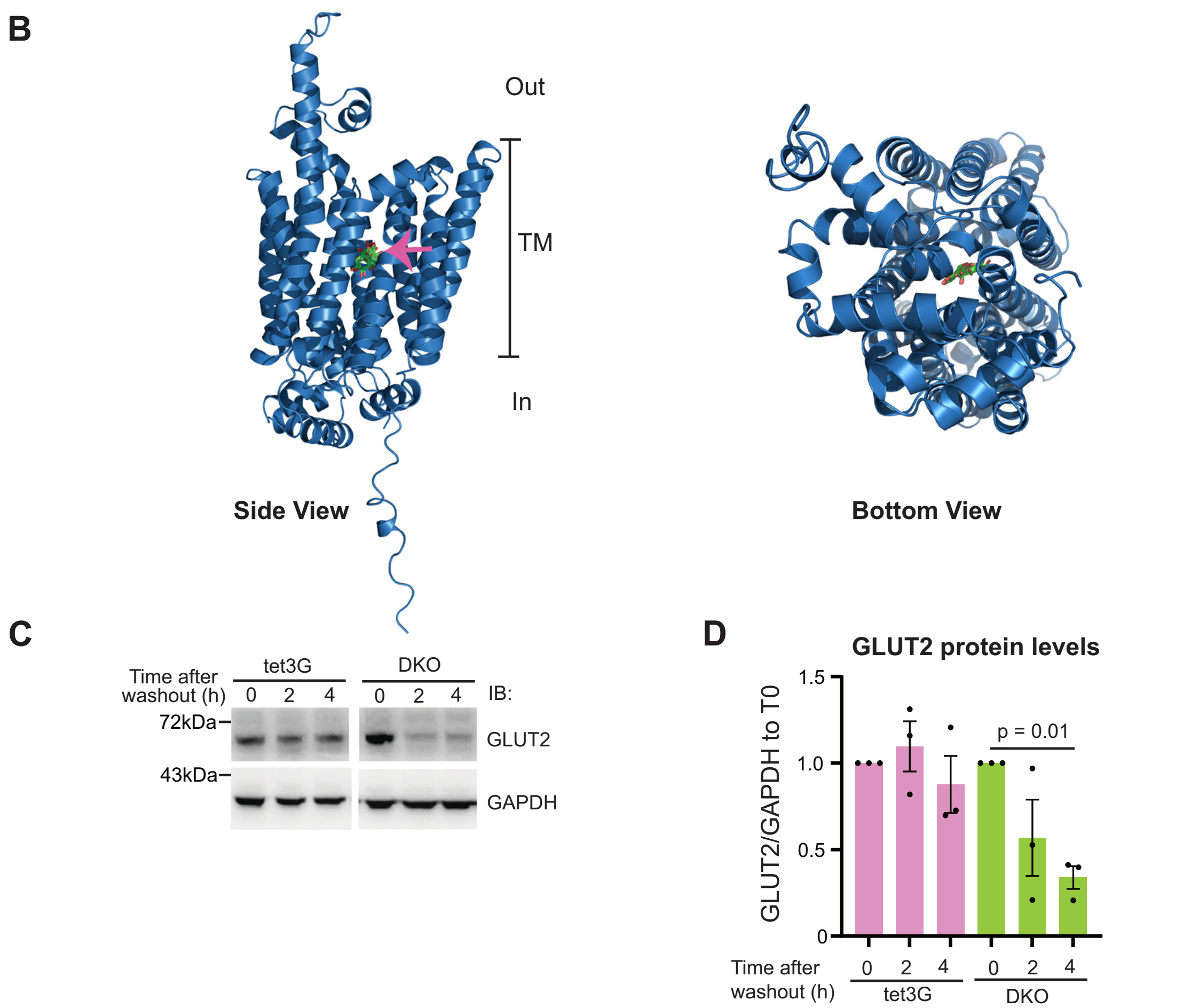

Figure EV5. In silico analysis of the human GLUT2 protein demonstrates its potential as an inositol transporter.

(**A**) A blast search of Pho84 within the human proteome revealed a number of proteins with sequence homology, with GLUT2 having the highest confidence scores. Shown is the Tree View generated by the Fast minimum evolution method. (**B**) Predicted inositol binding sites within GLUT2. Structural modelling was generated using DiffDock-L (https://neurosnap.ai). The top five inositol binding sites were found within the channel formed by the transmembrane domain, as indicated by a pink arrow. (**C**) GLUT2 protein levels are regulated by IP6K. Cells were pre-cultured in inositol-replete media and subsequently switched to inositol-free media. Protein extracts were prepared from the indicated time points and analysed by Western blotting using anti-GLUT2 or anti-GAPDH (loading control) antibodies. (**D**) Quantification of three biological repeats of (**C**). Graphs represent averages ± s.e.m ($n = 3$ biological replicates, DKO T0 vs. DKO 4 h: $P = 0.01$, using two-tailed Student's *t*-test).

 © The Author(s)
